# Illuminating Mitochondrial Dynamics: Ultrahigh Labeling Stability Probe for Long-Term SIM Super-Resolution Imaging of Mitochondria

**DOI:** 10.1021/acscentsci.5c00695

**Published:** 2025-07-29

**Authors:** Xiangpeng Lin, Xuelei Pang, Yue Huang, Xinxin Duan, Yunfei Wei, Ning Jing, Meng Zhang, Yu-Hui Zhang

**Affiliations:** † MOE Key Laboratory for Biomedical Photonics, Advanced Biomedical Imaging Facility−Wuhan National Laboratory for Optoelectronics, 12443 Huazhong University of Science and Technology, Wuhan, Hubei 430070, China

## Abstract

Delineating intricate mitochondrial dynamic changes over extended time scales through combined fluorescent probes and super-resolution microscopy is pivotal for deciphering the pathogenesis of mitochondrial-related diseases. However, a major challenge lies in the scarcity of probes that simultaneously exhibit robust labeling stability, exceptional photostability, and minimal cytotoxicity. Herein, rational design and screening yielded a novel covalent mitochondrial probe, HZ Mito Red. Due to its exceptional covalent labeling efficiency, HZ Mito Red exhibits superior mitochondrial labeling stability, with a 10-fold improvement compared to Mito Tracker Red (MTR). Furthermore, it exhibits remarkable photostability, retaining over 80% fluorescence after 300 SIM images, and negligible phototoxicity, preserving mitochondrial integrity even after 400 SIM images of continuous imaging. These advantageous properties facilitated the pioneering of high signal-to-noise, long-term dynamic SIM super-resolution imaging of mitochondria during ferroptosis, apoptosis, and autophagy, achieving unprecedented detailed delineation of mitochondrial morphology. Additionally, engineered for multichannel mitochondrial imaging, HZ Mito Deep Red mirrors the exceptional labeling stability of HZ Mito Red, achieving near-phototoxicity-free dynamic tracking with 60% fluorescence retention after 300 SIM images. Significantly, both HZ Mito Red and HZ Mito Deep Red are compatible with cell immunofluorescence staining. This study provides a robust and versatile tool for the in-depth analysis of mitochondrial dynamics in disease states.

## Graphical abstract

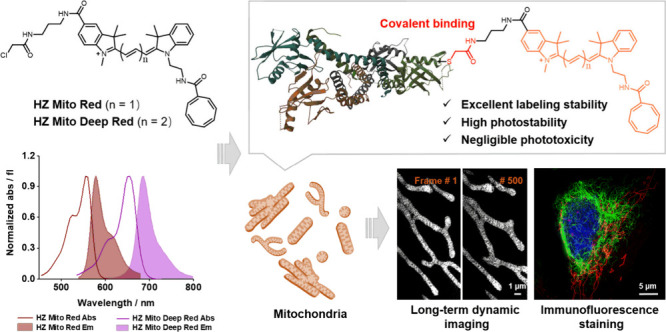

## Introduction

Mitochondria, essential bioenergetic hubs and pivotal signaling integrators, exhibit a highly dynamic nature characterized by fission, fusion, and intricate interorganellar interactions.^1–3^ These dynamic processes are fundamental to cellular homeostasis, and their dysregulation is increasingly recognized as a critical factor in a wide array of human pathologies, encompassing cancer, Alzheimer’s disease, and cardiovascular disorders.^4–8^ Consequently, long-term monitoring of mitochondrial dynamics has emerged as a crucial strategy for deciphering their multifaceted roles in disease pathogenesis. Recent advancements in live-cell super-resolution microscopy, such as structured illumination microscopy (SIM), provide unprecedented capabilities for the prolonged observation of mitochondrial dynamics, overcoming the limitations of conventional confocal techniques.^9–13^ However, leveraging these advanced imaging modalities imposes rigorous requirements on mitochondrial probes.^14,15^ First, probes must demonstrate exceptional labeling stability to ensure accurate targeting. Second, robust photostability is paramount for maintaining image quality during long-term imaging. Finally, probes must exhibit minimal phototoxicity to prevent artifacts and functional alterations during extended dynamic imaging.

To date, a rich assortment of fluorescent probes has been developed for mitochondrial imaging, and within this landscape, organic small molecules have gained preference due to their facile synthesis, customizable performance tuning, and reduced impact on cellular physiology.^16–19^ The prevailing strategy for small-molecule probes utilizes electrostatic adsorption to the mitochondrial inner membrane, an approach valued for its simplicity and demonstrated efficacy in achieving targeted localization.^20–23^ However, fluctuating mitochondrial membrane potential (MMP) significantly challenges probes that rely solely on electrostatic interactions for localization, as this mechanism is inherently dependent on the charge distribution across the mitochondrial membrane.^24,25^ This limitation can lead to significant off-target effects, ultimately manifesting as diminished signal-to-noise ratios and reduced fluorescence intensity, particularly during long-term imaging.^26,27^ Moreover, the issue is compounded by probes exhibiting high phototoxicity, which triggers structural and functional abnormalities in mitochondria, leading to rapid and substantial fluctuations in membrane potential that further exacerbate off-target effects.^28^ Functionalizing probes with lipophilic moieties to anchor in the mitochondrial membrane effectively enhances labeling stability.^12,29,30^ However, the significant reduction in aqueous solubility and disruption of the lipid microenvironment frequently induces probe dysfunction, limiting their cellular applicability. While covalent labeling strategies, as explored by Yamaguchi and co-workers, offer a potential avenue for enhanced label stability, the inherent phototoxicity associated with these probes remains a critical limitation, precluding their effective utilization in long-term dynamic studies.^31^ Consequently, the absence of ideal mitochondrial probes presents a formidable bottleneck in the advancement of long-term dynamic super-resolution imaging aimed at unraveling the intricate behaviors of mitochondria.

Here, we designed and screened eight candidate compounds, ultimately developing a novel covalent mitochondrial probe, HZ Mito Red. By leveraging covalent bond formation using a chloroacetamide group instead of relying on electrostatic adsorption, HZ Mito Red overcomes membrane potential dependency and minimizes off-target artifacts (Scheme 1a). This covalent labeling strategy imparts exceptional labeling stability, ensuring superior signal-to-noise ratios in long-term dynamic super-resolution imaging with structured illumination microscopy (SIM). HZ Mito Red also demonstrated significant photostability, retaining approximately 80% of its fluorescence across 300 SIM imaging cycles. Furthermore, phototoxicity was negligible, with well-preserved mitochondrial morphology observed after 400 SIM frames. Additionally, HZ Mito Red enables artifact-free, high signal-to-noise, long-term dynamic SIM super-resolution imaging of mitochondria during critical cell death pathways-ferroptosis, apoptosis, and autophagy—providing unprecedented visualization of mitochondrial dynamics. To achieve multichannel mitochondrial imaging, we further developed HZ Mito Deep Red. Similar to HZ Mito Red, HZ Mito Deep Red demonstrates excellent mitochondrial labeling stability and enables near-phototoxicity-free, super-resolution long-term dynamic tracking of mitochondria, with 60% fluorescence retention after 300 SIM frames. Moreover, the excellent compatibility of HZ Mito Red and HZ Mito Deep Red with immunostaining renders them highly applicable for mitochondrial imaging in fixed cells. This work provides a versatile and robust probe for in-depth studies of mitochondrial dynamics and offers a broadly applicable technical platform for advancements in mitochondrial biology.

**Scheme 1:**
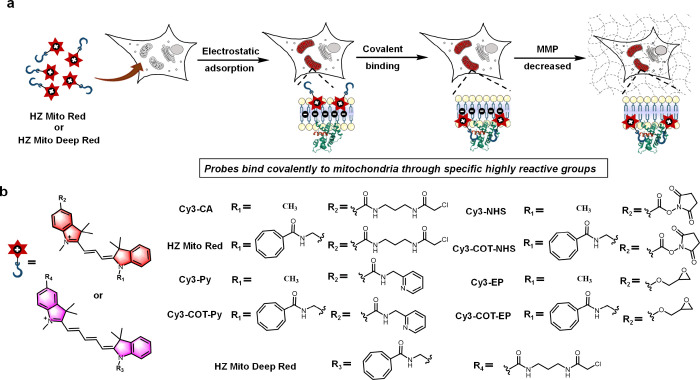
Design Strategy (a) and Structure (b) of Covalent Mitochondrial Probes

## Results

### Design of Covalent Mitochondrial Probes

Cy3 has gained prominence as a mitochondrial probe, driven by its low cytotoxicity, favorable biocompatibility, and intrinsic mitochondrial targeting capabilities.^32^ Nevertheless, its reliance on electrostatic interactions for mitochondrial targeting frequently results in undesirable off-target effects. Moreover, the inherent instability of the double bonds in Cy3 makes it highly susceptible to reactive oxygen species (ROS) generated during imaging, leading to significant photobleaching.^33^ Consequently, these limitations severely impede the application of Cy3 in demanding long-term dynamic mitochondrial imaging. To overcome these challenges, we introduced high-reactivity functional groups into Cy3 for the first time. These functional groups are capable of forming covalent bonds with nucleophilic sites on mitochondrial proteins, thereby mitigating off-target effects. Initially, asymmetric Cy3 derivatives containing carboxylic or hydroxyl groups were synthesized to provide sites for subsequent functionalization. Recognizing that the labeling efficiency of covalent mitochondrial probes is intrinsically linked to the reaction kinetics with target proteins, we systematically evaluated the compatibility of four highly reactive functional groups for Cy3-based mitochondrial labeling: (1) *N*-hydroxysuccinimide group (NHS), (2) epoxide group, (3) chloroacetamide group, and (4) pyridyl group, which is capable of undergoing protonation to form stable N–S bonds with mitochondrial membrane proteins.^13^ To improve photostability, cyclooctatetraene (COT), a widely recognized antiphotobleaching agent, was incorporated into the probe structure. This multifaceted design strategy culminated in the generation of eight distinct covalent mitochondrial probes: Cy3-CA, HZ Mito Red, Cy3-NHS, Cy3-COT-NHS, Cy3-Py, Cy3-COT-Py, Cy3-EP, and Cy3-COT-EP. Additionally, HZ Mito Deep Red was synthesized by extending the conjugated structure of HZ Mito Red to enable multicolor mitochondrial labeling (Scheme 1b). The detailed synthetic routes for all probes are shown in Scheme S1–S4, and their chemical structures were confirmed by NMR and HRMS analysis, as described in the Supporting Information.

### Spectral Properties and Mitochondrial Localization of Covalent Mitochondrial Probes

The photophysical properties of the novel covalent mitochondrial probes were examined in phosphate-buffered saline buffer (PBS, pH 7.2, Table 1). Compared to the parent fluorophore Cy3, all probes exhibited a slight red shift in both their maximum absorption and fluorescence peaks (Figure S1a, b). These shifts are attributed to the reduced energy difference (Δ*E*) between HOMO and LUMO in all covalent mitochondrial probes compared to Cy3, which leads to lower emission energy and consequently spectral red shifts (Figure S2). Notably, Cy3-EP and Cy3-COT-EP, possessing the strongly electron-donating ether groups, exhibit the smallest energy gaps and the most pronounced red-shifted emissions, with both compounds showing maximum emission wavelengths at 580 nm and corresponding Stokes shifts of 27 and 22 nm, respectively. Moreover, among the probes, HZ Mito Red achieved the highest fluorescence brightness (ε × Φ = 14400 M^–1^ cm^–1^), with a 1.7-fold increase compared to Cy3 (Table 1). Subsequently, to evaluate their mitochondrial labeling efficacy, these novel covalent probes were subjected to rigorous cellular assays. Considering the typical working concentration of Cy3-derived mitochondrial probes to be within the 250 nM to 500 nM range, U-2 OS cells were incubated with each probe at a uniform concentration (0.5 μM), and subsequently imaged by spinning disk confocal microscopy. As shown in Figure S3, under identical imaging parameters, the fluorescence intensity emanating from cells labeled with all covalent mitochondrial probes was diminished compared to Cy3. This decrease in fluorescence signal is posited to stem from the reduced hydrophobicity of the modified probes, consequently leading to lower transmembrane efficiency. To determine the working concentrations that yield fluorescence intensities comparable to Cy3 for subsequent comparative analysis, concentration gradients were established for each probe, and cell viability was assessed at the corresponding concentrations. As shown in Figure S4, intracellular fluorescence intensity exhibited a concentration-dependent increase across all probes. Specifically, when the concentrations of Cy3-CA, HZ Mito Red, Cy3-NHS, Cy3-COT-NHS, Cy3-Py, and Cy3-COT-Py were increased to 10 μM, and Cy3-EP and Cy3-COT-EP were increased to 2.5 μM, the labeling brightness was equivalent to that of 0.5 μM Cy3 (Figure S5a, b). Furthermore, we performed cell viability assessments using the chemiluminescent CellTiter-Lumi assay at various concentrations of each probe. No obvious cytotoxicity was observed, even at a concentration of 20 μM (Figure S6). Subsequently, the effect of extended incubation time with a low concentration of HZ Mito Red (0.5 μM) on intracellular fluorescence intensity was further investigated. The results showed that prolonged incubation significantly increased the average intracellular fluorescence intensity (Figure S7a). Meanwhile, no significant cytotoxicity was observed even when the incubation time was extended to 12 h (Figure S8). However, a fluorescence intensity comparable to that achieved with 10 μM HZ Mito Red after 30 min of incubation was only observed after 12 h of incubation with 0.5 μM HZ Mito Red (Figure S7b). Therefore, considering time efficiency and the absence of cytotoxicity at higher concentrations (2.5 or 10 μM), we chose a 30 min incubation time for each probe at these concentrations in subsequent experiments, rather than extending the incubation time at a lower concentration.

**Table 1:**
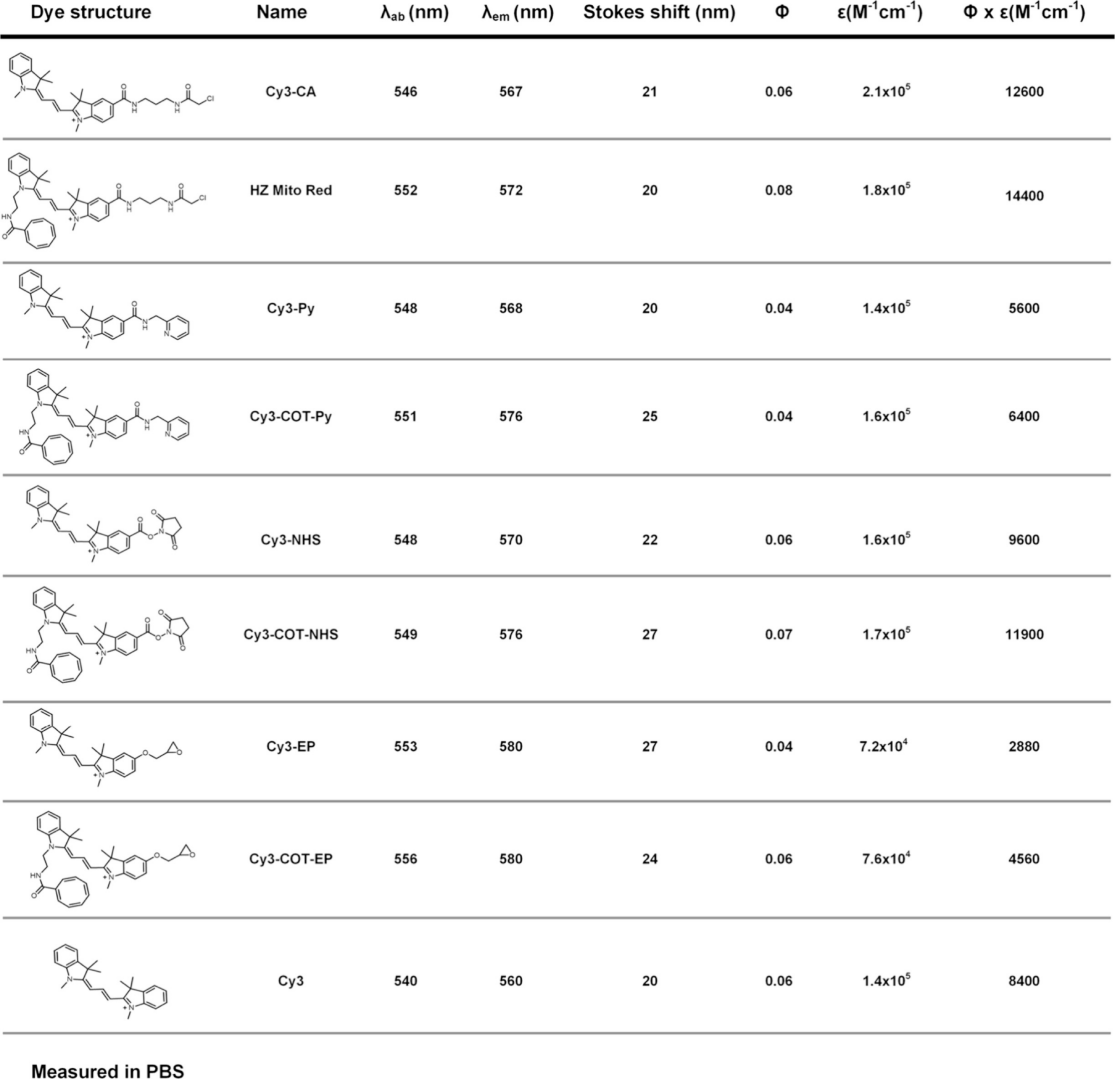
Photophysical Properties of Covalent Mitochondrial Probes and Cy3 in PBS buffer (pH 7.2)

Furthermore, to rigorously evaluate the mitochondrial labeling specificity of Cy3-CA, HZ Mito Red, Cy3-NHS, Cy3-COT-NHS, Cy3-Py, Cy3-COT-Py, Cy3-EP, and Cy3-COT-EP, colocalization studies were conducted in U-2 OS cells using Mito Tracker Deep Red (MTDR) as a reference mitochondrial marker. As shown in Figure 1a, b and Figure S9a, all probes except Cy3-COT-NHS, which exhibited a PCC of 0.5, showed a high degree of colocalization with MTDR-labeled mitochondria, with PCC values greater than 0.86. Normalized fluorescence intensity profiles further confirmed near-complete overlap between the fluorescence of Cy3-CA, HZ Mito Red, Cy3-NHS, Cy3-Py, Cy3-COT-Py, Cy3-EP, Cy3-COT-EP, and MTDR (Figure 1c, d, Figure S9b). This indicates that, with the exception of Cy3-COT-NHS, all probes effectively and specifically label mitochondria in living cells.

**Figure 1:**
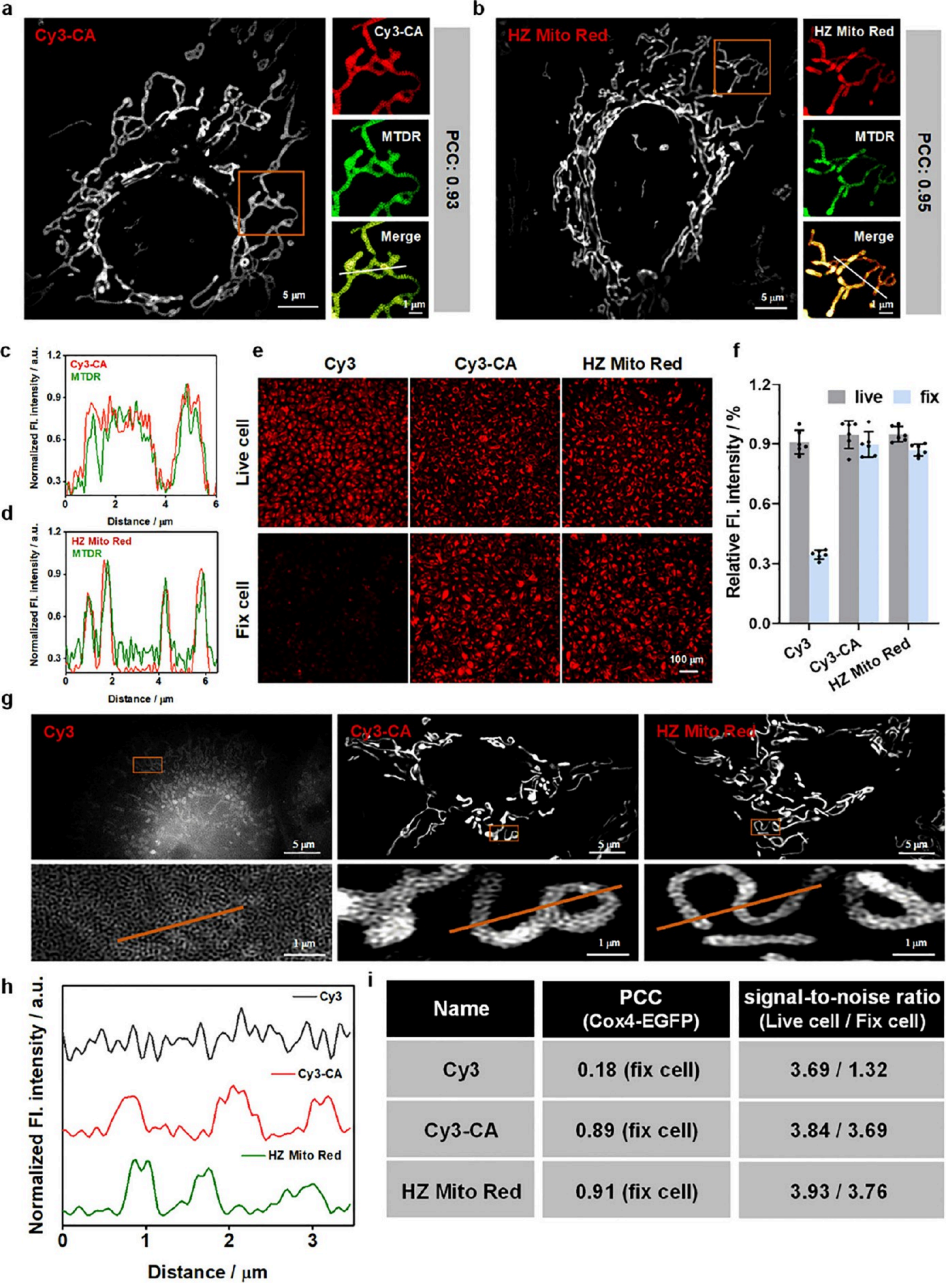
**Covalent mitochondrial labeling of Cy3-CA and HZ Mito Red.** (a, b) Colocalization analysis of Cy3-CA (a) or HZ Mito Red (b) with the commercial mitochondrial probe MTDR in live U-2 OS cells. Magnified views are from regions within the boxes. Cy3-CA and HZ Mito Red: λ_ex_ = 561 nm; λ_em_ = 609 nm. MTDR: λ_ex_ = 640 nm; λ_em_ = 667 nm. (c, d) Normalized fluorescence intensity distribution profiles of Cy3-CA and MTDR (c) and HZ Mito Red and MTDR (d) along the line in the merged channels. (e) Confocal imaging of cells stained with Cy3, Cy3-CA, or HZ Mito Red, before and after fixation. (f) Statistical analysis of relative fluorescence intensity before and after fixation in cells stained with Cy3, Cy3-CA, or HZ Mito Red. (g) Super-resolution images of cells labeled with Cy3, Cy3-CA, or HZ Mito Red after fixation. Magnified images are from regions within the boxes. (h) Normalized fluorescence intensity distribution profiles of Cy3, Cy3-CA, and HZ Mito Red along the line in fixed cells. (i) PCC and signal-to-noise ratios of Cy3, Cy3-CA, and HZ Mito Red. *n* = 6 independent experiments. All data are presented as mean ± SEM.

Stable mitochondrial labeling is essential for long-term dynamic imaging. Therefore, we further evaluated the stability of mitochondrial labeling by various probes. U-2 OS cells, prelabeled with Cy3-CA, HZ Mito Red, Cy3-NHS, Cy3-Py, Cy3-COT-Py, Cy3-EP, or Cy3-COT-EP, were subjected to glutaraldehyde (GA) fixation—a procedure that abolishes MMP and induces off-target effects for probes relying solely on electrostatic adsorption for mitochondrial targeting. In comparison to prefixation conditions, cells labeled with Cy3-NHS, Cy3-Py, Cy3-COT-Py, Cy3-EP, or Cy3-COT-EP exhibited a fluorescence intensity retention exceeding 60% postfixation, whereas Cy3-labeled control cells retained less than 35% (Figure S9c, d). Notably, Cy3-CA and HZ Mito Red outperformed the series, retaining over 85% of their initial fluorescence intensity after fixation (Figure 1e, f). To further substantiate the mitochondrial origin of fluorescence signals in fixed cells, U-2 OS cells transfected with a Cox4-EGFP plasmid were incubated with Cy3, Cy3-CA, HZ Mito Red, Cy3-NHS, Cy3-Py, Cy3-COT-Py, Cy3-EP, or Cy3-COT-EP, fixed with GA, and subjected to super-resolution imaging. As shown in Figure 1g and Figure S9e, Cy3-labeled mitochondria displayed a diffuse fluorescence signal distribution upon fixation, with the PCC to Cox4-EGFP significantly dropping to 0.18. Moreover, while all covalent mitochondrial probes exhibited enhanced labeling stability compared to Cy3, the PCC values for Cy3-NHS, Cy3-Py, Cy3-COT-Py, Cy3-EP, and Cy3-COT-EP decreased to approximately 0.7. In contrast, Cy3-CA and HZ Mito Red maintained high PCC values of 0.86 and 0.9, respectively, after fixation. (Figure 1i, Figure S10). Normalized fluorescence intensity distributions along the line in fixed cells further indicate that Cy3-CA and HZ Mito Red exhibited superior signal-to-noise ratios compared to Cy3 (Figure 1h, (i). These results indicate that all probes achieved covalent mitochondrial labeling, but Cy3-NHS, Cy3-Py, Cy3-COT-Py, Cy3-EP, and Cy3-COT-EP were less effective than Cy3-CA and HZ Mito Red. This highlights the higher reaction efficiency of Cy3 with the chloroacetamide group toward mitochondrial proteins, as well as its stronger compatibility compared to NHS group, pyridyl group, and epoxide group. In summary, Cy3-CA and HZ Mito Red achieved highly specific and stable covalent mitochondrial labeling.

### Long-Term Dynamic Super-Resolution Imaging of Mitochondria

Subsequently, we applied Cy3-CA and HZ Mito Red for long-term dynamic super-resolution imaging of mitochondria, comparing their performance with the commercial mitochondrial probe MTR and the widely recognized PK Mito Red. We began by conducting fluorescence spectroscopy on the four probes in solution. Under identical concentrations and detection parameters, all four probes exhibited virtually identical fluorescence intensities (Figure S11a). Subsequently, these probes were applied to cells, and SIM super-resolution imaging was performed under consistent parameters. Despite variations in probe concentrations within the cell culture medium during incubation, the super-resolution imaging revealed no significant differences in the average intracellular fluorescence intensity, suggesting that the intracellular concentrations of the four probes were remarkably consistent (Figure S11b). The subsequent results of mitochondrial dynamics imaging are presented in Figure 2a, where after capturing 50 frames of super-resolution images, MTR exhibited significant background signals resembling the endoplasmic reticulum (ER), which intensified with increasing frame count. This phenomenon is attributed to MTR’s reliance on electrostatic adsorption for mitochondrial labeling, leading to off-target effects during long-term dynamic super-resolution imaging. This issue is further exacerbated by its inherent phototoxicity, which induces mitochondrial dysfunction. Normalized fluorescence intensity distribution along the line in Sec61β-mEGFP-expressing cells revealed that the off-target MTR accumulated on the ER due to its high lipophilicity. In comparison, PK Mito Red only showed faint background signals after capturing 200 frames, owing to its lower phototoxicity. However, as image acquisition continued, its accumulation in the ER progressively increased, similar to MTR. In striking contrast, Cy3-CA and HZ Mito Red exhibited a complete absence of discernible background signals even after acquiring 500 frames. Normalized fluorescence intensity distribution analyses in merged channels further affirmed the exceptional signal-to-noise ratio afforded by Cy3-CA and HZ Mito Red. These results indicate that both Cy3-CA and HZ Mito Red enable long-term dynamic mitochondrial super-resolution imaging, with labeling stability more than 10 times higher than that of MTR. Furthermore, to quantify photobleaching resistance, we statistically analyzed alterations in average fluorescence intensity during long-term dynamic imaging. As shown in Figure 2b, Cy3-CA exhibited the most rapid photobleaching kinetics. Conversely, HZ Mito Red and PK Mito Red displayed the lowest photobleaching rates, retaining over 60% of their fluorescence intensity after continuous acquisition over 500 frames. Intriguingly, the pronounced phototoxicity of MTR triggered cellular release of the probe, leading to an initial rapid increase in cellular brightness within the first 20 frames, followed by accelerated photobleaching. However, due to off-target effects, the retained fluorescence signal within the cells was not entirely from the mitochondria. Therefore, we further analyzed the changes in average fluorescence intensity specifically within the mitochondrial region, revealing comparable rapid photobleaching kinetics for Cy3-CA and MTR, with a 20% reduction in mitochondrial fluorescence intensity after approximately 70 and 50 frames, respectively. HZ Mito Red and PK Mito Red maintained 80% of their fluorescence intensity after acquiring nearly 250 frames (Figure 2c, d). Crucially, beyond 250 frames, HZ Mito Red demonstrated superior photostability compared to PK Mito Red. Additionally, the phototoxicity was further quantified by comparing the full width at half-maximum (fwhm) of the fluorescence intensity distribution across mitochondrial widths (Figure S12a, b). On average, HZ Mito Red and PK Mito Red were able to provide ∼400 frames of super-resolution imaging before the mitochondrial width expanded to 125% of its original size, whereas Cy3-CA and MTR could only provide ∼200 frames and 80 frames, respectively, under the same conditions (Figure 2e). These comprehensive results unequivocally establish that HZ Mito Red can robustly label mitochondria during protracted dynamic imaging, showcasing exceptional photobleaching resistance and minimal phototoxicity. Furthermore, HZ Mito Red demonstrates broad compatibility, enabling mitochondrial labeling across multiple cell lines (Figure S13). In summary, HZ Mito Red stands out as a superior probe for stable mitochondrial labeling in long-term dynamic imaging applications.

**Figure 2:**
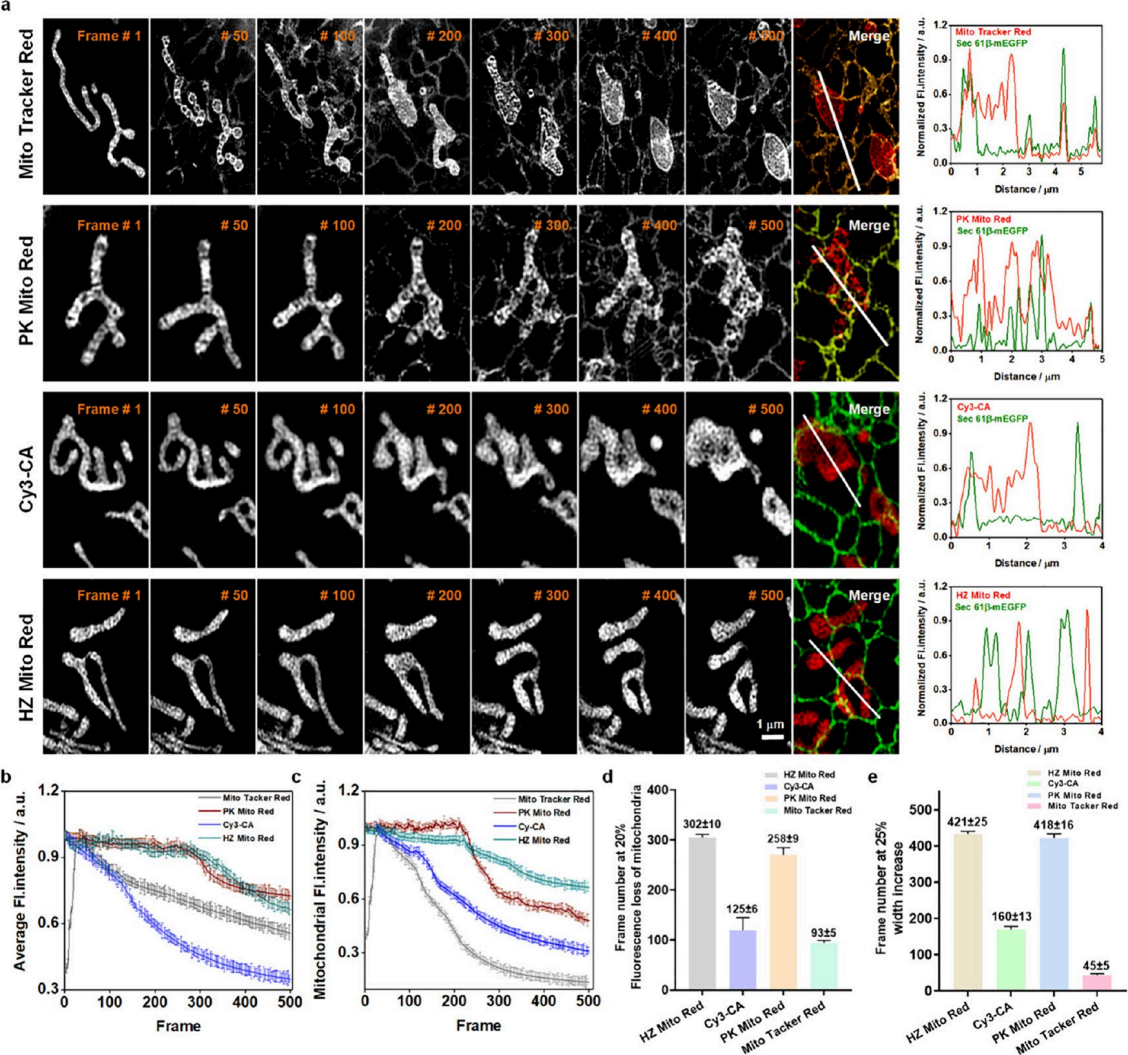
**Comparison of the performance of different probes in long-term dynamic super-resolution imaging of mitochondria.** (a) Long-term dynamic super-resolution imaging of mitochondria was performed in Sec61β-mEGFP transfected U-2 OS cells stained with MTR, PK Mito Red, Cy3-CA, or HZ Mito Red. And normalized fluorescence intensity distribution profiles along the line in merged channels. Images were acquired continuously without time intervals, and contrast was enhanced using ImageJ to compensate for fluorescence loss over time. (b, c) Changes in average fluorescence intensity (b) and mitochondrial fluorescence intensity (c) in long-term dynamic super-resolution images. (d) Average frame number at 20% fluorescence loss. (e) Average frame number at 25% increase in mitochondrial width. MTR, PK Mito Red, Cy3-CA, and HZ Mito Red were all imaged under the same conditions: λ_ex_ = 561 nm; λ_em_ = 609 nm. *n* = 15 cells from three independent experiments. All data are presented as mean ± SEM.

### Long-Term Dynamic Super-Resolution Imaging of Mitochondria during Ferroptosis, Apoptosis and Autophagy

Ferroptosis, apoptosis, and autophagy represent distinct modes of programmed cell death, wherein mitochondria assume a pivotal regulatory role.^34–36^ Long-term dynamic monitoring of mitochondrial behavior is essential for gaining deeper insights into the mechanisms of cell death.^37,38^ However, the loss of MMP, coupled with the fact that these processes often span tens of minutes to several hours, makes long-term dynamic imaging of mitochondria highly challenging. Herein, we employed HZ Mito Red to pioneer long-term super-resolution imaging of mitochondrial dynamics during ferroptosis, apoptosis, and autophagy, which were induced using Erastin, Oligomycin A (OA), and CCCP, respectively (Figure 3a). As a control, PK Mito Red was initially used to track mitochondrial dynamics during autophagy using SIM, acquiring super-resolution images every 1 min. As shown in Figure 3b, a significant increase in background signal was observed by 20 min, and the imaging signal-to-noise ratio gradually decreased as autophagy progressed, eventually resulting in loss of mitochondrial localization (Figure 3c). In contrast, cells labeled with HZ Mito Red achieved high signal-to-noise ratio dynamic tracking of mitochondria during autophagy, apoptosis, and ferroptosis under the same imaging conditions. As shown in Figure 3d, no discernible changes in mitochondrial morphology were detected within the initial 20 min of autophagy. Between 20 and 140 min, mitochondria exhibited significant swelling and sustained contraction. Additionally, during apoptosis, mitochondria gradually fragmented and contracted within 30–70 min post-OA induction, culminating in spherical structures (Figure 3f). Analogously, mitochondria maintained their normal morphology in the first 30 min of ferroptosis (Figure 3h). Subsequently, from 30 to 180 min, the mitochondria divided into shorter fragments and gradually underwent vacuolization. Long-term dynamic imaging provides a detailed temporal profile of mitochondrial morphological alterations across diverse death models, a capability that surpasses the limitations of traditional single-frame fluorescence imaging. This underscores that long-term dynamic imaging offers more comprehensive and nuanced insights into the intricate processes of mitochondrial structural remodeling. Moreover, analysis of the fluorescence intensity distribution along the line at 140 min (autophagy), 70 min (apoptosis), and 180 min (ferroptosis) confirmed the absence of significant off-target signal for HZ Mito Red, thereby demonstrating exceptional imaging signal-to-noise ratios (Figure 3e, g, and i). Furthermore, to preclude the possibility that alterations in mitochondrial morphology were attributable to phototoxicity during imaging, control super-resolution imaging of unstimulated cells was performed under identical conditions, revealing sustained mitochondrial morphology over a 3 h period (Figure S14). In summary, these results demonstrate that HZ Mito Red is not limited by MMP and enables high signal-to-noise ratio, long-term dynamic imaging of mitochondria during ferroptosis, apoptosis, and autophagy, providing more detailed information on mitochondrial morphological dynamics.

**Figure 3:**
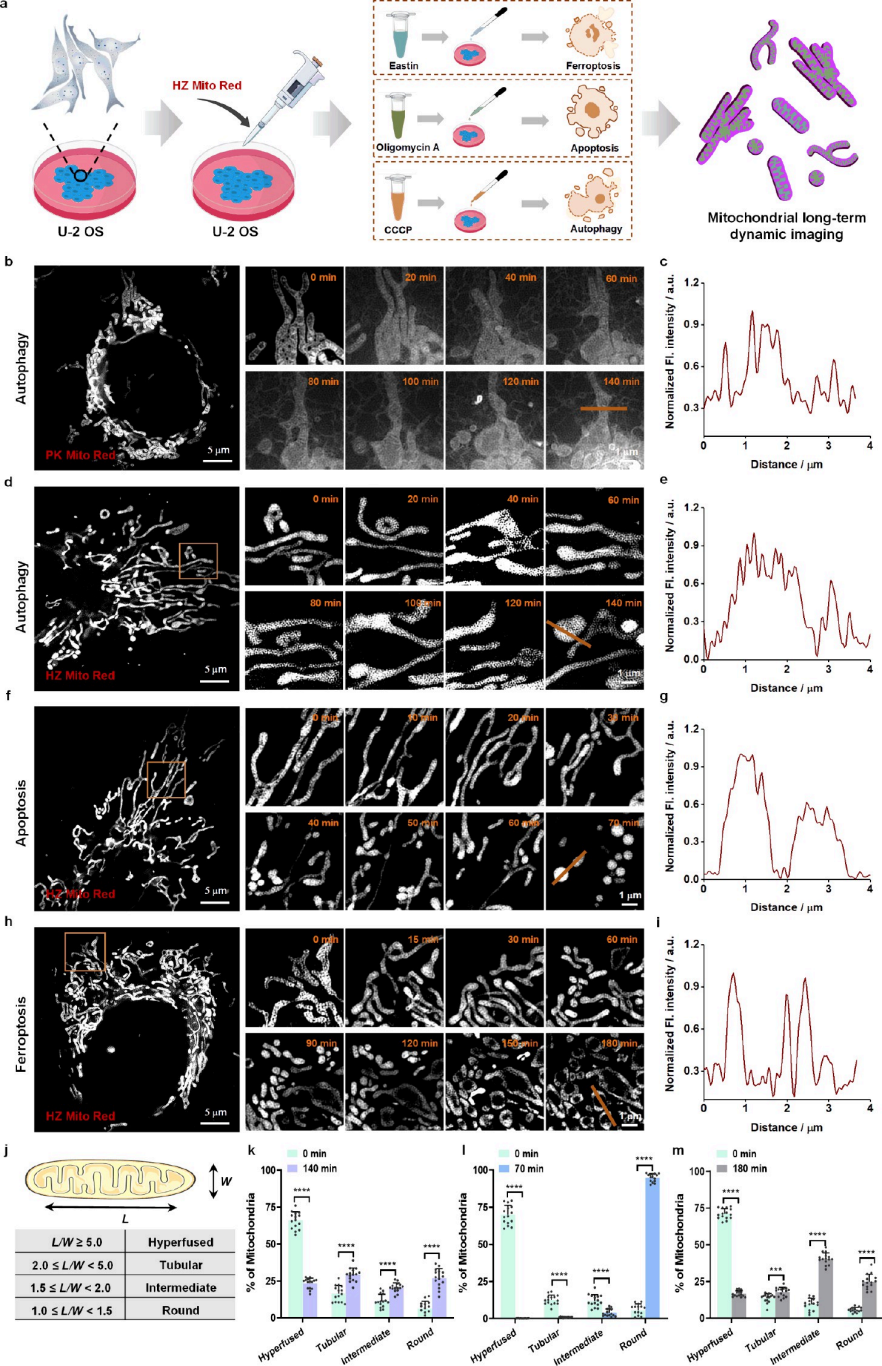
**Long-term dynamic imaging of mitochondria in different types of cell death.** (a) Schematic diagram of sample preparation and imaging process. (b) Long-term dynamic super-resolution imaging of mitochondria labeled with PK Mito Red during autophagy. (c) Normalized fluorescence intensity distribution along the line in (b) at 140 min. (d, f, h) Long-term dynamic super-resolution imaging of mitochondria during autophagy (d), apoptosis (f), and ferroptosis (h). (e, g, i) Normalized fluorescence intensity distribution along the line in images at 140 min for autophagy (e), 70 min for apoptosis (g), and 180 min for ferroptosis (i). (j) Classification of mitochondrial morphology. (k, l, m) Quantitative statistical analysis of mitochondrial morphology before and after autophagy (k), apoptosis (l), and ferroptosis (m). *n* = 15 cells from three independent experiments. All data are presented as mean ± SEM. Statistical significance: *** denotes *P* < 0.001; **** denotes *P* < 0.0001.

Finally, mitochondrial morphology was quantitatively analyzed across these three cellular demise processes using the established length-to-width ratio (*L*/*W*) methodology, classifying mitochondrial shapes into four categories: round (1.0 < *L*/*W* < 1.5), intermediate (1.5 < *L*/*W* < 2.0), tubular (2.0 < *L*/*W* < 5.0), and hyperfused (*L*/*W* > 5.0) (Figure 3g). Automated image analysis using ImageJ software was employed to statistically evaluate mitochondrial morphology. During autophagy, the proportion of elongated mitochondria decreased, while round, intermediate, tubular, and hyperfused mitochondrial morphologies became more uniformly distributed (Figure 3k). In apoptosis, mitochondria underwent progressive shortening, with over 90% adopting a spherical morphology by the late stage (Figure 3l). During ferroptosis, mitochondria exhibited a transition toward shorter and rounder forms, with significant shifts in the proportions of round, tubular, and hyperfused morphologies relative to preferroptosis conditions (Figure 3m).

### HZ Mito Deep Red Mediated Multichannel Imaging of Mitochondria

Finally, to facilitate multicolor mitochondrial imaging, HZ Mito Deep Red was synthesized by extending the π-conjugated system of HZ Mito Red. Photophysical characterization of HZ Mito Deep Red in DMSO revealed maximum excitation and emission wavelengths at 655 and 690 nm, respectively, yielding a Stokes shift of 35 nm (Figure 4a, Figure S15). Additionally, the solubility of HZ Mito Red and HZ Mito Deep Red in PBS buffer (pH 7.2) was further determined to be 3.4 and 2.0 mg/mL, respectively. HZ Mito Deep Red was then applied for mitochondrial labeling in live cells. Surprisingly, at a concentration of 1 μM, the labeling brightness of HZ Mito Deep Red was comparable to that of 10 μM HZ Mito Red (Figure S16a, b). This exceptional brightness is attributed to the intrinsic fluorescence properties of HZ Mito Deep Red and its expanded π-conjugated backbone, which mitigates the influence of the chloroacetamide group on the probe’s hydrophobicity. Moreover, HZ Mito Deep Red demonstrated negligible cytotoxicity at working concentrations (Figure S17). Next, the mitochondrial targeting specificity of HZ Mito Deep Red in live cells was investigated (Figure 4b). Colocalization assays with the commercial Mito Tracker Green (MTG) affirmed a high degree of overlap between HZ Mito Deep Red and MTG fluorescence, evidenced by a PCC of 0.92. Subsequently, HZ Mito Deep Red was used to label mitochondria in cells transfected with the Cox4-EGFP plasmid. After fixation and disruption of MMP, HZ Mito Deep Red and EGFP fluorescence still exhibited strong overlap, with a PCC of 0.88 (Figure 4c). These results demonstrate that HZ Mito Deep Red can specifically and covalently label mitochondria while maintaining excellent labeling stability. Further application of HZ Mito Deep Red to long-term dynamic imaging of mitochondria revealed its superior performance. Upon acquiring 500 consecutive frames, HZ Mito Deep Red exhibited a complete absence of discernible background signal (Figure 4d). In contrast, MTDR displayed a substantial increase in background signal after a mere 100 frames. Normalized fluorescence intensity distribution analysis along the line also indicated that HZ Mito Deep Red provided superior imaging signal-to-noise ratio after capturing 500 frames (Figure 4e). Furthermore, quantitative analysis of fluorescence intensity alterations within mitochondrial regions revealed the enhanced photostability of HZ Mito Deep Red, demonstrating superior retention of mitochondrial fluorescence throughout 500 consecutive frames compared to MTDR (Figure 4f). To assess the phototoxicity of HZ Mito Deep Red, alterations in mitochondrial width during imaging were evaluated. Mitochondria stained with HZ Mito Deep Red maintained their native morphology for up to 300 frames, whereas MTDR-stained mitochondria exhibited swelling within the initial 20 frames (Figure 4g). On average, HZ Mito Deep Red allowed continuous imaging of 491 frames before mitochondrial width increased to 120% of the original, whereas MTDR provided only 40 frames under the same conditions, indicating the low phototoxicity of HZ Mito Deep Red (Figure 4h). Finally, the labeling efficacy of HZ Mito Deep Red across diverse cell lines was evaluated. As shown in Figure 4i, PCC between HZ Mito Deep Red and MTR fluorescence consistently exceeded 0.89 in COS-7 cells, BHK21 cells, cardiomyocytes cells and A2780 cells, demonstrating the broad applicability of HZ Mito Deep Red for specific mitochondrial labeling across a range of cell lines. In conclusion, HZ Mito Deep Red emerges as a powerful tool for specific, stable, and low-phototoxic mitochondrial labeling, particularly advantageous for multicolor and long-term dynamic imaging applications.

**Figure 4:**
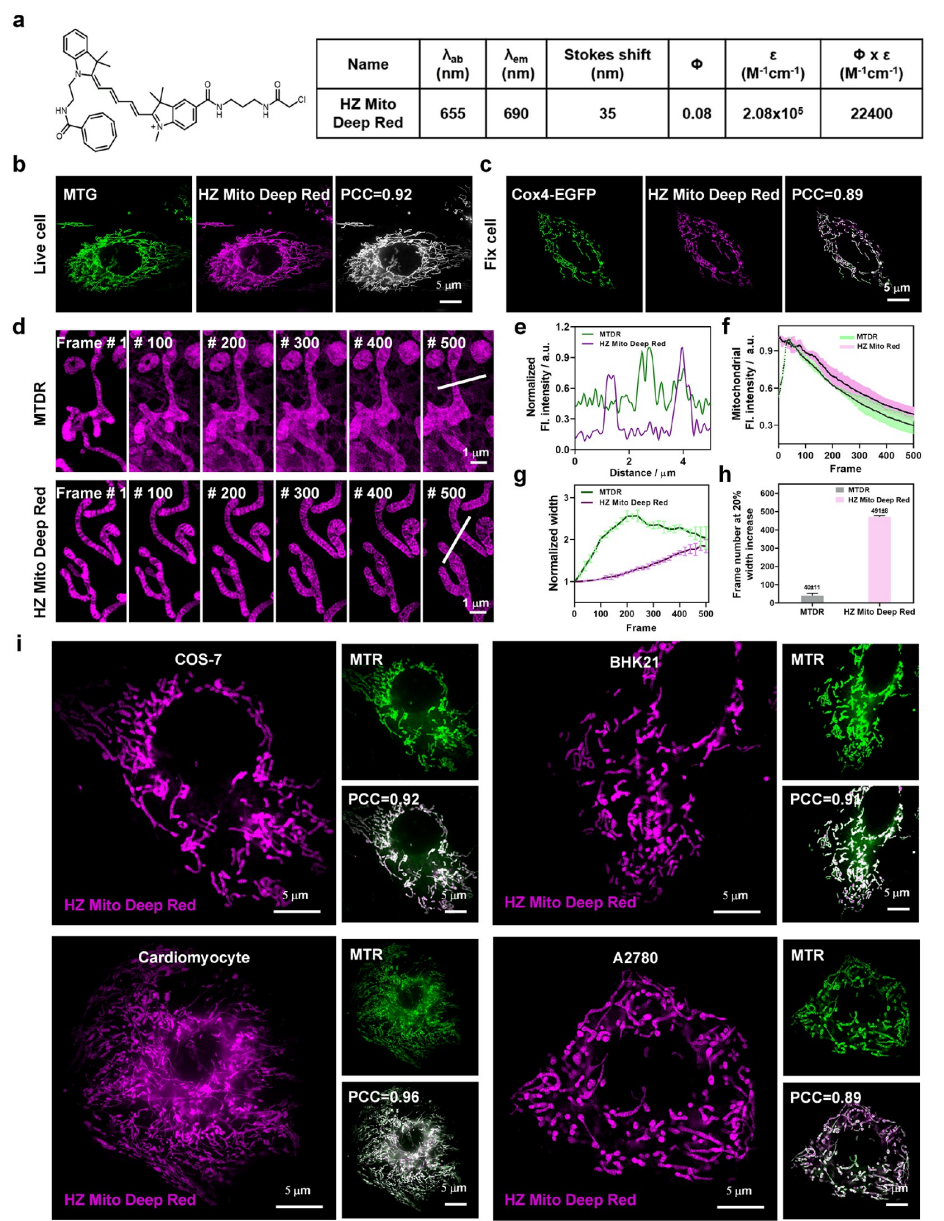
**HZ Mito Deep Red enables multicolor covalent labeling of mitochondria.** (a) Structure and photophysical properties of HZ Mito Deep Red. (b) Colocalization of HZ Mito Deep Red and MTG in live cells. (c) Colocalization of HZ Mito Deep Red with Cox4-EGFP in fixed cells. (d) Long-term dynamic super-resolution imaging of mitochondria labeled with MTDR and HZ Mito Deep Red. ImageJ was used to enhance contrast to compensate for fluorescence intensity loss during long-term imaging. (e) Normalized fluorescence intensity distribution along the line in the 500th frame image. (f) Changes in mitochondrial fluorescence intensity in long-term dynamic super-resolution images (*n* = 10). (g) Normalized internal width distribution of mitochondria in long-term dynamic super-resolution images (*n* = 6). (h) Average frame number when mitochondrial width increased by 25%. (i) Colocalization analysis of mitochondrial labeling by HZ Mito Deep Red across multiple cell lines. HZ Mito Deep Red: λ_ex_ = 640 nm; λ_em_ = 667 nm. MTG: λ_ex_ = 488 nm; λ_em_ = 525 nm. Cox4-EGFP: λ_ex_ = 488 nm; λ_em_ = 525 nm. MTR: λ_ex_ = 561 nm; λ_em_ = 607 nm. All data are presented as mean ± SEM.

### HZ Mito Red and HZ Mito Deep Red Are Compatible with Immunofluorescence Staining

Immunofluorescence staining is a ubiquitous technique for fluorescently tagging target proteins, typically entailing chemical fixation of samples followed by incubation with fluorophore-conjugated antibodies or toxins for specific labeling.^39–41^ Given the robust mitochondrial labeling proficiency of HZ Mito Red and HZ Mito Deep Red in fixed cells, we proceeded to investigate their compatibility with immunofluorescence staining. Cells prelabeled with HZ Mito Red or HZ Mito Deep Red were fixed with GA, permeabilized with Triton X-100, and subsequently immunostained with antibodies targeting diverse proteins or protein-binding toxins (Figure 5a). In fixed U-2 OS cells, immunostaining with Anti-Vimentin-Alexa488 visualized both mitochondria and intermediate filaments (IF) (Figure 5b). Additionally, three-color fluorescence labeling of nucleus, mitochondria, IF, actin and tubulin was achieved across multiple cell lines, including BHK21 cells, cardiomyocytes cells and COS-7 cells (Figure 5c–h). This demonstrates that the combination of HZ Mito Red or HZ Mito Deep Red with immunofluorescence staining enables comprehensive mapping of spatial relationships between mitochondria and a diverse array of subcellular architectures across various cell types.

**Figure 5:**
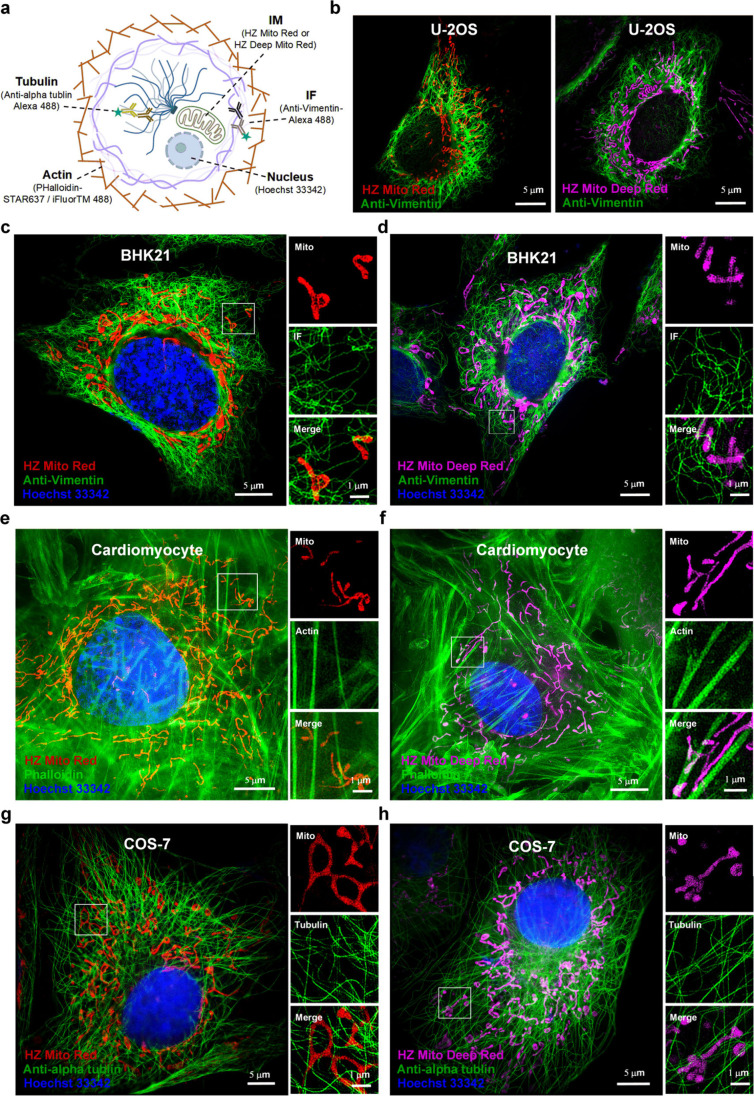
**Multicolor super-resolution imaging of HZ Mito Red and HZ Mito Deep Red combined with immunolabeling.** (a) Immunofluorescence staining strategy for multicolor imaging in fixed cells. (b) Two-color super-resolution imaging of mitochondria and IF in fixed U-2 OS cells. (c–h) Three-color super-resolution imaging of fixed BHK21 cells, Cardiomyocyte cells and COS-7 cells, revealing mitochondria, nucleus, IF, actin and tubulin. Magnified images correspond to the areas demarcated by the boxes. HZ Mito Red: λ_ex_ = 561 nm; λ_em_ = 609 nm. HZ Mito Deep Red: λ_ex_ = 640 nm; λ_em_ = 667 nm. Anti-Vimentin, Phalloidin and Antialpha tublin: λ_ex_ = 488 nm; λ_em_ = 525 nm. Hoechst33342: λ_ex_ = 405 nm; λ_em_ = 450 nm.

## Discussion

The current absence of mitochondrial fluorescent probes that simultaneously achieve high labeling stability, robust photobleaching resistance, and minimal toxicity severely impedes in-depth research into mitochondrial diseases.^42,43^ In this study, we conducted a pioneering systematic screening of the compatibility between highly reactive functional groups and dyes, resulting in the development of HZ Mito Red, a novel covalent mitochondrial probe. The high compatibility of its chloroacetamide group with Cy3 significantly enhanced mitochondrial labeling stability, as evidenced by continuous acquisition of 500 SIM super-resolution frames with no discernible background signal. HZ Mito Red demonstrated an order-of-magnitude increase in labeling stability over MTR, and a 2.5-fold improvement over PK Mito Red, ensuring high signal-to-noise ratio for long-term dynamic SIM super-resolution imaging. In addition, HZ Mito Red also exhibits excellent resistance to photobleaching and low phototoxicity, retaining 80% of its fluorescence intensity after approximately 300 consecutive SIM frames, with mitochondrial width increasing by less than 25%. The nearly 3-fold enhancement in photobleaching resistance and 10-fold reduction in phototoxicity, compared to MTR, are critical for achieving high-quality, sustained mitochondrial tracking.

Using HZ Mito Red, we achieved long-term dynamic SIM super-resolution imaging of mitochondria during ferroptosis, apoptosis, and autophagy. During these processes, the dissipation of MMP prevents the use of probes that rely on electrostatic adsorption for mitochondrial labeling, making long-term dynamic imaging of mitochondria a challenging task. HZ Mito Red, with its high labeling stability, low cytotoxicity, and excellent photobleaching resistance, effectively addresses these challenges. Using high signal-to-noise ratio super-resolution images, we captured the dynamic changes of mitochondria during ferroptosis, apoptosis, and autophagy for the first time, providing unprecedented details of mitochondrial morphological dynamics and statistically analyzed the characteristic morphological changes of mitochondria in different types of cell death.

To enable multichannel mitochondrial imaging, HZ Mito Deep Red was further developed. The expansion of the spectral coverage enriches the optical toolbox for multicolor imaging, providing more support for the multiparametric analysis of complex biological systems. Taken together, HZ Mito Red and HZ Mito Deep Red represent the first dual-color covalent mitochondrial probe pair with good hydrophilicity, which simultaneously fulfills the key criteria of high labeling stability, excellent photostability, and minimal phototoxicity. Moreover, HZ Mito Red and HZ Mito Deep Red also demonstrate, for the first time, compatibility with both long-term dynamic SIM super-resolution imaging in living cells and fluorescence immunostaining techniques in fixed cells-achievements unattainable with probes relying solely on electrostatic and hydrophobic interactions for mitochondrial labeling. In summary, this study provides a powerful tool for long-term dynamic monitoring of mitochondria and offers strong technical support for investigating the pathogenesis of mitochondrial-related diseases.

## Experimental Section

### 1 Materials and Experimental Instruments

Reagents were purchased of the highest commercial quality and used without further purification unless otherwise stated. Unless otherwise stated, all reactions were carried out in a nitrogen atmosphere with dry solvents under anhydrous conditions. Nuclear magnetic resonance (NMR) spectra (^1^H NMR and ^13^C NMR) were obtained on a 400 or 600 MHz spectrometer (Bruker, Switzerland). High-resolution mass spectrometric data were obtained using an 1100 LC/MSD Trap 2D liquid chromatography-ion trap mass spectrometer (LC-MS) (Agilent, USA). UV–vis absorption spectra of sample solutions in spectral grade solvents were measured using an Agilent Cary 60 UV–vis spectrophotometer in a 1 cm square quartz cuvette. Emission spectra were measured using an Agilent Cary Eclipse fluorescence spectrophotometer. The CellTiter-Lumi assay was obtained on a VictorX4 (PerkinElmer, USA). The living-cell confocal imaging was performed using a spinning disk confocal microscope (Olympus) equipped with an UltraVIEW VoX 3D live-cell imaging system (PerkinElmer), sCMOS camera, and four-channel excitation lasers (405, 488, 561, and 640 nm). SIM imaging was performed with a high-sensitivity structured illumination microscope (His-SIM, Guangzhou Computational Super-Resolution Biotech Co., Ltd.). All the super-resolution images were reconstructed from 9 raw images. Fiji software was used to analyze the confocal and super-resolution images.

### 2 Synthesis of Covalent Mitochondrial Probes

The structures of Cy3-CA, HZ Mito Red, Cy3-NHS, Cy3-COT-NHS, Cy3-Py, Cy3-COT-Py, Cy3-EP, Cy3-COT-EP and HZ Mito Deep Red are illustrated in Scheme. S1–S4. The Supporting Information provide detailed information on the synthetic routes and methods of compounds, as well as the NMR spectroscopy and mass spectrometry.

### 3 Test Solution Preparation and Spectral Measurement

Stock solutions of Cy3, Cy3-CA, HZ Mito Red, Cy3-NHS, Cy3-COT-NHS, Cy3-Py, Cy3-COT-Py, Cy3-EP, Cy3-COT-EP and HZ Mito Deep Red (1 mM, 10 mL) were individually prepared in DMSO. A 20 μL of the stock solution was taken and diluted with PBS buffer (pH 7.2) to prepare a 10 μM test solution. The UV absorption spectrum of the probe was measured from 400 to 650 nm using an Agilent Cary 60 UV–vis spectrophotometer, while the fluorescence emission spectrum, excited at 520 nm and recorded from 540 to 680 nm, was obtained using an Agilent Cary Eclipse fluorescence spectrophotometer.

### 4 Fluorescence Quantum Yield and Molar Extinction Coefficient

The fluorescence quantum yield (Φ_*f*_) of the sample was calculated using the following formula, with Rhodamine B in PSB (Φ = 0.31) as the reference:

$$\frac{\Phi_{sample}}{\Phi_{ref}}=\frac{OD_{ref}\times I_{sample}\times {d^{2}}_{sample}}{OD_{sample}\times I_{ref}\times {d^{2}}_{ref}}$$

Φ: quantum yield of fluorescence.

*I*: integrated emission intensity.

OD: optical density at the excitation wavelength.

*d*: refractive index of solvents: *d*_PBS_ = 1.336.

The molar extinction coefficients of Cy3, Cy3-CA, HZ Mito Red, Cy3-NHS, Cy3-COT-NHS, Cy3-Py, Cy3-COT-Py, Cy3-EP, Cy3-COT-EP and HZ Mito Deep Red in DMSO were measured using an Agilent Cary 60 UV–vis spectrophotometer with a 1 cm quartz cuvette.

### 5 Cell Culture and Viability Testing

Human osteosarcoma cell line (U-2 OS) cells were cultured in McCoy’s 5A medium (Gibco) supplemented with 10% (v/v) fetal bovine serum (FBS; Gibco) and maintained at 37 °C and 5% CO_2_ during culturing. The COS-7 cells, BHK21 cells, Cardiomyocyte cells and A2780 cells were kindly provided by AmyJet Scientific (Wuhan, China). We tested the cytotoxicity of Cy3-CA, HZ Mito Red, Cy3-NHS, Cy3-COT-NHS, Cy3-Py, Cy3-COT-Py, Cy3-EP, Cy3-COT-EP and HZ Mito Deep Red using CellTiter-Lumi assay. CellTiter-Lumi assay: U-2 OS cells were seeded into 96-well plates and incubated for 24 h at 37 °C with 5% CO_2_. Following incubation, the cells were first rinsed with PBS, and then stained with various concentrations (1 ∼ 20 μM) of Cy3-CA, HZ Mito Red, Cy3-NHS, Cy3-COT-NHS, Cy3-Py, Cy3-COT-Py, Cy3-EP, Cy3-COT-EP, or HZ Mito Deep Red, respectively, for 30 min at 37 °C. After staining, the cells were washed twice with FBS-free culture medium. Subsequently, add 100 μL of the commercial reagent CellTiter-Lumi to each well. This reagent assesses cell viability by detecting ATP generated by metabolically active cells. Chemiluminescence was then measured using a multimode microplate reader (Victor X4). Cell viability was calculated using the following formula:

Cell viability (%) = (Luminescence value of test well/Average luminescence value of control well) × 100.

### 6 Probe Concentration Screening

Incubate U-2 OS cells with different concentrations (0.5, 1, 2.5, 5, 10 μM) of Cy3-CA, HZ Mito Red, Cy3-NHS, Cy3-COT-NHS, Cy3-Py, Cy3-COT-Py, Cy3-EP, and Cy3-COT-EP for 30 min, then perform imaging using a confocal microscope. The imaging parameters for the confocal microscope are set to 10× magnification, a laser power of 15 mW, and an exposure time of 100 ms.

### 7 Mitochondrial Colocalization Assay

Live Cells: U-2 OS cells were first incubated with 10 μM Cy3-CA, 10 μM HZ Mito Red, 10 μM Cy3-NHS, 10 μM Cy3-COT-NHS, 10 μM Cy3-Py, 10 μM Cy3-COT-Py, 2.5 μM Cy3-EP, and 2.5 μM Cy3-COT-EP for 30 min. After incubation, the cells were washed three times with PBS buffer (pH 7.2) and then stained with 0.5 μM MTDR for 10 min, followed by SIM imaging. For the live cell colocalization experiment with 1 μM HZ Mito Deep Red, the same procedure was followed, except MTDR was replaced with MTG.

Fixed Cells: U-2 OS cells transfected with the Cox4-EGFP plasmid were incubated with 10 μM Cy3-CA, 10 μM HZ Mito Red, 10 μM Cy3-NHS, 10 μM Cy3-COT-NHS, 10 μM Cy3-Py, 10 μM Cy3-COT-Py, 2.5 μM Cy3-EP, and 2.5 μM Cy3-COT-EP for 30 min. Subsequently, the cells were fixed with 4% GAfor 10 min, washed three times with PBS, and imaged using SIM. For the fixed cell colocalization experiment with 1 μM HZ Mito Deep Red, the same procedure was used. The PCC was obtained using the colocalization function of ImageJ software.

### 8 Long-Term Dynamic SIM Super-Resolution Imaging of Mitochondria

The cells were first incubated with 0.5 μM MTR, 0.5 μM PK Mito Red, 10 μM Cy3-CA, or 10 μM HZ Mito Red for 30 min. After washing three times with PBS, the cells were imaged using SIM. The imaging conditions for the His-SIM microscope were set as follows: laser power at 5 mW, exposure time at 20 ms, and continuous acquisition without intervals.

### 9 Long-Term Dynamic SIM Super-Resolution Imaging of Mitochondria during Multiple Types of Cell Death

The cells were first incubated with 10 μM HZ Mito Red for 30 min, followed by treatment with 10 μM Eastin, 10 μM OA or 10 μM CCCP to induce ferroptosis, apoptosis, and autophagy, respectively. Subsequently, the cells were imaged using SIM. The imaging conditions for the His-SIM microscope were set as follows: laser power at 5 mW, exposure time at 20 ms, and an interval time of 1 min.

### 10 HZ Mito Red and HZ Mito Deep Red in Immunofluorescence Staining

The cells were first incubated with 10 μM HZ Mito Red or 1 μM HZ Mito Deep Red for 30 min, then fixed in 4% GA for 10 min. Permeabilization was performed with 0.2% Triton X-100 (10 min), followed by standard immunofluorescence staining and imaging.

### 11 Long-Term Dynamic SIM Super-Resolution Imaging of Mitochondria by HZ Mito Deep Red

The cells were first incubated with 0.5 μM MTDR or 10 μM HZ Mito Deep Red for 30 min. After washing three times with PBS, the cells were imaged using SIM. The imaging conditions for the His-SIM microscope were set as follows: laser power at 5 mW, exposure time at 20 ms, and continuous acquisition without intervals.

### 12 Statistical Analysis

Statistical analyses and P-values were computed using GraphPad Prism 8. The statistical differences between experimental and control groups were assessed analyzed by *t* test analysis. For all data, values of *P* < 0.05 indicated statistical significance. The number of mitochondrial cristae was quantified using ImageJ.

## Supplementary Material



## Notes

Supporting methods and additional data, including chemical structure, NMR and HR-MAS (PDF)
