# Supplementary material for: Illuminating Mitochondrial Dynamics: Ultrahigh Labeling Stability Probe for Long-Term SIM Super-Resolution Imaging of Mitochondria
Source: ACS Cent Sci. 2025 Jul 29;11(9):1700–14. doi: 10.1021/acscentsci.5c00695 (PMC12464782; doi:10.1021/acscentsci.5c00695)
Supplement: Supplementary file 1 [file oc5c00695_si_001.pdf]

**Supporting Information**

# Illuminating Mitochondrial Dynamics: Ultra-High Labeling Stability Probe for Long-Term SIM Super-Resolution Imaging of Mitochondria

Xiangpeng Lin,[1†] Xuelei Pang,[1†] Huang Yue,[1†] Xinxin Duan,[1] Yunfei Wei,[1] Ning Jing,[1] Meng Zhang,[1*] Yu-Hui Zhang[1*]

[1]MOE Key Laboratory for Biomedical Photonics, Advanced Biomedical Imaging Facility-Wuhan National Laboratory for Optoelectronics, Huazhong University of Science and Technology, Wuhan, Hubei 430070, China.

[†]These authors contributed equally

*Corresponding Authors: zhangyh@mail.hust.edu.cn (Yu-Hui Zhang); zhangmeng276@hust.edu.cn (Meng Zhang).

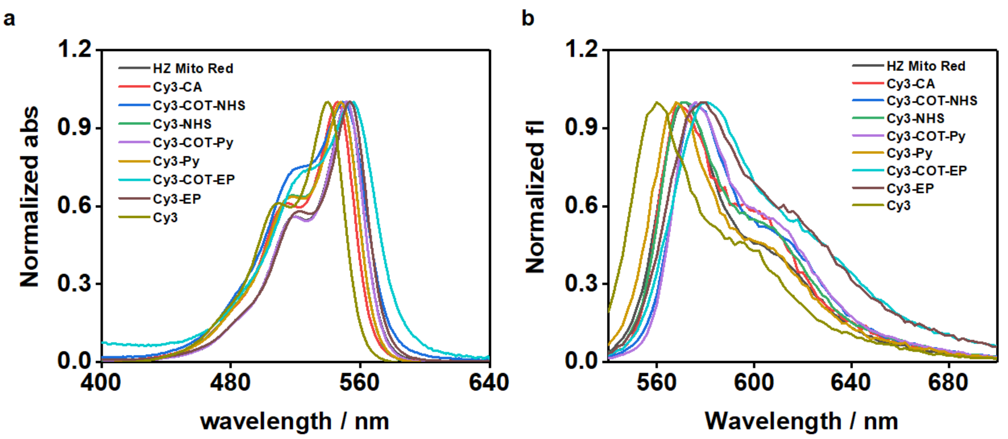


Fig. S1. The absorption (a) and emission (b) spectra of the probes in PBS buffer (pH 7.2).

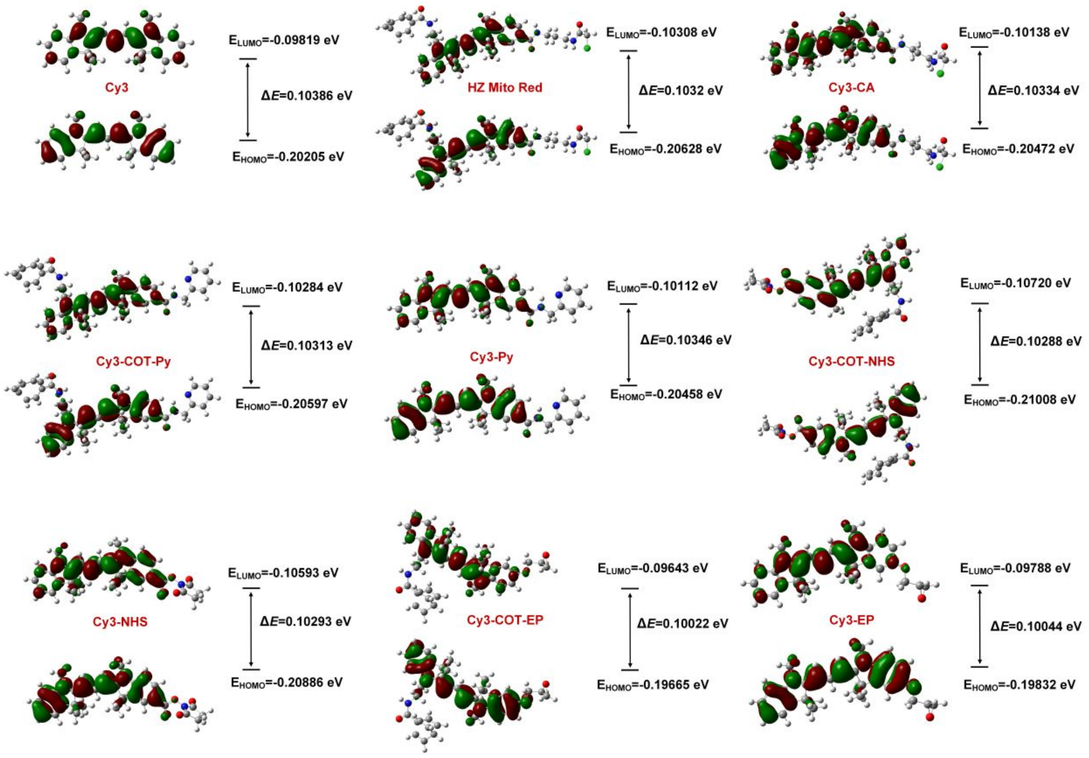


Fig. S2. 3D plot of the HOMO-LUMO energy levels of Cy3 and covalent probes.

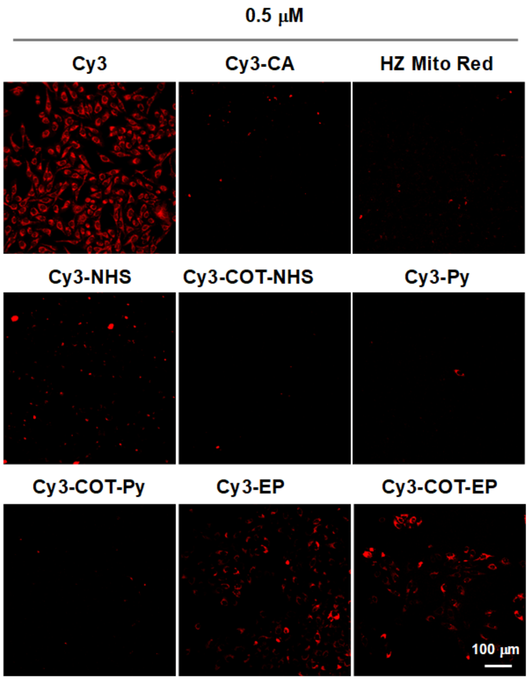


Fig.S3. Confocal imaging of U-2 OS cells stained with 0.5 μM Cy3, 0.5 μM Cy3-CA, 0.5 μM HZ Mito Red, 0.5 μM Cy3-NHS, 0.5 μM Cy3-COT-NHS, 0.5 μM Cy3-Py, 0.5 μM Cy3-COT-Py, 0.5 μM Cy3-EP, and 0.5 μM Cy3-COT-EP, respectively.

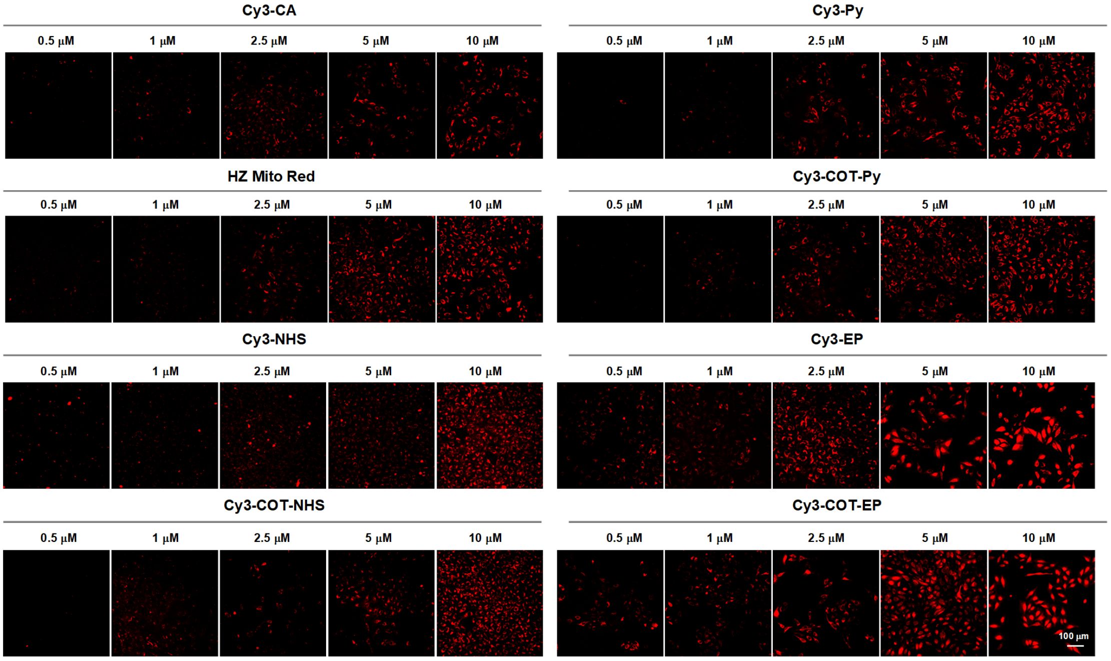


Fig.S4. Confocal imaging of U-2 OS cells separately stained with different concentrations (0.5, 1, 2.5, 5, 10 μM) of Cy3-CA, Cy3-Py, HZ Mito Red, Cy3-COT-Py, Cy3-NHS, Cy3-EP, Cy3-COT-NHS, and Cy3-COT-EP.

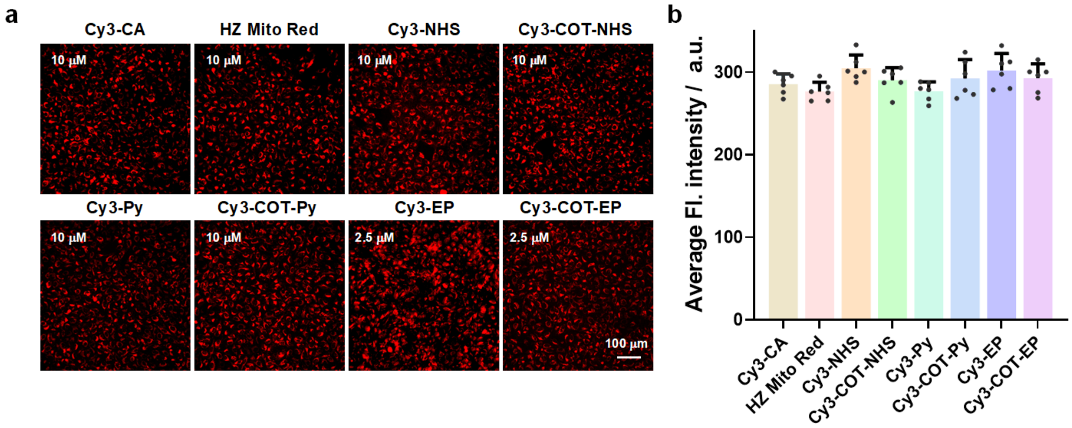


Fig.S5. Confocal images (a) and average fluorescence intensity (b) of U-2 OS cells stained with 10 μM Cy3-CA, 10 μM HZ Mito Red, 10 μM Cy3-NHS, 10 μM Cy3-COT-NHS, 10 μM Cy3-Py, 10 μM Cy3-COT-Py, 2.5 μM Cy3-EP, and 2.5 μM Cy3-COT-EP, respectively. n = 6 independent experiments. All data are presented as mean ± SEM.

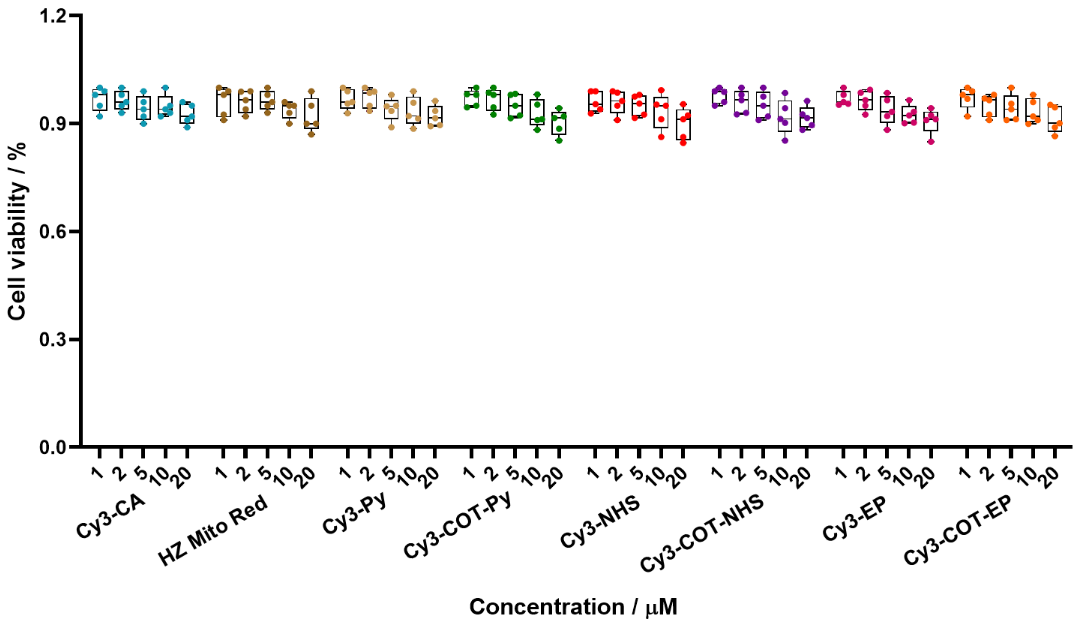


Fig. S6. Cell viability of U-2 OS cells stained with covalent mitochondrial probes at different concentrations (1 ~ 20 μM). n = 5 independent experiments. All data are presented as mean ± SEM.

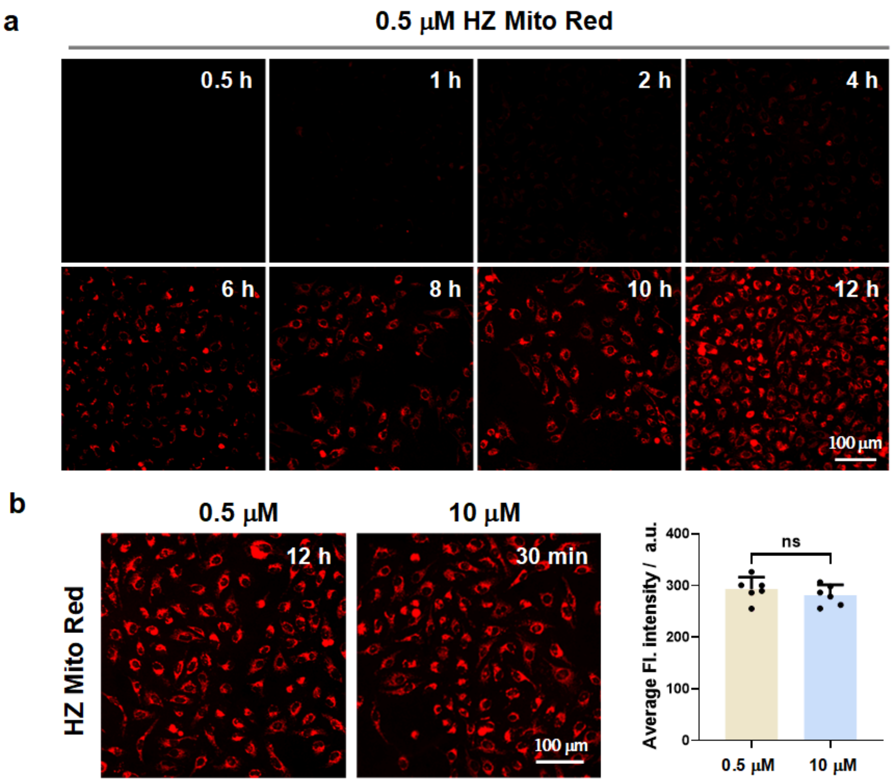


Fig. S7. (a) Confocal images of cells incubated with 0.5 μM HZ Mito Red for different durations. (b) Confocal images and average fluorescence intensity of cells incubated with 0.5 μM HZ Mito Red for 12 h or 30 min. n = 6 independent experiments. All data are presented as mean ± SEM.

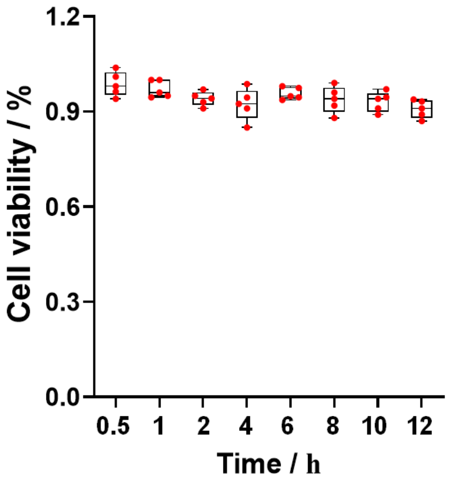


Fig. S8. Cell viability of U-2 OS cells after staining with 0.5 μM HZ Mito Red for different durations. n = 5 independent experiments. All data are presented as mean ± SEM.

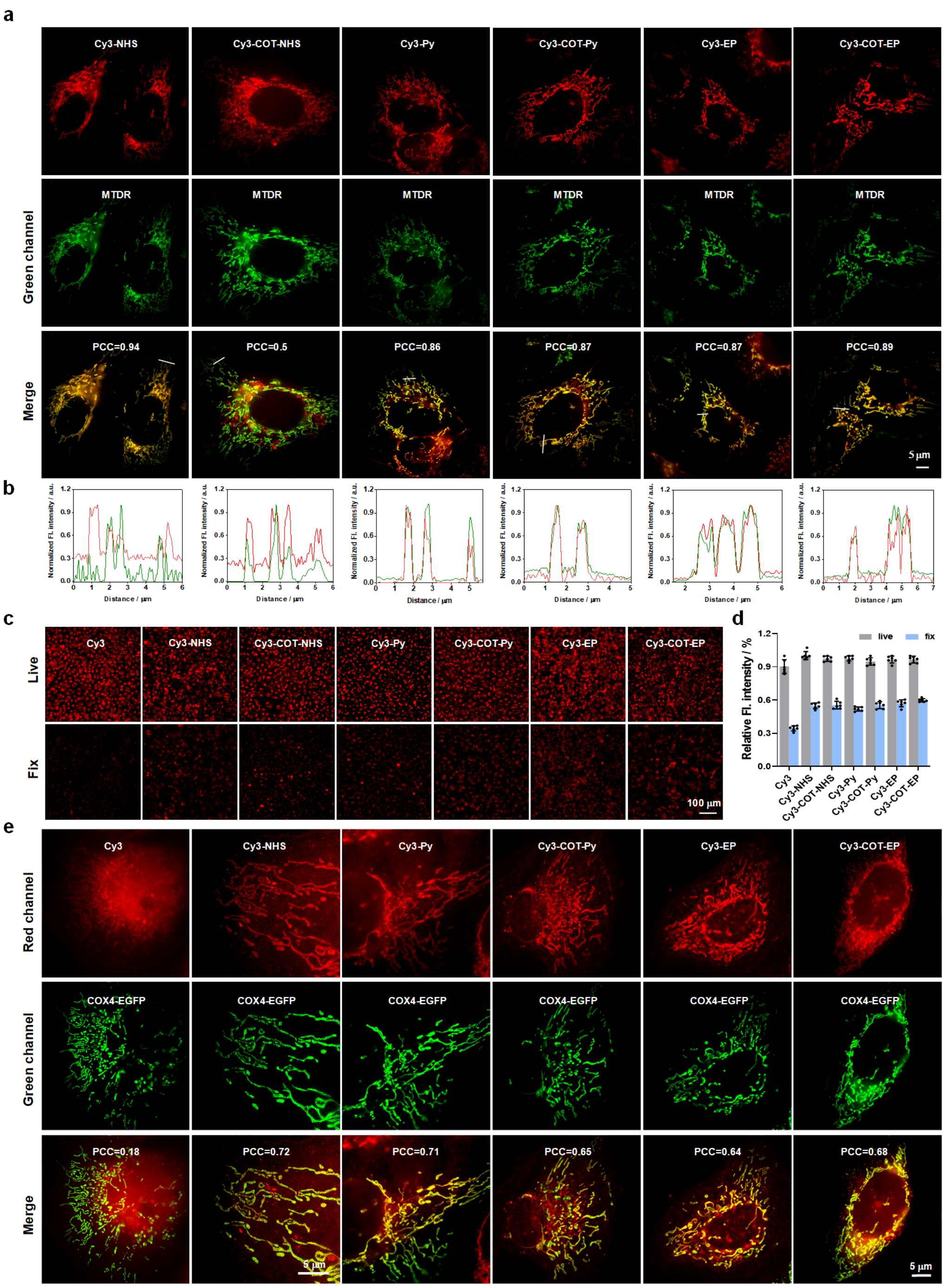


Fig. S9. (a) Co-localization analysis of Cy3-NHS, Cy3-COT-NHS, Cy3-Py, Cy3-COT-Py, Cy3-EP, and Cy3-COT-EP with MTDR in live U-2 OS cells. Cy3-NHS, Cy3-COT-NHS, Cy3-Py, Cy3-COT-Py, Cy3-EP, and Cy3-COT-EP: $\lambda_{ex}$ = 561 nm; $\lambda_{em}$ = 609 nm. MTDR: $\lambda_{ex}$ = 640 nm; $\lambda_{em}$ = 667 nm. (b) Normalized fluorescence intensity distribution profiles along the line in the merged channel. (c) Confocal imaging of cells stained with Cy3, Cy3-NHS, Cy3-COT-NHS, Cy3-Py, Cy3-COT-Py, Cy3-EP, and Cy3-COT-EP, respectively, before and after fixation. (d) Statistical analysis of relative fluorescence intensity before and after fixation (e) Super-resolution images of fixed cells transfected with Cox4-EGFP plasmid, labeled with Cy3, Cy3-NHS, Cy3-COT-NHS, Cy3-Py, Cy3-COT-Py, Cy3-EP, and Cy3-COT-EP, respectively. n = 6 independent experiments. All data are presented as

mean ± SEM.

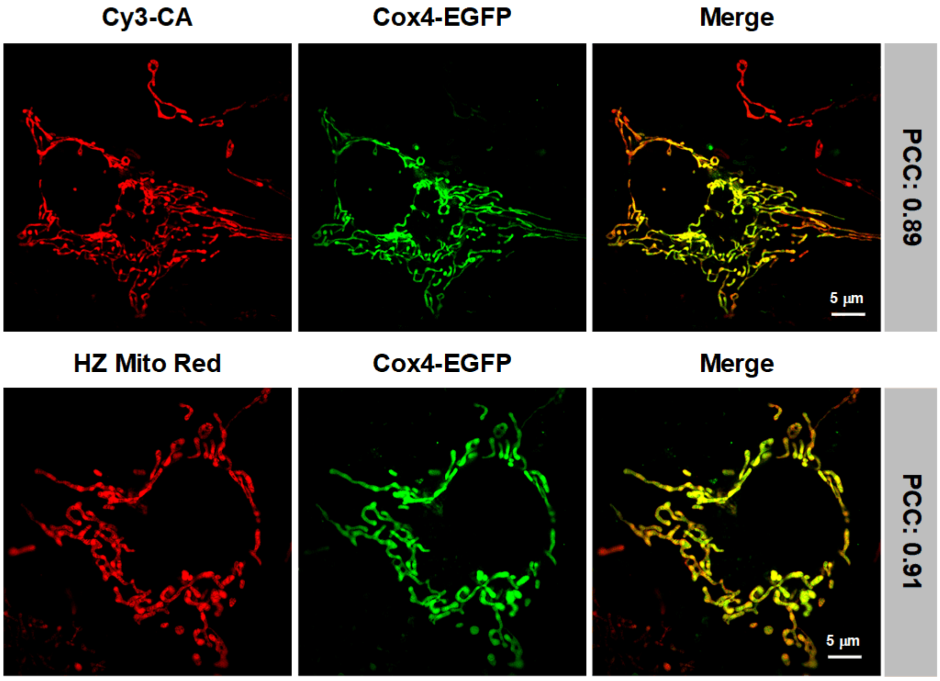


Fig. S10. Super-resolution images of fixed cells transfected with Cox4-EGFP plasmid, labeled with Cy3-CA and HZ Mito Red, respectively.

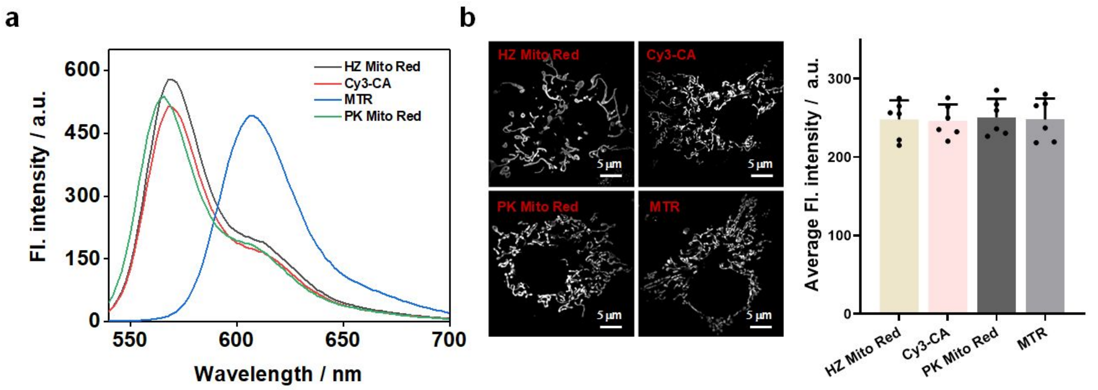


Fig. S11. (a) Fluorescence emission spectra of 0.25 □M HZ Mito Red, 0.25 □M Cy3-CA, 0.25 □M PK Mito Red, and 0.25 □M MTR in PBS solution. (b) Super-resolution imaging and average fluorescence intensity of cells labeled with HZ Mito Red, Cy3-CA, PK Mito Red, and MTR. n = 6 cells from three independent experiments. All data are presented as mean ± SEM.

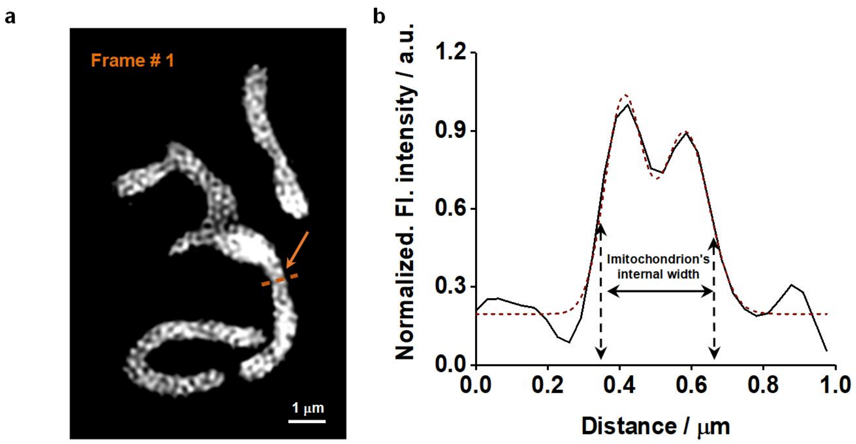


Fig. S12. (a) Super-resolution image of mitochondria in U-2 OS cells labeled with HZ Mito Red. (b) Normalized fluorescence intensity distribution along the line in (a). Fitted with a double Gaussian function, the distance between the peaks at half maximum of the double Gaussian curve represents the mitochondrial width.

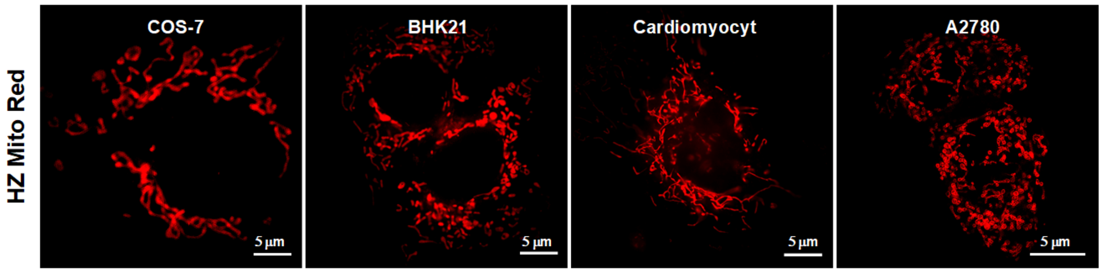


Fig. S13. Super-resolution images of mitochondria in COS-7 cells, BHK21 cells, cardiomyocyte cells, and A2780 cells labeled with HZ Mito Red.

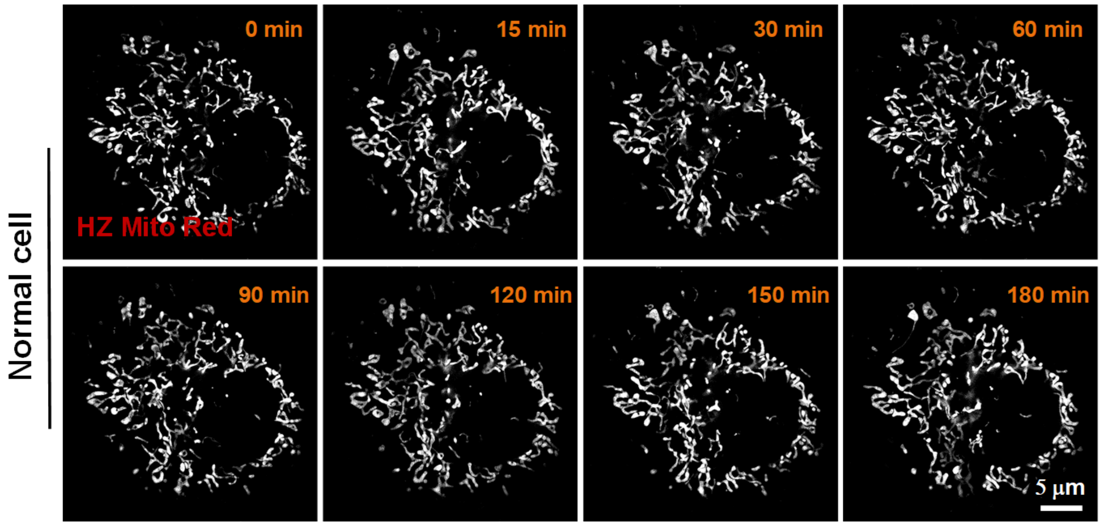


Fig. S14. Long-term dynamic super-resolution imaging of mitochondria in normal cells. Normal cells: Stained solely with 10 µM HZ Mito Red for 30 min. Super-resolution imaging was performed under the same conditions: $\lambda_{ex}$ = 561 nm; $\lambda_{em}$ = 609 nm, with a super-resolution image captured every 1 min.

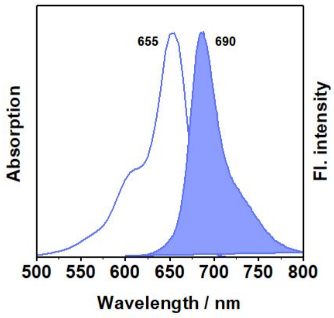


Fig. S15. The absorption and emission spectra of HZ Mito Deep Red.

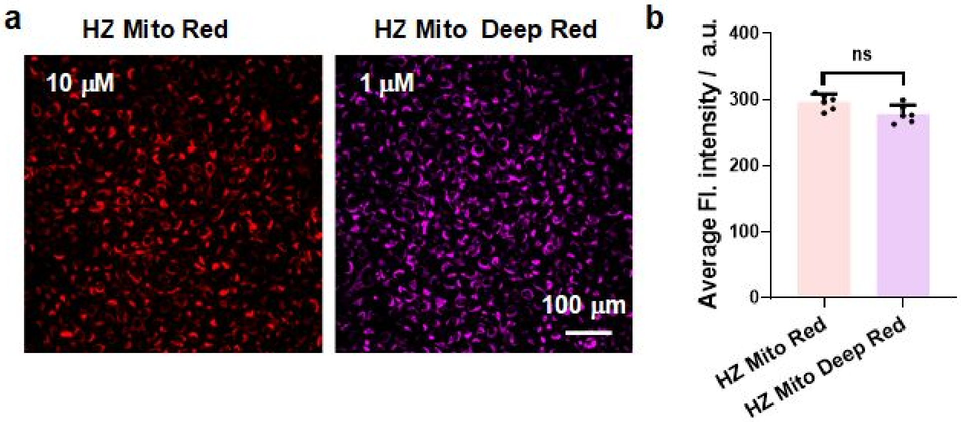


Fig.S16. Confocal images (a) and average fluorescence intensity (b) of U-2 OS cells stained with 10 μM HZ Mito Red and 1 μM HZ Mito Deep Red, respectively. n = 6 independent experiments. All data are presented as mean ± SEM.

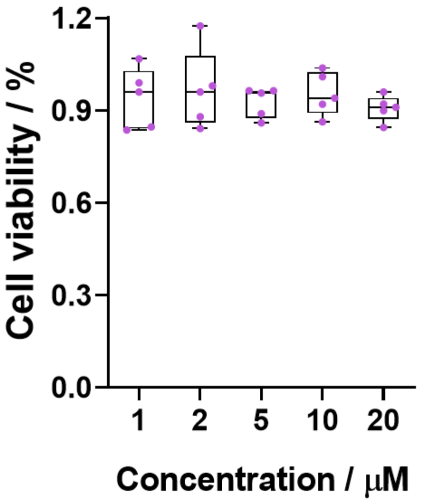


Fig.S17. Cell viability of U-2 OS cells stained with HZ Mito Deep Red at different concentrations (1 ~ 20 μM). n = 5 independent experiments. All data are presented as mean ± SEM.

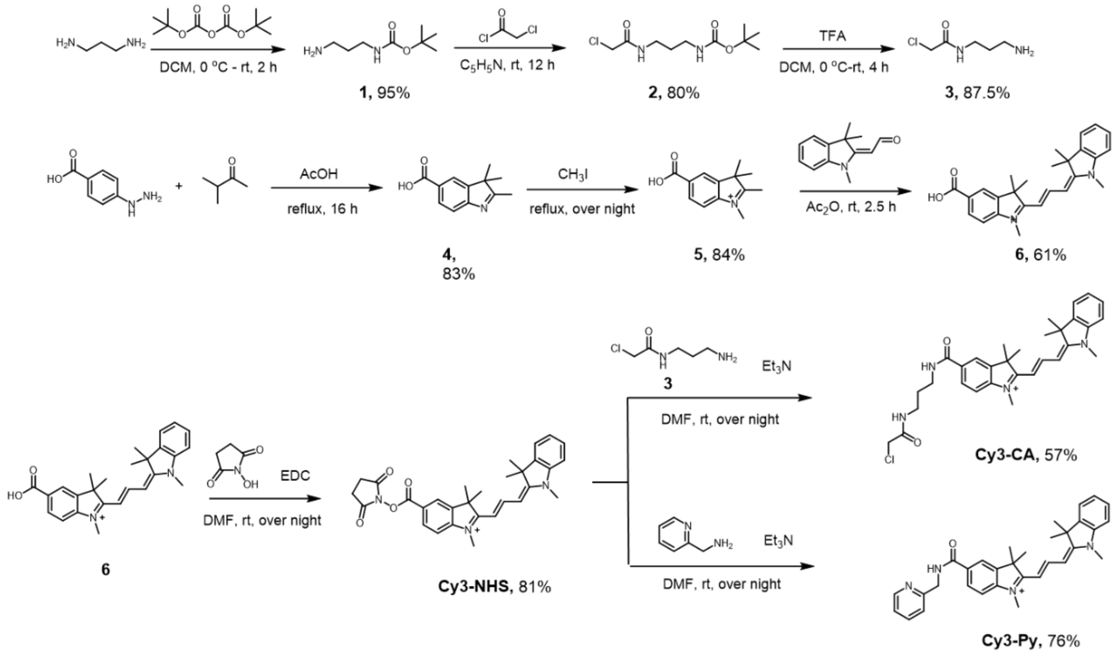


Scheme S1. The synthesis route to Cy3-NHS, Cy3-CA, and Cy3-Py.

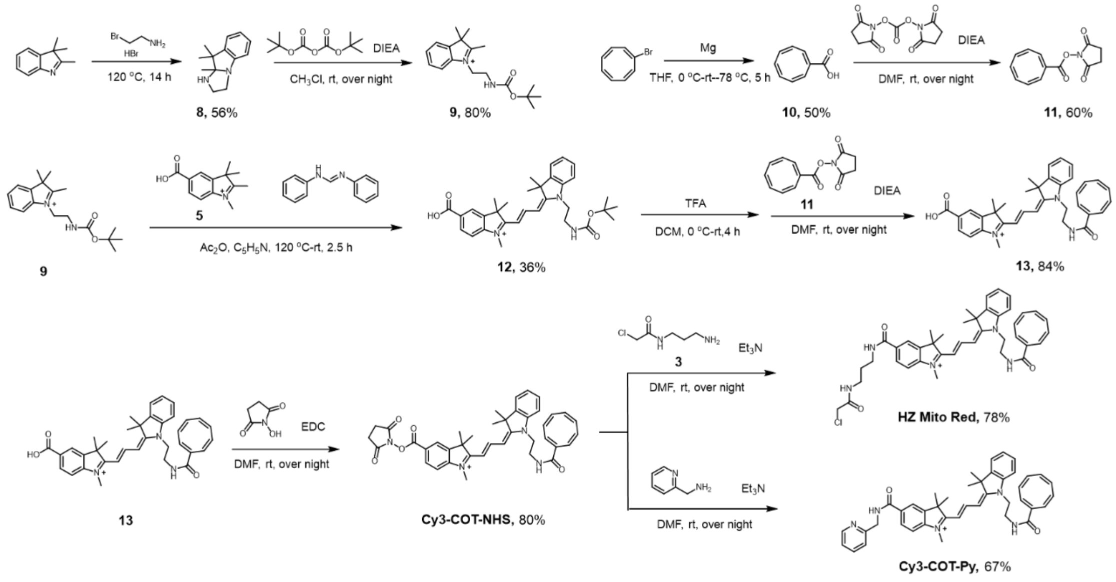


Scheme S2. The synthesis route to Cy3-COT-NHS, HZ Mito Red, and Cy3-COT-Py.

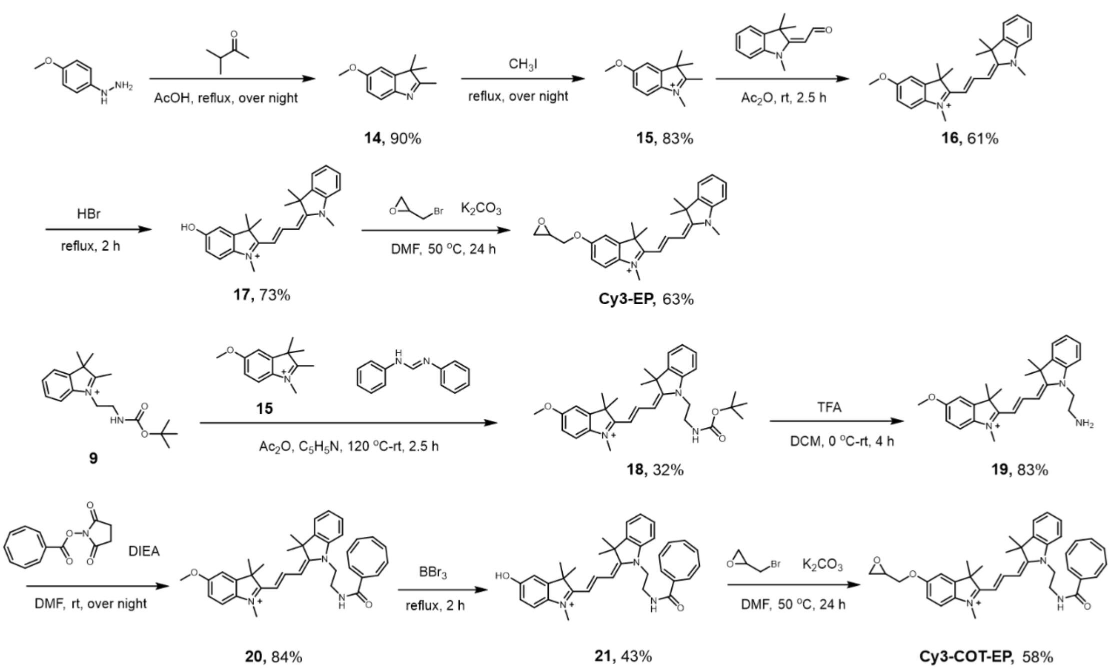

Scheme S3. The synthesis route to Cy3-EP and Cy3-COT-EP.

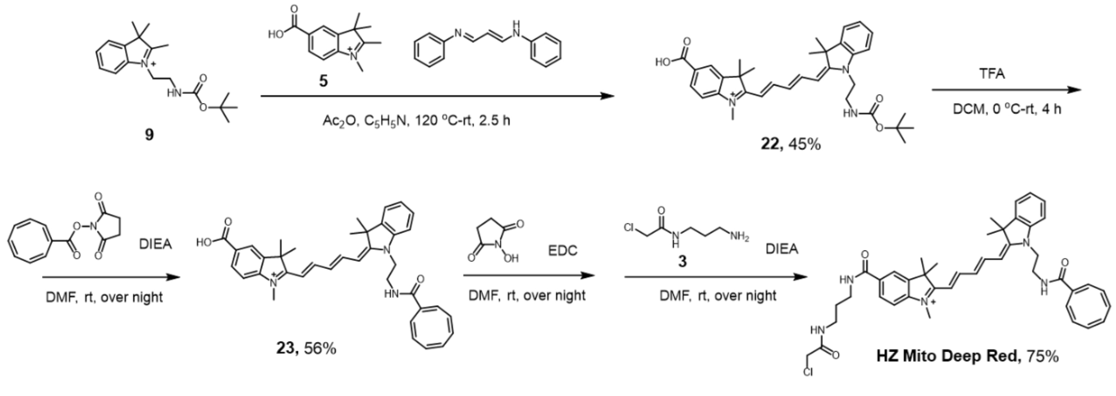

Scheme S4. The synthesis route to HZ Mito Deep Red.

***Compound 1.***

A solution of $Boc_2O$ (1.2 g, 5.5 mmol) in $CHCl_2$ (30 mL) was added dropwise to a solution of diamine (2.03 mL, 27.5 mmol) in $CHCl_2$ (6 mL) over 30 min at 0 °C. The solution was stirred for 2 h at room temperature. The reaction mixture was filtered and concentrated under reduced pressure. Some azeotropes were removed with toluene. The crude residue was dissolved in EtOAc (200 mL) and washed with saline solution (50 mL). Dried with $Na_2SO_4$, filtered and concentrated under reduced pressure to give **1** (957.75 mg, 5.5 mmol, 95%) as a white solid.

$^1$H NMR (400 MHz, $CDCl_3$) δ 4.93 (d, $J$ = 61.1 Hz, 1H), 3.20 (dd, $J$ = 12.5, 6.2 Hz, 2H), 2.75 (t, $J$ = 6.6 Hz, 2H), 1.63 – 1.57 (m, 2H), 1.51 (s, 2H), 1.43 (s, 9H).

The NMR is in accordance with published result, see reference [1].

***Compound 2.***

**1** (800 mg, 4.59 mmol) was dissolved in dry pyridine. The mixture was stirred at 0 °C for 10

min. Chloroacetyl chloride (617.17 mg, 5.51 mmol) was added to the reaction mixture. The reaction mixture was heated to room temperature. Stirring continued for 12 h. The reaction mixture was poured into crushed ice. The mixture was carefully extracted with EtOAc. The residues in the extract were purified by DCM -MeOH gradient column chromatography to obtain **2** (917.9 mg, 3.67 mmol, 80%) as a dark brown solid.

$^1$H NMR (600 MHz, Chloroform-*d*) δ 5.09 – 5.03 (m, 1H), 3.99 (s, 2H), 3.30 (q, *J* = 6.3 Hz, 2H), 3.12 (q, *J* = 6.3 Hz, 2H), 1.62 (p, *J* = 6.2 Hz, 2H), 1.38 (s, 9H).

The NMR is in accordance with published result, see reference [2].

***Compound 3.***

A mixture of 1.5 mL TFA and 2 mL DCM was added dropwise at 0 °C to a round bottom flask containing **2** (70 mg, 0.4 mmol). The reaction solution was then stirred at room temperature for 3 h. At the end of the reaction, the reaction was concentrated under vacuum, the solvent was removed and azeotropised three times with toluene. It was then washed with DCM to obtain **3** (87.17 mg, 0.35 mmol, 87.5%) as a pale yellow oil. LCMS (ESI) calcd for $C_5H_{12}ClN_2O^+$ [$M^+$] 151.0633, found 151.0635.

$^1$H NMR (600 MHz, Methanol-$d_4$) δ 5.48 (d, *J* = 1.5 Hz, 1H), 4.07 (s, 2H), 3.34 (d, *J* = 5.7 Hz, 2H), 2.95 (t, *J* = 7.5 Hz, 2H), 1.88 (t, *J* = 7.2 Hz, 2H).

$^{13}$C NMR (101 MHz, MeOD) δ 168.78, 41.65, 36.85, 36.01, 27.17.

***Compound 4.***

4-Hydrazinobenzoic acid (2 g, 13.15 mmol) and 3-methyl-2-butanone (1.7 g, 19.72 mmol) were refluxed in glacial acetic acid (10 mL) solution for about 16 h. Afterwards, the reaction was cooled down to room temperature and quenched with water. The crude was extracted with dichloromethane and washed with water followed by drying the organic phase with anhydrous $Na_2SO_4$. Dichloromethane was evaporated under vacuum and the crude was purified over a silica gel column chromatography, eluting with MeOH / DCM = 1:10. **4** (2.22 g, 10.92 mmol, 83%) was obtained as a yellowish solid.

$^1$H NMR (600 MHz, Chloroform-d) δ 8.15 (dd, *J* = 8.1, 1.7 Hz, 1H), 8.07 (d, *J* = 1.8 Hz, 1H), 7.68 (d, *J* = 8.1 Hz, 1H), 2.39 (s, 3H), 1.37 (s, 6H).

The NMR is in accordance with published result, see reference [3].

***Compound 5.***

**4** (2 g, 9.84 mmol) was mixed with methyl iodide (8.38 g, 59.08 mmol) in a 50 mL round-bottom flask and refluxed overnight. After the reaction, it is cooled to room temperature, the residue is filtered, and washed with acetonitrile and hexane. **5** (1.8 g, 8.25mmol, 84%) was obtained as a white solid.

$^1$H NMR (400 MHz, MeOD) δ 8.38 (d, *J* = 1.1 Hz, 1H), 8.32 (dd, *J* = 8.4, 1.5 Hz, 1H), 7.97 (d, *J* = 8.4 Hz, 1H), 4.12 (s, 3H), 3.37 (s, 3H), 1.67 (s, 6H).

The NMR is in accordance with published result, see reference [3].

***Compound 6.***

**5** (1 g, 4.58 mmol) and the aldehyde-Fischer base (922.70 mg, 4.58 mmol) were combined together in a solution of glacial acetic anhydride (10 mL). The solution was stirred at room temperature for 2.5 h. To obtain the final product as highly pure, silica gel column chromatography was performed eluting with MeOH / DCM = 20/1 to 15/1. **6** (1.12 g, 2.79 mmol, 61%) was obtained as a dark green solid.

$^{1}$H NMR (600 MHz, Methanol-d4) δ 8.57 (t, $J$ = 13.5 Hz, 1H), 8.12 (dd, $J$ = 5.9, 1.8 Hz, 2H), 7.60 (d, $J$ = 7.4 Hz, 1H), 7.51 – 7.47 (m, 1H), 7.45 (d, $J$ = 7.9 Hz, 1H), 7.37 (dd, $J$ = 7.9, 5.5 Hz, 2H), 6.68 (d, $J$ = 13.7 Hz, 1H), 6.56 (d, $J$ = 13.2 Hz, 1H), 3.79 (s, 3H), 3.71 (s, 3H), 1.79 (s, 12H).

The NMR is in accordance with published result, see reference [3].

***Compound Cy3-NHS.***

**6** (997 mg, 2.49 mmol) and N-hydroxysuccinimide (285.99 mg, 2.49 mmol) were placed in a 100 mL round-bottom flask, EDC (1-(3-dimethylaminopropyl)-3-ethylcarbodiimide) (285.84 mg, 2.49 mmol) was added, followed by 16 mL of DMF. The system was reacted overnight at room temperature. At the end of the reaction, the solvent was removed in vacuum and the pure product **Cy3-NHS** (1001.46 mg, 2.01 mmol, 81%) was obtained as a red solid by extraction with DCM. LCMS (ESI) calcd for $C_{30}H_{32}N_3O_4^+$[M$^+$] 498.2387, found 498.2392.

$^{1}$H NMR (600 MHz, $CDCl_3$) δ 8.44 (t, $J$ = 13.4 Hz, 1H), 8.17 (dt, $J$ = 18.6, 9.3 Hz, 1H), 8.01 (d, $J$ = 1.4 Hz, 1H), 7.57 (d, $J$ = 13.8 Hz, 1H), 7.47 – 7.39 (m, 2H), 7.34 (dd, $J$ = 13.2, 5.4 Hz, 2H), 7.27 (d, $J$ = 5.3 Hz, 1H), 7.16 (d, $J$ = 8.4 Hz, 1H), 3.93 (s, 3H), 3.76 (s, 3H), 2.92 (dd, $J$ = 22.1, 9.5 Hz, 4H), 1.73 (d, $J$ = 5.6 Hz, 12H).

$^{13}$C NMR (151 MHz, $CDCl_3$) δ 176.67, 172.76, 169.51, 161.28, 151.04, 148.29, 142.10, 140.96, 140.45, 132.64, 129.14, 126.78, 123.93, 122.31, 120.31, 112.03, 110.42, 107.51, 104.95, 53.63, 49.75, 47.96, 33.49, 28.26, 27.83, 25.86, 25.58.

***Compound Cy3-CA.***

**Cy3-NHS** (166 mg, 0.33 mmol) and **3** (82.19 mg, 0.33 mmol) was put into a 100 mL round-necked flask and then added $Et_3N$ (33.72 mg, 0.66 mmol) and 2 mL DMF were then added. The reaction was stirred overnight at room temperature. The solution was subsequently concentrated in vacuo. To obtain the final product as highly pure, silica gel column chromatography was performed eluting with methanol/dichloromethane (5% to 8%) to yield **Cy3-CA** (100.30 mg, 0.19 mmol, 57%) as a red solid. LCMS (ESI) calcd for $C_{31}H_{38}ClN_4O_2^+$[M$^+$]: 533.2677, found 533.2671

$^{1}$H NMR (400 MHz, Chloroform-d) δ 8.42 – 8.37 (m, 2H), 8.08 (dd, $J$ = 8.3, 1.7 Hz, 1H), 7.36 (dd, $J$ = 5.6, 1.7 Hz, 2H), 7.27 – 7.22 (m, 1H), 7.15 (d, $J$ = 8.2 Hz, 1H), 7.08 (d, $J$ = 8.3 Hz, 1H), 6.62 (dd, $J$ = 24.3, 13.4 Hz, 2H), 3.68 (s, 3H), 3.62 (s, 3H), 3.48 (d, J = 6.0 Hz, 2H), 3.38 (s, 2H), 3.35 – 3.22 (m, 2H), 1.79 (t, $J$ = 6.1 Hz, 2H), 1.74 (s, 6H), 1.66 (s, 6H).

$^{13}$C NMR (151 MHz, $CDCl_3$) δ 175.35, 175.22, 166.88, 166.64, 166.47, 150.92, 144.53, 142.23, 140.51, 140.35, 129.20, 129.07, 126.19, 122.39, 122.32, 111.16, 110.20, 104.26, 103.83, 49.46, 49.25, 43.01, 36.33, 32.30, 28.89, 28.20, 28.00, 25.54, 22.66.

***Compound Cy3-Py.***

The synthesis route of **Cy3-Py** is similar to **Cy3-CA**, except **Cy3-NHS** (50 mg, 0.1 mmol), 2-Pyridinemethanamine (10.86 mg, 0.1 mmol) and $Et_3N$ (20.32 mg, 0.2 mmol) were used. **Cy3-Py** (37.34 mg, 0.08 mmol, 76 %) was obtained as a magenta product. LCMS (ESI) calcd for $C_{32}H_{35}N_4O^+[M^+]$: 491.2805, found 491.2813

$^1H$ NMR (600 MHz, Chloroform-d) δ 8.52 (d, *J* = 4.9 Hz, 1H), 8.43 (t, *J* = 13.4 Hz, 1H), 8.14 (d, *J* = 1.6 Hz, 2H), 7.97 (dd, *J* = 8.3, 1.7 Hz, 1H), 7.67 (d, *J* = 1.8 Hz, 1H), 7.44 – 7.37 (m, 3H), 7.28 (s, 1H), 7.20 – 7.12 (m, 2H), 4.77 (s, 2H), 3.80 (s, 3H), 3.74 (s, 3H), 3.46 (s, 2H), 1.72 (d, *J* = 18.1 Hz, 12H).

$^{13}C$ NMR (151 MHz, $CDCl_3$) δ 175.91, 174.79, 166.97, 157.18, 151.63, 149.40, 145.77, 142.98, 141.25, 141.13, 137.61, 131.51, 129.58, 128.83, 126.54, 123.01, 122.71, 111.81, 110.67, 106.50, 105.57, 55.80, 53.99, 49.81, 49.25, 45.40, 33.48, 30.24, 28.76, 28.57.

***Compound 8.***

2, 3, 3-trimethylindodine (1.03 g, 6.22 mmol) and 2-bromoethylamine hydrobromide (1.91 g, 9.41 mmol) were added to the pressure-resistant tube. The mixture was stirred at 120 °C in an argon atmosphere for 14 h. After the reaction, the solid was washed with $CHCl_3$, MeOH, and acetone. Subsequently, it was dissolved in DCM and washed with saturated $NaHCO_3$ aqueous solution and saline, and dried with anhydrous $Na_2SO_4$. The crude product was further purified with a silica gel column (eluent: n-hexane / AcOEt = 60/40 to 20/80, then DCM / MeOH = 100/0 to 90/10) to give **8** (0.73 g, 3.51 mmol. 56%) as a dark brown solid.

$^1H$ NMR (600 MHz, $CDCl_3$) δ 7.19 – 7.03 (m, 2H), 6.88 (t, *J* = 7.2 Hz, 1H), 6.67 (d, *J* = 7.7 Hz, 1H), 3.45 (ddd, *J* = 24.9, 12.1, 7.5 Hz, 2H), 3.05 (ddd, *J* = 25.5, 12.0, 7.6 Hz, 2H), 2.12 (s, 1H), 1.41 (s, 3H), 1.32 (d, *J* = 9.3 Hz, 3H), 1.22 (s, 3H).

The NMR is in accordance with published result, see reference [4].

***Compound 9.***

Compound **8** (300 mg, 1.48 mmol), $Boc_2O$ (288 mg, 1.78 mmol), and DIEA (0.39 mL, 2.22 mmol) was added to a 10 mL round-bottomed flask containing $CHCl_3$ (5 mL) and stirred overnight at room temperature under nitrogen. After the reaction, it was diluted with DCM, washed with saturated $NaHCO_3$ aqueous solution and saline, and dried on $Na_2SO_4$. The solvent was evaporated and purified by silica gel column chromatography (eluent: DCM / MeOH = 100/0 to 90/10) to give **9** (362 mg, 1.19 mmol, 80%) as a pink oil.

$^1H$ NMR (600 MHz, $CDCl_3$) δ 7.26 (t, *J* = 7.7 Hz, 1H), 7.22 (d, *J* = 6.8 Hz, 1H), 7.07 (t, *J* = 7.2 Hz, 1H), 7.00 (d, *J* = 7.2 Hz, 1H), 4.92 (s, 1H), 3.87 (s, 2H), 3.42 (d, *J* = 5.0 Hz, 2H), 1.52 – 1.22 (m, 18H).

The NMR is in accordance with published result, see reference [4].

***Compound 10.***

Add magnesium granules (1.0 g, 42 mmol) to a dry 50 mL double-mouth round-bottom flask and purge with argon. Dissolve COTBr (1.0 g, 5.8 mmol) in ultra-dry THF, then, in an ice bath, Ar atmosphere, slowly add it dropwise to a double-mouth round-bottom flask containing magnesium

grains and continue stirring for 1h. The system was then raised to room temperature and stirred for 4 h until a blue-green solution was generated. The resulting solution was cooled to −78 °C and an excess of solid dry ice was added. Quench the reaction with 20 mL of water and acidify to pH = 2 with 1 M HCl. The resulting carboxylic acid was extracted from an aqueous solution using n-hexane and then purified by flash chromatography (EA / PE = 5/1) on a silica gel column to obtain 10 (430 mg, 2.9 mmol, 50%) as a yellow solid.

$^1$H NMR (400 MHz, $CDCl_3$) δ 7.17 (s, 1H), 6.20 – 5.77 (m, 6H).

The NMR is in accordance with published result, see reference [5].

***Compound 11.***

Dissolve **10** (180 mg, 1.22 mmol), DIEA (236.5 mg, 1.83 mmol), and N.N'-Disuccinimidyl Carbonate (625.05 mg, 2.44 mmol) in a round-bottom flask containing 5 mL of dried DMF. The reaction solution is stirred overnight at room temperature. After the end of the reaction, the resulting mixture was purified with silica gel (eluent: EtOAc : hexane = 1:3) to obtain **11** (187 mg, 0.73 mmol, 60%), which is a light yellow oil.

$^1$H NMR (400 MHz, $CDCl_3$) δ 7.27 (d, $J$ = 7.5 Hz, 1H), 6.19 – 5.64 (m, 6H), 2.83 (s, 4H).

The NMR is in accordance with published result, see reference [6].

***Compound 12.***

**5** (120 mg, 0.56 mmol) and N,N'-diphenylformamidine (129.2 mg, 0.68 mmol) were dissolved in a 50 mL round-bottom flask containing 5 mL of acetic anhydride and heated to 120 °C for 30 min. Subsequently, the reaction mixture is cooled to room temperature. Add **9** (206 mg, 0.68 mmol) of dry pyridine (10 mL) solution to the mixture. Stir the mixture at room temperature for 2 h. Vacuum to remove the solvent. Dissolve the residual oil in chloroform (50 mL). Wash sequentially with clean water and 0.1 M hydrochloric acid. Dry the organic layer with $Na_2SO_4$. The crude product was purified by column chromatography (eluent: DCM / MeOH = 50/1 to 20/1) to obtain **12** (106 mg, 0.2 mmol, 36%) as a red solid. LCMS (ESI) calcd for $C_{32}H_{40}N_3O_4^+$ [$M^+$] 530.30133, found 530.30072.

$^1$H NMR (600 MHz, MeOD) δ 8.61 (t, $J$ = 13.4 Hz, 1H), 8.15 (d, $J$ = 3.9 Hz, 2H), 7.58 (d, $J$ = 7.4 Hz, 1H), 7.48 (d, $J$ = 3.3 Hz, 2H), 7.38 (dd, $J$ = 13.1, 5.9 Hz, 2H), 6.64 (d, $J$ = 13.6 Hz, 1H), 6.45 (d, $J$ = 13.2 Hz, 1H), 4.34 (t, $J$ = 5.3 Hz, 2H), 3.69 (s, 3H), 3.63 – 3.54 (m, 2H), 1.82 (d, $J$ = 5.8 Hz, 12H), 1.24 (d, $J$ = 8.9 Hz, 9H).

$^{13}$C NMR (151 MHz, MeOD) δ 176.67, 174.81, 156.84, 151.21, 142.43, 141.21, 140.36, 130.88, 129.47, 129.39, 128.45, 125.89, 123.16, 122.07, 111.84, 110.04, 104.15, 102.50, 78.79, 49.76, 48.59, 44.67, 37.82, 27.20, 26.89, 26.56, 22.34.

***Compound 13.***

Add **12** (100 mg, 0.19 mmol) to a round-bottom flask containing 3 mL of DCM. In an ice bath, add TFA (2 mL) dropwise. Subsequently, transfer the reaction mixture to room temperature and continue stirring for 4 h. The product was obtained after the solvent was removed by vacuum. No further purification is required and is used directly for the next step. The product was dissolved in

3 mL DMF, then **11** (46.6 mg, 0.19 mmol) and DIEA (49 mg, 0.38 mmol) were added, and the reaction mixture was stirred overnight in an Ar atmosphere. After the reaction is over. The solvent was removed by vacuum, and the crude product was purified by silica gel column chromatography (eluent: DCM / MeOH = 50/1 to 10/1) to obtain a product of **13** (89.6 mg, 0.16 mmol, 84%), which was a red solid. LCMS (ESI) calcd for $C_{36}H_{38}N_3O_3^+$ [M$^+$] 560.2908, found 560.2897.

$^1$H NMR (600 MHz, MeOD) δ 8.58 (t, *J* = 13.5 Hz, 1H), 8.15 (t, *J* = 3.4 Hz, 2H), 7.59 (d, *J* = 7.4 Hz, 1H), 7.51 – 7.43 (m, 2H), 7.39 (dd, *J* = 14.8, 7.9 Hz, 2H), 7.19 (dd, *J* = 32.6, 7.5 Hz, 2H), 6.70 – 6.56 (m, 2H), 6.39 (d, *J* = 13.2 Hz, 1H), 5.81 (ddd, *J* = 58.8, 49.6, 11.2 Hz, 6H), 4.42 (s, 2H), 3.77 (s, 2H), 3.69 (s, 3H), 1.81 (d, *J* = 4.3 Hz, 12H).

$^{13}$C NMR (151 MHz, MeOD) δ 175.61, 166.25, 149.61, 145.03, 140.38, 139.63, 138.93, 136.49, 134.63, 133.22, 131.45, 129.95, 129.49, 128.26, 127.12, 126.98, 126.27, 124.51, 123.36, 121.68, 120.63, 110.09, 108.64, 102.84, 101.06, 48.32, 47.05, 41.83, 35.43, 25.37, 25.09.

***Compound Cy3-COT-NHS.***

The synthesis route of **Cy3-COT-NHS** is similar to **Cy3-NHS**, except **13** (85 mg, 0.15 mmol) were used. **Cy3-COT-NHS** (79 mg, 0.12 mmol, 80%) was obtained as a red solid. LCMS (ESI) calcd for $C_{40}H_{41}N_4O_5^+$[M$^+$] 657.3071, found 657.3055.

$^1$H NMR (600 MHz, $CDCl_3$) δ 8.66 (s, 1H), 8.42 (d, *J* = 11.1 Hz, 1H), 8.20 (d, *J* = 8.2 Hz, 1H), 7.94 (d, *J* = 12.0 Hz, 1H), 7.57 – 7.47 (m, 2H), 7.45 – 7.33 (m, 2H), 7.22 (d, *J* = 12.0 Hz, 1H), 7.13 (d, *J* = 8.4 Hz, 1H), 6.92 (s, 1H), 6.29 – 5.66 (m, 6H), 5.35 (d, *J* = 3.1 Hz, 1H), 4.54 (s, 2H), 3.89 (s, 2H), 3.69 (s, 3H), 2.89 – 2.73 (m, 4H), 1.79 – 1.60 (m, 12H).

$^{13}$C NMR (151 MHz, $CDCl_3$) δ 177.20, 171.70, 169.34, 166.88, 162.53, 161.30, 150.83, 148.48, 141.58, 141.07, 140.30, 136.40, 132.77, 131.33, 129.38, 126.83, 124.05, 122.12, 120.08, 112.39, 109.77, 109.22, 105.30, 49.85, 47.72, 44.75, 36.94, 36.49, 29.69, 28.41, 27.65, 25.72.

***Compound HZ Mito Red.***

The synthesis route of **HZ Mito Red** is similar to **Cy3-CA**, except ***Cy3-COT-NHS*** (15 mg, 0.023 mmol) were used. **HZ Mito Red** (12.5 mg, 0.018 mmol, 78%) was obtained as a red solid. LCMS (ESI) calcd for $C_{41}H_{47}ClN_5O_5^+$[M$^+$] 692.3362, found 692.3348.

$^1$H NMR (600 MHz, MeOD) δ 8.56 (s, 1H), 8.03 – 7.92 (m, 2H), 7.56 (d, *J* = 7.4 Hz, 1H), 7.44 (dd, *J* = 19.0, 7.6 Hz, 4H), 7.37 (dd, *J* = 18.4, 7.9 Hz, 2H), 6.62 (s, 2H), 6.55 (d, *J* = 13.6 Hz, 1H), 6.37 (d, *J* = 13.2 Hz, 1H), 6.14 – 5.52 (m, 6H), 5.34 (d, *J* = 4.8 Hz, 1H), 4.39 (s, 2H), 4.07 (s, 2H), 3.74 (d, *J* = 5.6 Hz, 2H), 3.67 (s, 3H), 3.46 (t, *J* = 6.7 Hz, 2H), 3.34 (t, *J* = 6.7 Hz, 2H), 1.89 – 1.76 (m, 14H).

$^{13}$C NMR (151 MHz, MeOD) δ 176.86, 174.79, 168.11, 167.89, 167.78, 151.05, 145.43, 141.95, 141.09, 138.00, 136.19, 134.76, 131.49, 130.90, 129.47, 129.39, 128.64, 128.31, 125.91, 122.16, 121.13, 111.51, 110.29, 104.05, 102.58, 49.77, 48.80, 43.27, 41.82, 36.83, 35.14, 31.66, 26.90, 26.68, 25.52, 22.33.

***Compound Cy3-COT-Py.***

The synthesis route of **Cy3-COT-Py** is similar to **Cy3-Py**, except ***Cy3-COT-NHS*** (20 mg, 0.03

mmol) were used. **HZ Mito Red** (13 mg, 0.02 mmol, 67%) was obtained as a red solid. LCMS (ESI) calcd for $C_{42}H_{44}N_5O_2^+$[$M^+$] 650.34895, found 650.34930.

$^1$H NMR (600 MHz, $CDCl_3$) δ 8.60 – 8.48 (m, 2H), 8.07 (d, *J* = 1.4 Hz, 1H), 8.02 (dd, *J* = 8.3, 1.6 Hz, 1H), 7.83 (d, *J* = 1.6 Hz, 1H), 7.56 (d, *J* = 7.3 Hz, 1H), 7.50 – 7.39 (m, 4H), 7.35 (dd, *J* = 10.3, 5.6 Hz, 2H), 6.69 – 6.48 (m, 2H), 6.38 (d, *J* = 13.2 Hz, 1H), 6.05 – 5.59 (m, 6H), 5.34 (t, *J* = 4.7 Hz, 1H), 4.72 (s, 2H), 4.40 (s, 2H), 3.74 (s, 2H), 3.68 (s, 3H), 1.79 (d, *J* = 11.1 Hz, 12H).

$^{13}$C NMR (151 MHz, $CDCl_3$) δ 180.86, 178.69, 171.83, 171.71, 161.90, 149.56, 145.88, 145.05, 141.95, 141.48, 140.12, 138.71, 136.93, 135.43, 134.46, 133.41, 133.33, 132.58, 132.47, 131.89, 129.88, 126.40, 126.10, 125.61, 125.25, 115.49, 114.26, 108.08, 106.54, 53.73, 52.72, 48.60, 40.89, 39.07, 35.60, 29.46, 26.27.

***Compound 14.***

4-methoxy-phenylhydrazine hydrochloride (1 g, 7.28 mmol) and isopropyl methyl ketone (0.624 g, 7.28 mmol) were added to glacial acetic acid (10 mL). The mixture was refluxed for 10 h with stirring. Then the mixture was cooled and neutralized with 1 M $Na_2CO_3$ then diluted with water (100 mL) and extracted with $CHCl_2$. The organic layer is dried on $Na_2SO_4$ and the solvent is removed by evaporation. **14** (1.24 g, 6.6 mmol, 90%) was prepared as a red viscous oil in high yield.

$^1$H NMR (600 MHz, Chloroform-d) δ 7.44 (d, *J* = 8.3 Hz, 1H), 6.85 – 6.80 (m, 2H), 3.82 (s, 3H), 2.25 (s, 3H), 1.28 (s, 6H).

The NMR is in accordance with published result, see reference [7].

***Compound 15.***

**14** (1 g, 5.28 mmol) and methyl iodide (4.5 g, 31.73 mmol) were dissolved in acetonitrile (20 mL), refluxed and stirred overnight in an argon atmosphere. The reactants were cooled to room temperature and washed 3 times with ethyl acetate to give **15** as a brown solid (895 mg, 4.38 mmol, 83%). It can be used directly for the next reaction without further purification.

$^1$H NMR (400 MHz, DMSO-d6) δ 7.82 (d, *J* = 8.8 Hz, 1H), 7.48 (d, *J* = 2.5 Hz, 1H), 7.14 (dd, *J* = 8.8, 2.5 Hz, 1H), 3.95 (s, 3H), 3.86 (s, 3H), 2.73 (s, 3H), 1.52 (s, 6H).

The NMR is in accordance with published result, see reference [8].

***Compound 16.***

**15** (500 mg, 2.45 mmol) and the aldehyde-Fischer base (493.11 mg, 2.45 mmol) were combined together in a solution of glacial acetic anhydride (2 mL). The solution was stirred at room temperature for 2.5 h. Then the solvent was removed by means of evaporation. To obtain the final product as highly pure, silica gel column chromatography was performed eluting with dichloromethane/methanol 5% to 8%. **16** (579.19 mg, 1.5 mmol, 61%) was obtained as a red solid.

$^1$H NMR (400 MHz, Chloroform-d) δ 8.32 (t, *J* = 13.4 Hz, 1H), 7.33 (d, *J* = 7.6 Hz, 2H), 7.19 (d, *J* = 7.4 Hz, 1H), 7.08 (t, *J* = 8.0 Hz, 2H), 7.02 (d, *J* = 13.6 Hz, 1H), 6.95 (d, *J* = 2.5 Hz, 2H), 6.90–6.86 (m, 1H), 3.83 (s, 3H), 3.75 (s, 3H), 3.68 (s, 3H), 1.67 (d, *J* = 5.7 Hz, 12H).

The NMR is in accordance with published result, see reference [9].

***Compound 17.***

**16** (500 mg 1.29 mmol) and hydrobromic acid (2 mL) were combined together in a 25 mL sealed reaction vial. The mixture was refluxed for 2 h with stirring. Then the mixture was cooled neutralized with $NaHCO_3$ then diluted with water (100 mL) and extracted with $CHCl_2$. Following the drying of the organic layer over $MgSO_4$, the solvent was removed by means of evaporation. The crude product is purified by column chromatography (eluent; DCM / MeOH = 20/1 to 15:1) to obtain a high purity of **17** (351.74 mg, 0.94 mmol, 73%) as a red solid.

$^1$H NMR (600 MHz, Chloroform-d) δ 8.32 (t, *J* = 13.5 Hz, 1H), 7.41 – 7.35 (m, 2H), 7.29 – 7.27 (m, 1H), 7.22 (t, *J* = 7.5 Hz, 1H), 7.16 – 7.07 (m, 2H), 6.98 (d, *J* = 8.5 Hz, 1H), 6.76 (d, *J* = 13.6 Hz, 1H), 6.65 (d, J = 13.1 Hz, 1H), 3.71 (s, 3H), 3.65 (s, 3H), 1.69 (d, *J* = 14.2 Hz, 12H).

***Compound Cy3-EP.***

Compound **17** (50 mg, 0.13 mmol), $K_2CO_3$ (36.76 mg, 0.27 mmol) and DMF (2 mL) were combined together in a solution of glacial acetic anhydride (5 mL). Next, epoxybromopropane (91.09 mg, 0.67 mmol) was added dropwise to the system, heated to 50 °C and stir the mixture for 24 h. Then the mixture was cooled and then diluted with water (100 mL) and extracted with $CHCl_2$. Following the drying of the organic layer over $MgSO_4$, the solvent was removed by means of evaporation. The crude product is purified by column chromatography (eluent; DCM / MeOH = 20/1 to 15:1) to obtain a high purity of **Cy3-EP** (36 mg, 0.08 mmol, 63%) as a red solid. LCMS (ESI) calcd for $C_{28}H_{33}N_2O_2^+$[M$^+$] 429.25365, found 429.25446

$^1$H NMR (600 MHz, Chloroform-d) δ 8.39 (td, *J* = 13.4, 1.9 Hz, 1H), 7.44 – 7.35 (m, 3H), 7.27 – 7.23 (m, 1H), 7.21 – 7.17 (m, 1H), 7.14 – 7.08 (m, 3H), 6.99 – 6.94 (m, 1H), 3.81 (d, *J* = 8.0 Hz, 3H), 3.77 (d, *J* = 5.6 Hz, 3H), 2.94 (d, J = 44.5 Hz, 1H), 2.35 – 2.22 (m, 2H), 2.03 (d, *J* = 6.4 Hz, 2H), 1.73 (d, *J* = 8.9 Hz, 12H).

$^{13}$C NMR (101 MHz, $CDCl_3$) δ 174.35, 173.30, 157.73, 149.70, 142.83, 142.41, 140.23, 136.21, 129.86, 128.80, 124.95, 122.00, 114.70, 111.74, 110.36, 109.92, 104.85, 103.52, 69.57, 69.35, 53.38, 49.47, 48.70, 46.01, 35.89, 25.54, 22.65.

***Compound 18.***

The synthesis route of **18** is similar to **12**, except **15** (200 mg, 0.98 mmol) were used. **18** (160.06 mg, 0.31 mmol, 32%) was obtained as a red solid. LCMS (ESI) calcd for $C_{32}H_{42}N_3O_3^+$[M$^+$] 516.3212, found 516.3221.

$^1$H NMR (600 MHz, MeOD) δ 8.51 (dt, *J* = 38.4, 13.5 Hz, 1H), 7.53 (dd, *J* = 17.8, 7.4 Hz, 1H), 7.44 (dd, *J* = 15.7, 7.8 Hz, 1H), 7.33 (ddd, *J* = 26.9, 10.7, 5.4 Hz, 1H), 7.21 (dd, *J* = 18.4, 1.9 Hz, 1H), 7.05 – 7.01 (m, 1H), 6.50 (t, *J* = 12.5 Hz, 1H), 6.37 (d, *J* = 13.5 Hz, 1H), 4.25 (t, *J* = 5.4 Hz, 1H), 3.90 (d, *J* = 6.8 Hz, 4H), 3.70 (d, *J* = 32.1 Hz, 4H), 3.59 – 3.54 (m, 1H), 3.35 (s, 3H), 1.81 (dd, *J* = 14.6, 11.2 Hz, 12H), 1.26 (d, *J* = 12.5 Hz, 6H).

$^{13}$C NMR (151 MHz, MeOD) δ 173.91, 159.03, 158.65, 156.86, 149.82, 148.91, 142.79, 142.28, 140.56, 136.18, 128.25, 124.67, 121.90, 113.43, 111.92, 111.40, 110.79, 108.57, 102.77, 101.61, 78.75, 55.07, 49.25, 43.91, 37.58, 30.79, 27.23, 26.83.

***Compound 19.***

Add **18** (150 mg, 0.29 mmol) to a round-bottom flask containing 3 mL of DCM. In an ice bath, add TFA (2 mL) dropwise. Subsequently, transfer the reaction mixture to room temperature and continue stirring for 4 h. The **19** (123 mg, 0.24 mmol, 83%) was obtained as a red solid after the solvent was removed by vacuum.

$^{1}$H NMR (600 MHz, MeOD) δ 8.53 (t, *J* = 13.4 Hz, 1H), 7.53 (d, *J* = 7.3 Hz, 1H), 7.47 – 7.38 (m, 2H), 7.34 – 7.26 (m, 2H), 7.23 (d, *J* = 2.3 Hz, 1H), 7.06 (dd, *J* = 8.7, 2.4 Hz, 1H), 6.50 (d, *J* = 13.9 Hz, 1H), 6.43 (d, *J* = 12.9 Hz, 1H), 4.41 (t, *J* = 7.3 Hz, 2H), 3.90 (s, 3H), 3.77 (s, 3H), 3.42 – 3.35 (m, 4H), 1.81 (t, *J* = 11.5 Hz, 12H).

$^{13}$C NMR (151 MHz, MeOD) δ 176.03, 172.66, 159.59, 149.84, 143.04, 141.89, 140.05, 135.75, 128.56, 124.62, 122.25, 113.84, 112.65, 109.45, 108.43, 104.34, 100.72, 55.09, 50.02, 48.67, 40.43, 35.86, 31.04, 27.24, 26.45.

***Compound 20.***

The synthesis route of **20** is similar to **13**, except **19** (100 mg, 0.19 mmol) were used. **20** (87.4 mg, 0.16 mmol, 84%) was obtained as a red solid. LCMS (ESI) calcd for $C_{36}H_{40}N_3O_2^+$[M$^+$] 546.3115, found 546.3130.

$^{1}$H NMR (600 MHz, MeOD) δ 8.50 (s, 1H), 7.52 (d, *J* = 7.4 Hz, 1H), 7.42 (d, *J* = 7.6 Hz, 1H), 7.33 (dd, *J* = 8.2, 4.5 Hz, 2H), 7.29 (d, *J* = 7.4 Hz, 1H), 7.20 (d, *J* = 2.2 Hz, 1H), 7.04 (dd, *J* = 8.7, 2.3 Hz, 1H), 6.65 (s, 1H), 6.39 (dd, *J* = 17.4, 13.5 Hz, 2H), 6.03 – 5.66 (m, 6H), 4.31 (s, 2H), 3.89 (s, 3H), 3.77 – 3.64 (m, 5H), 1.78 (d, *J* = 3.1 Hz, 12H).

$^{13}$C NMR (151 MHz, MeOD) δ 174.84, 174.23, 167.79, 159.10, 149.69, 142.57, 142.29, 140.47, 137.90, 136.28, 135.94, 132.97, 131.47, 128.44, 128.01, 124.77, 121.99, 113.62, 111.97, 110.52, 108.52, 102.82, 101.62, 55.05, 49.59, 48.97, 42.52, 36.76, 30.66, 26.97, 26.68.

***Compound 21.***

Dissolve **20** (50 mg, 0.092 mmol) in 10 mL of DCM. Add $BBr_3$ (227.94 mg, 0.92 mmol) dropwise at 0 °C, $N_2$ atmosphere. Then, transfer to room temperature and continue stirring for 3 h. After the reaction, water (5 mL) was added to quench the reaction, DCM extraction, anhydrous $Na_2SO_4$ drying, and vacuum removal of solvent. The crude product was purified by silica gel column chromatography (eluent: DCM / MeOH = 50/1 to 10/1) to obtain **21** (21.3 mg, 0.04 mmol, 43%) as a red solid. LCMS (ESI) calcd for $C_{35}H_{38}N_3O_2^+$[M$^+$] 532.2958, found 532.2950.

$^{1}$H NMR (600 MHz, MeOD) δ 8.45 (t, *J* = 13.5 Hz, 1H), 7.48 (d, *J* = 7.1 Hz, 1H), 7.38 (dt, *J* = 14.3, 7.9 Hz, 2H), 7.28 (d, *J* = 7.9 Hz, 1H), 7.26 – 7.22 (m, 2H), 6.97 (d, *J* = 2.2 Hz, 1H), 6.87 (dd, *J* = 8.5, 2.2 Hz, 1H), 6.39 – 6.31 (m, 2H), 5.80 (ddd, *J* = 56.9, 53.1, 11.4 Hz, 6H), 5.34 (t, *J* = 4.9 Hz, 1H), 4.27 (s, 2H), 3.68 (s, 5H), 1.74 (d, *J* = 6.9 Hz, 12H).

$^{13}$C NMR (151 MHz, MeOD) δ 174.61, 173.75, 167.79, 156.90, 149.35, 142.74, 142.36, 140.37, 137.90, 136.29, 134.88, 131.46, 129.84, 129.44, 128.41, 124.58, 121.96, 114.88, 112.17, 110.36, 109.50, 102.96, 101.32, 49.52, 48.83, 42.42, 36.73, 31.66, 27.01, 26.68.

***Compound Cy3-COT-EP.***

The synthesis route of **Cy3-COT-EP** is similar to **Cy3-EP**, except **21** (20 mg, 0.037 mmol)

were used. **Cy3-COT-EP** (12.3 mg, 0.021 mmol, 58%) was obtained as a red solid. LCMS (ESI) calcd for $C_{38}H_{42}N_3O_2^+$[M+] 588.3221, found 588.3211.

$^1$H NMR (600 MHz, MeOD) δ 8.48 (t, *J* = 13.4 Hz, 1H), 7.51 – 7.45 (m, 2H), 7.40 (d, *J* = 7.7 Hz, 1H), 7.35 (s, 1H), 7.32 (s, 1H), 7.27 (t, *J* = 7.4 Hz, 1H), 7.23 (s, 1H), 7.05 (dd, *J* = 8.8, 2.2 Hz, 1H), 6.37 (dd, *J* = 23.4, 13.4 Hz, 2H), 5.82 (s, 6H), 5.33 (dd, *J* = 12.0, 7.2 Hz, 1H), 4.43 – 4.37 (m, 1H), 4.29 (s, 2H), 4.14 (s, 1H), 3.93 (dd, *J* = 11.3, 6.3 Hz, 1H), 3.69 (d, *J* = 14.5 Hz, 5H), 2.90 (t, *J* = 4.5 Hz, 1H), 2.77 (dd, *J* = 4.8, 2.6 Hz, 1H), 1.75 (s, 12H).

$^{13}$C NMR (151 MHz, MeOD) δ 174.87, 174.42, 167.80, 157.93, 149.80, 142.54, 142.27, 136.34, 131.47, 129.83, 129.65, 129.44, 128.46, 127.14, 124.81, 122.01, 114.52, 111.95, 110.58, 109.37, 102.80, 101.77, 69.65, 49.84, 49.57, 43.45, 36.78, 31.67, 29.35, 28.93, 26.95, 26.69, 25.51, 22.33.

***Compound 22.***

The synthesis route of **22** is similar to **12**, except N-(3-(Phenylamino) Allylidene) Aniline **was** used. **22** (52 mg, 0.09 mmol, 45%) was obtained as a purple-black solid. LCMS (ESI) calcd for $C_{34}H_{42}N_3O_4^+$[M+] 556.31698, found 556.31573.

$^1$H NMR (600 MHz, MeOD) δ 8.42 – 8.34 (m, 1H), 8.27 (t, *J* = 13.0 Hz, 1H), 8.18 – 8.02 (m, 2H), 7.54 (d, *J* = 7.4 Hz, 1H), 7.46 (d, *J* = 4.1 Hz, 2H), 7.35 (dd, *J* = 7.7, 3.7 Hz, 1H), 7.27 (d, *J* = 8.4 Hz, 1H), 6.71 (t, *J* = 12.4 Hz, 1H), 6.55 (d, *J* = 14.0 Hz, 1H), 6.23 (d, *J* = 13.3 Hz, 1H), 4.46 – 4.21 (m, 2H), 3.63 (d, *J* = 40.5 Hz, 3H), 3.58 – 3.47 (m, 2H), 1.93 – 1.62 (m, 12H), 1.29 – 1.17 (m, 9H).

$^{13}$C NMR (151 MHz, MeOD) δ 158.27, 157.06, 154.39, 143.85, 143.27, 142.04, 132.18, 130.90, 130.82, 129.71, 127.78, 127.17, 124.41, 123.47, 113.12, 110.59, 106.76, 103.97, 80.26, 51.38, 45.98, 39.29, 36.57, 33.09, 28.64, 28.05, 27.56, 23.77.

***Compound 23.***

The synthesis route of **23** is similar to **13**, except **22** (50 mg, 0.09 mmol) were used. **23** (29.3 mg, 0.05 mmol, 56%) was obtained as a purple-black solid. LCMS (ESI) calcd for $C_{38}H_{40}N_3O_3^+$[M+] 586.3064, found 586.3037.

$^1$H NMR (600 MHz, MeOD) δ 8.38 – 8.32 (m, 1H), 8.26 (t, *J* = 13.1 Hz, 1H), 8.10 (d, *J* = 8.5 Hz, 1H), 8.05 (s, 1H), 7.56 (d, *J* = 7.4 Hz, 1H), 7.50 – 7.43 (m, 2H), 7.36 (t, *J* = 7.2 Hz, 1H), 7.28 (d, *J* = 8.3 Hz, 1H), 6.65 (t, *J* = 12.4 Hz, 2H), 6.50 (d, *J* = 14.1 Hz, 1H), 6.22 (d, *J* = 13.3 Hz, 1H), 5.99 – 5.70 (m, 6H), 4.41 (s, 2H), 3.75 (s, 2H), 3.59 (s, 3H), 1.75 (t, *J* = 6.9 Hz, 12H).

$^{13}$C NMR (151 MHz, MeOD) δ 178.30, 173.41, 169.13, 157.14, 154.34, 148.51, 143.35, 143.23, 142.11, 139.63, 137.49, 136.14, 134.35, 132.88, 132.33, 131.26, 129.96, 129.91, 129.36, 128.09, 127.34, 124.46, 123.58, 112.93, 110.65, 107.01, 103.91, 51.48, 44.74, 38.44, 28.03, 27.59.

***Compound HZ Mito Deep Red.***

The synthesis route of ***HZ Mito Deep Red*** is similar to ***HZ Mito Red,*** except **23** (25 mg, 0.04 mmol) were used. ***HZ Mito Deep Red*** (21.6 mg, 0.03 mmol, 75%) was obtained as a purple-black solid. LCMS (ESI) calcd for $C_{43}H_{49}ClN_5O_3^+$[M+] 718.3518, found 718.3516.

$^1$H NMR (600 MHz, MeOD) δ 8.39 – 8.32 (m, 1H), 8.27 (t, *J* = 13.0 Hz, 1H), 8.01 – 7.91 (m, 2H),

7.55 (d, *J* = 7.3 Hz, 1H), 7.50 – 7.42 (m, 2H), 7.35 (s, 1H), 7.30 (d, *J* = 8.3 Hz, 1H), 6.65 (d, *J* = 4.9 Hz, 2H), 6.48 (d, *J* = 14.0 Hz, 1H), 6.23 (d, *J* = 13.3 Hz, 1H), 5.80 (dd, *J* = 34.2, 21.2 Hz, 6H), 5.37 (d, *J* = 4.7 Hz, 1H), 4.39 (s, 2H), 4.10 (s, 2H), 3.71 (d, *J* = 41.8 Hz, 2H), 3.62 (d, *J* = 21.1 Hz, 3H), 3.47 (t, *J* = 6.5 Hz, 2H), 3.37 (s, 2H), 1.98 – 1.66 (m, 14H).

$^{13}$C NMR (151 MHz, MeOD) δ 169.62, 169.17, 157.00, 154.64, 147.39, 143.47, 143.16, 142.33, 139.65, 137.56, 136.19, 134.39, 132.95, 132.91, 131.33, 130.95, 130.87, 129.92, 129.60, 127.96, 127.18, 123.59, 122.42, 112.81, 110.88, 106.66, 104.01, 51.38, 43.29, 38.32, 36.61, 33.14, 30.86, 30.30, 28.05, 27.70, 27.00, 23.82.

## Reference


[1] K. Amirbekyan, N. Duchemin, E. Benedetti, R. Joseph, A. Colon, S. A. Markarian, L. Bethge, S. Vonhoff, S. Klussmann, J. Cossy, J.-J. Vasseur, S. Arseniyadis, M. Smietana, *ACS Catal.* **2016**, *6*, 3096–3105.

[2] A.-M. Fanning, S. E. Plush, T. Gunnlaugsson, *Chem. Commun.* **2006**, *36*, 3791-3793.

[3] A. Nano, A. N. Boynton, J. K. Barton, *J. Am. Chem. Soc.* **2017**, *139*, 17301–17304.

[4] H. Fujioka, S. Uno, M. Kamiya, R. Kojima, K. Johnsson, Y. Urano, *Chem. Commun.* **2020**, *56*, 5617–5620.

[5] Z. Yang, L. Li, J. Ling, T. Liu, X. Huang, Y. Ying, Y. Zhao, Y. Zhao, K. Lei, L. Chen, Z. Chen, *Chem. Sci.* **2020**, *11*, 8506–8516.

[6] A. K. Pati, O. El Bakouri, S. Jockusch, Z. Zhou, R. B. Altman, G. A. Fitzgerald, W. B. Asher, D. S. Terry, A. Borgia, M. D. Holsey, J. E. Batchelder, C. Abeywickrama, B. Huddle, D. Rufa, J. A. Javitch, H. Ottosson, S. C. Blanchard, *Proc. Natl. Acad. Sci. U.S.A.* **2020**, *117*, 24305–24315.

[7] H. M. Altass, M. Morad, A. S. Khder, M. Raafat, R. I. Alsantali, M. A. Khder, R. S. Salama, M. Shaheer Malik, Z. Moussa, M. A. S. Abourehab, S. A. Ahmed, *Arabian Journal of Chemistry* **2022**, *15*, 103670.

[8] P. Gaur, A. Kumar, G. Dey, R. Kumar, S. Bhattacharyya, S. Ghosh, *ACS Appl. Mater. Interfaces* **2016**, *8*, 10690–10699.

[9] A. Levitz, S. T. Ladani, D. Hamelberg, M. Henary, *Dyes and Pigments* **2014**, *105*, 238–249.

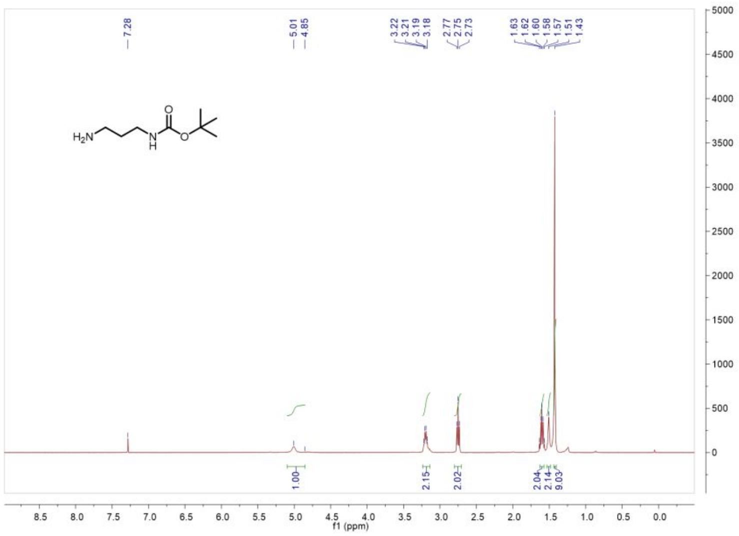

Fig.S18. The $^{1}H$ NMR spectrum of 1 in $CDCl_3$-d.

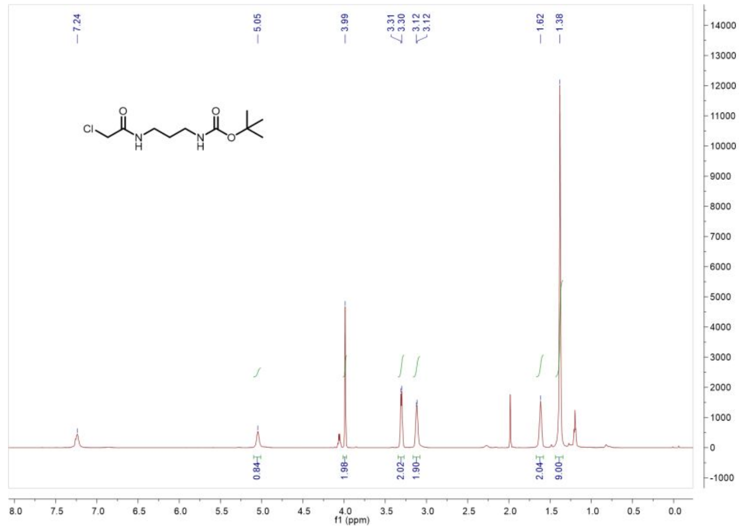

Fig.S19. The 1H NMR spectrum of 2 in CDCl3-d.

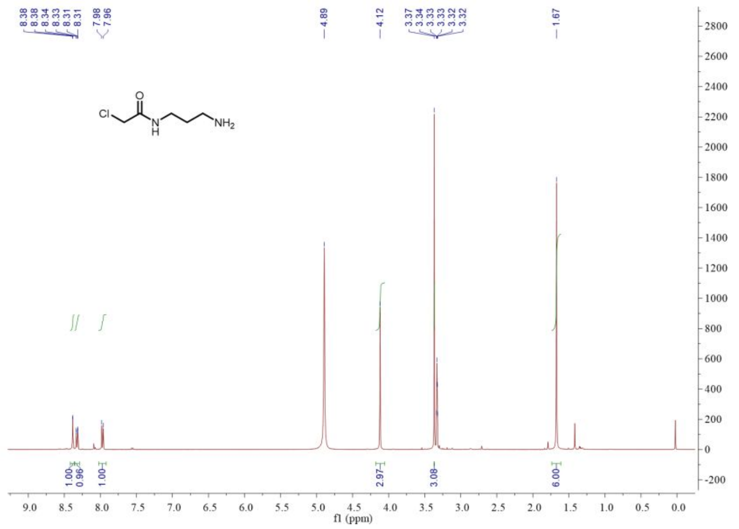

Fig.S20. The $^{1}H$ NMR spectrum of 3 in MeOD-$d_4$.

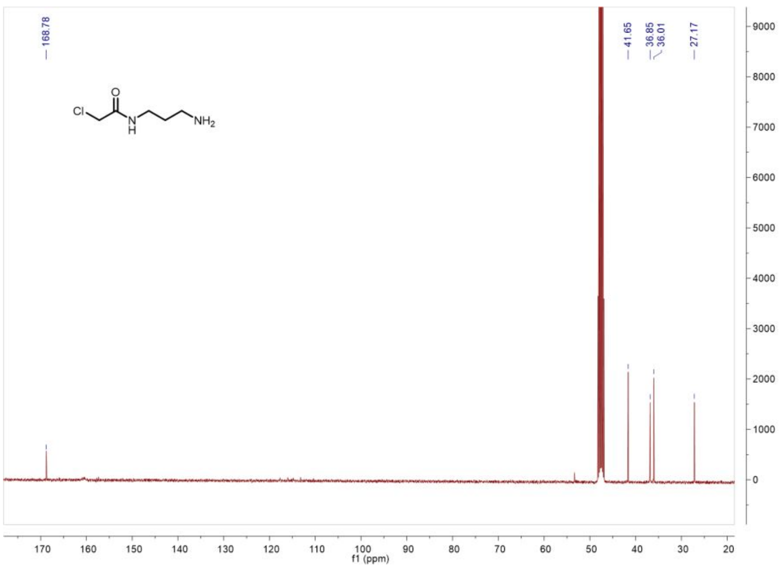


Fig.S21. The $^{13}$C NMR spectrum of 3 in MeOD-$d_4$.

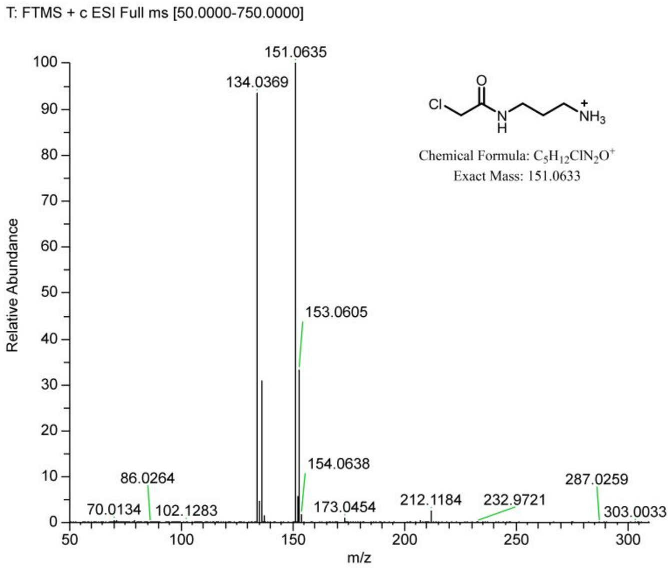


Fig.S22. The HR-MS spectrum of 3.

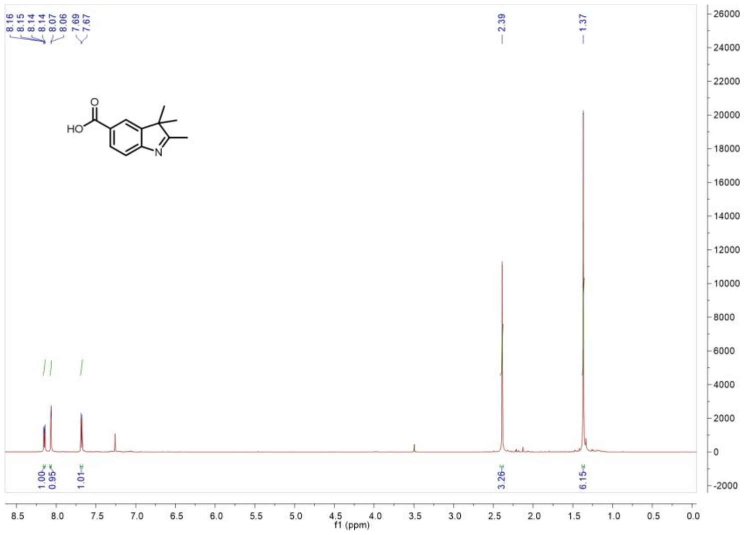


Fig.S23. The $^{1}$H NMR spectrum of 4 in $CDCl_3$-d.

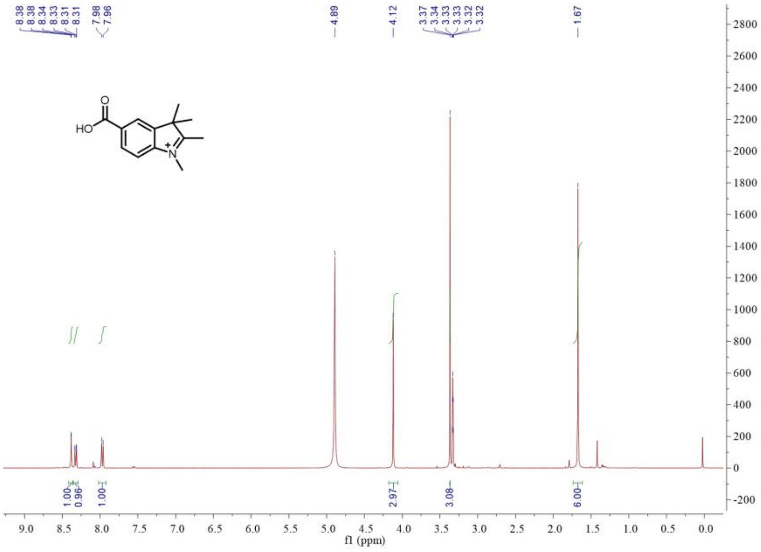


Fig.S24. The $^1$H NMR spectrum of 5 in MeOD-$d_4$.

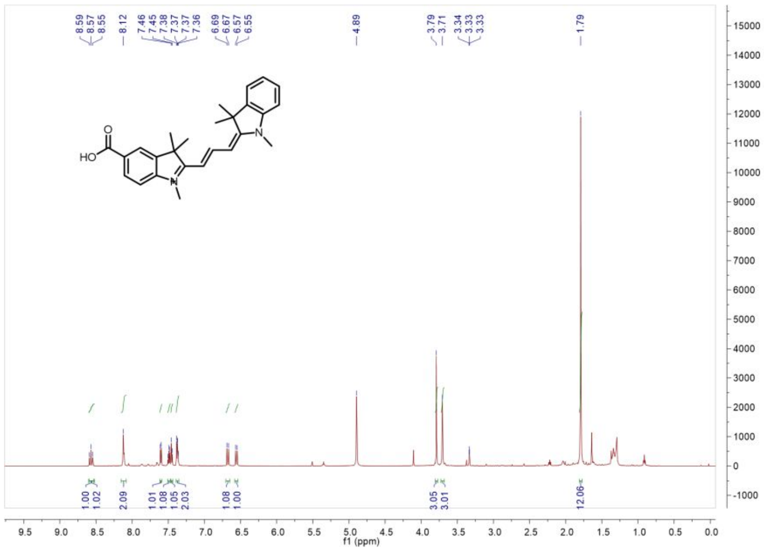


Fig.S25. The $^1$H NMR spectrum of 6 in MeOD-$d_4$.

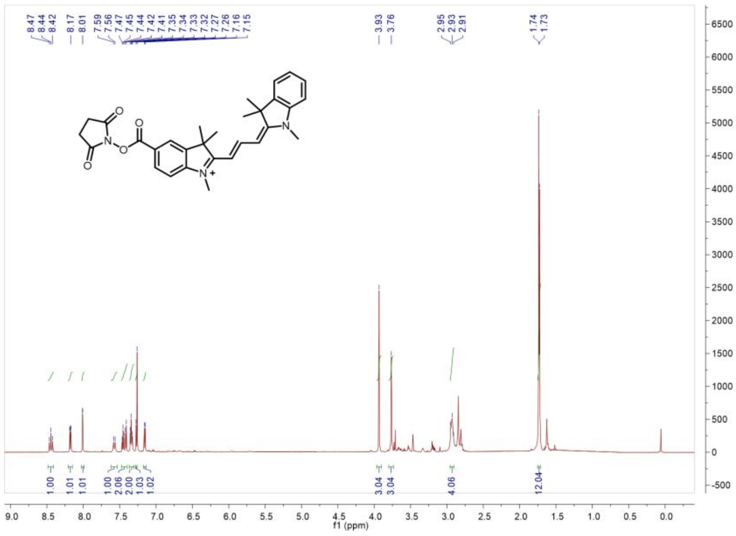


Fig.S26. The $^1$H NMR spectrum of Cy3-NHS in $CDCl_3$-d.

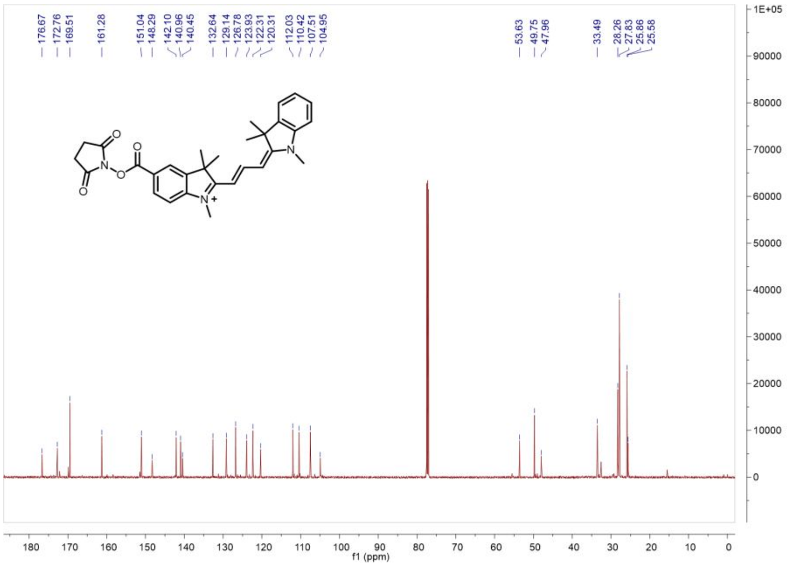


Fig.S27. The $^{13}$C NMR spectrum of Cy3-NHS in $CDCl_3$-d.

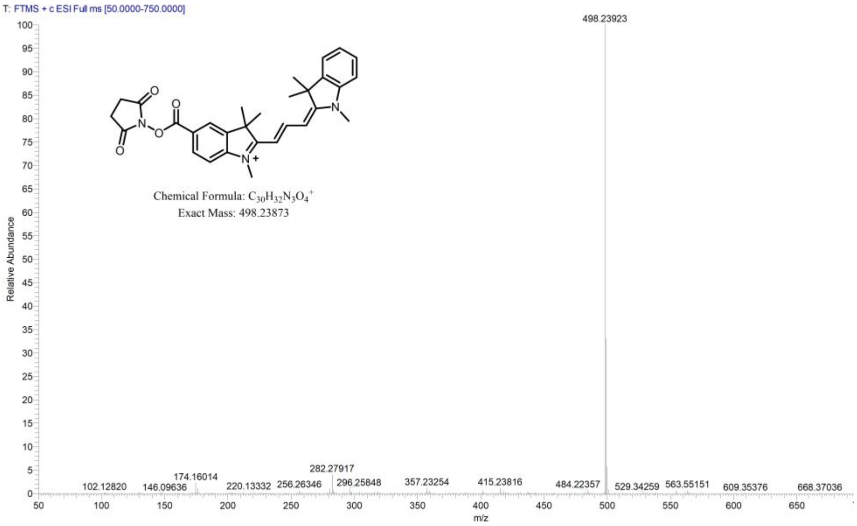


Fig.S28. The HR-MS spectrum of Cy3-NHS.

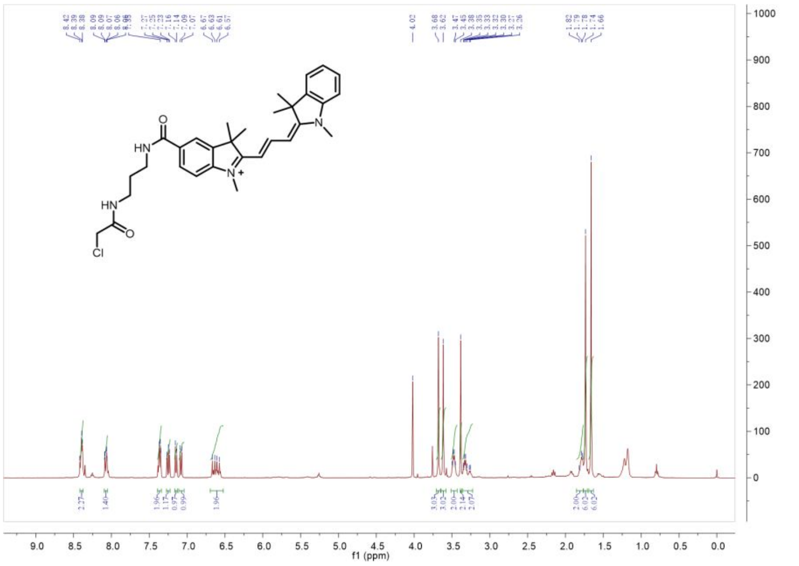


Fig.S29. The $^{1}$H NMR spectrum of Cy3-CA in $CDCl_3$-d.

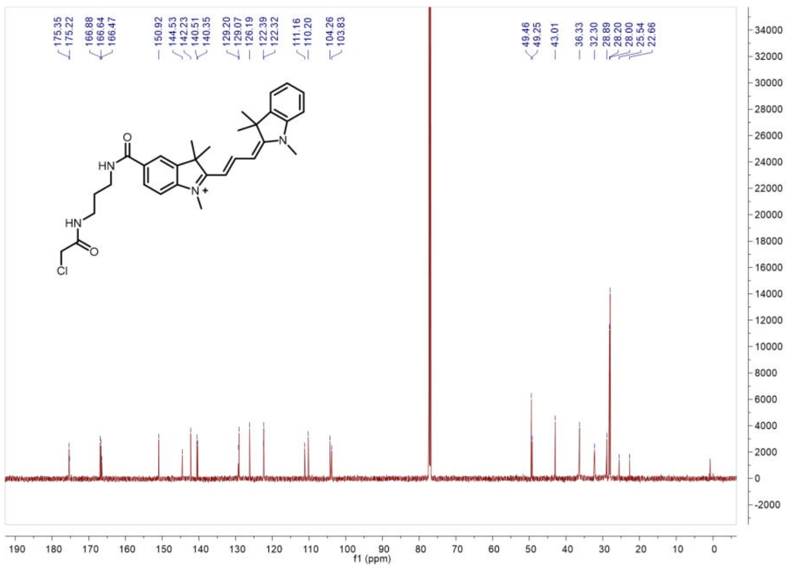


Fig.S30. The $^{13}C$ NMR spectrum of Cy3-CA in $CDCl_3$-d.

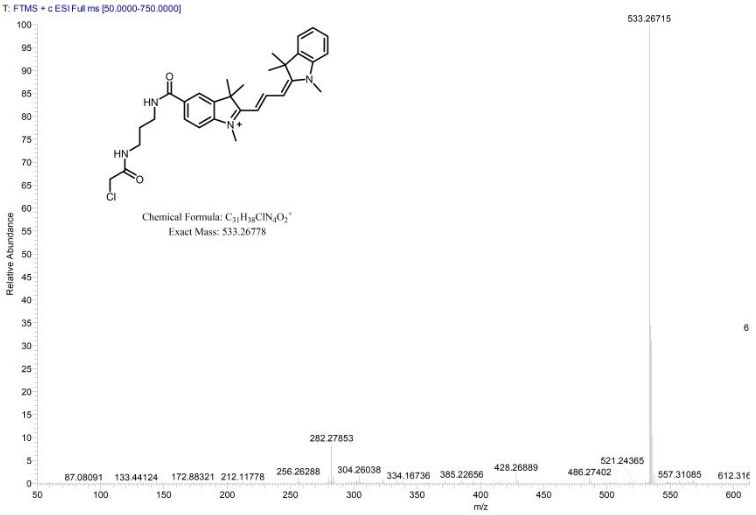


Fig.S31. The HR-MS spectrum of Cy3-CA.

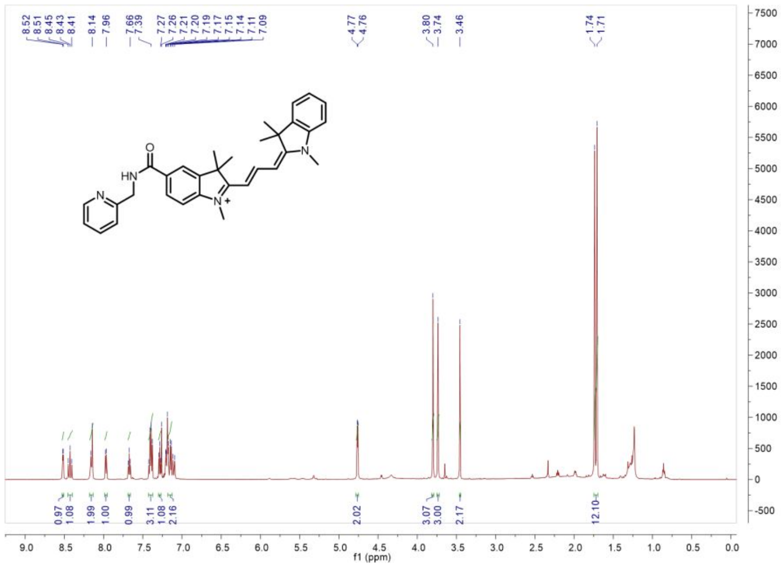


Fig.S32. The $^{1}H$ NMR spectrum of Cy3-Py in $CDCl_3$-d.

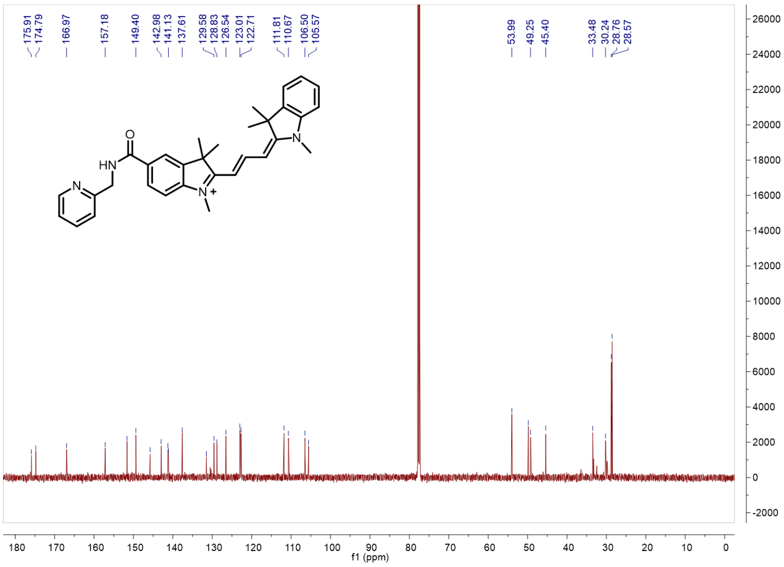


Fig.S33. The $^{13}$C NMR spectrum of Cy3-Py in $CDCl_3$-d.

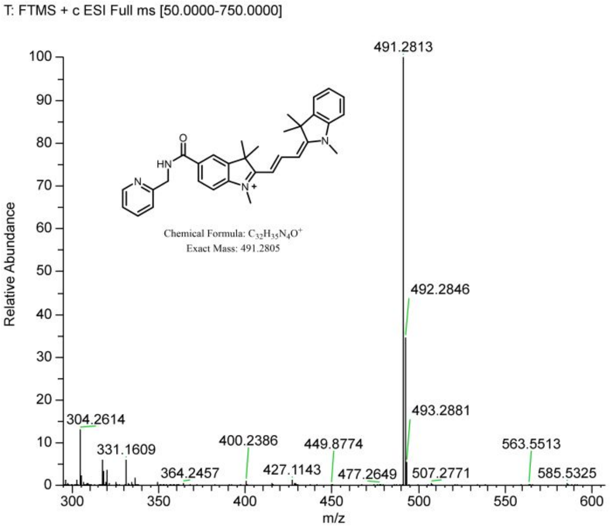


Fig.S34. The HR-MS spectrum of Cy3-Py.

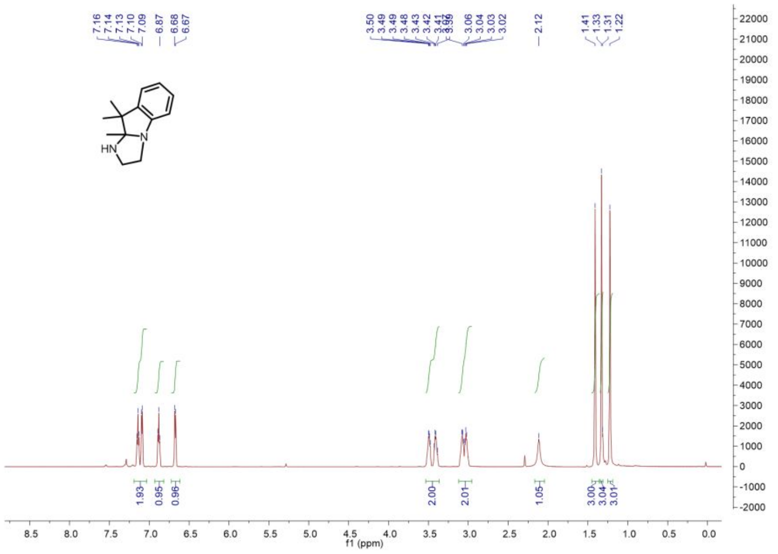


Fig.S35. The $^{1}$H NMR spectrum of 8 in $CDCl_3$-d.

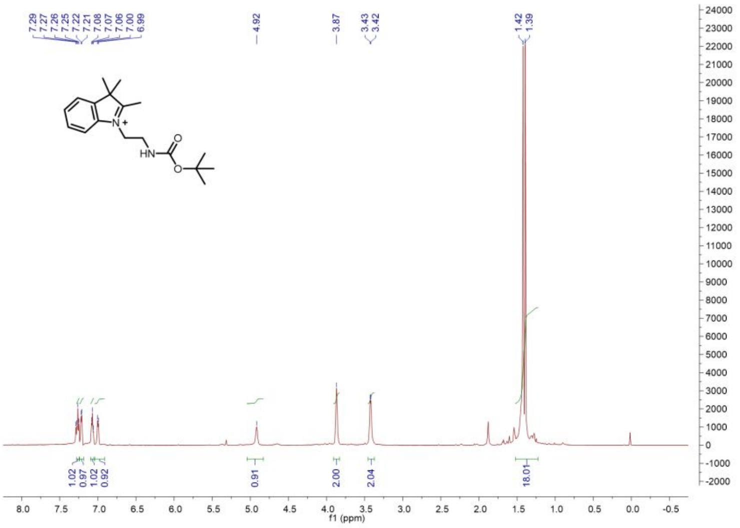


Fig.S36. The $^{1}$H NMR spectrum of 9 in $CDCl_3$-d.

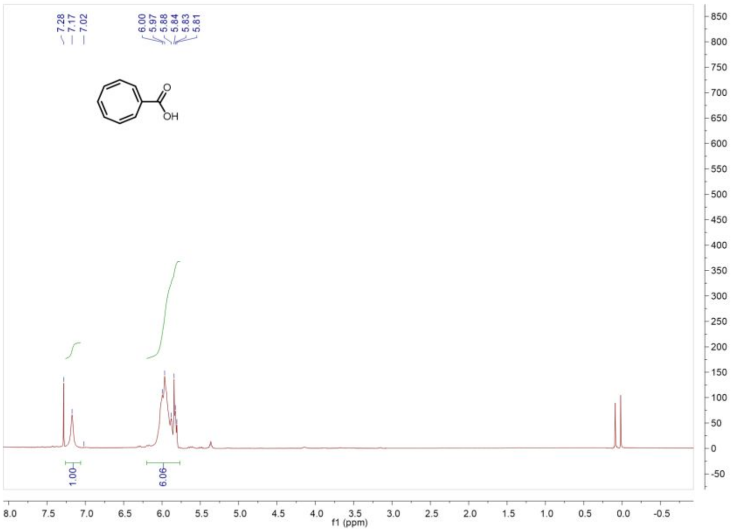


Fig.S37. The $^{1}$H NMR spectrum of 10 in $CDCl_3$-d.

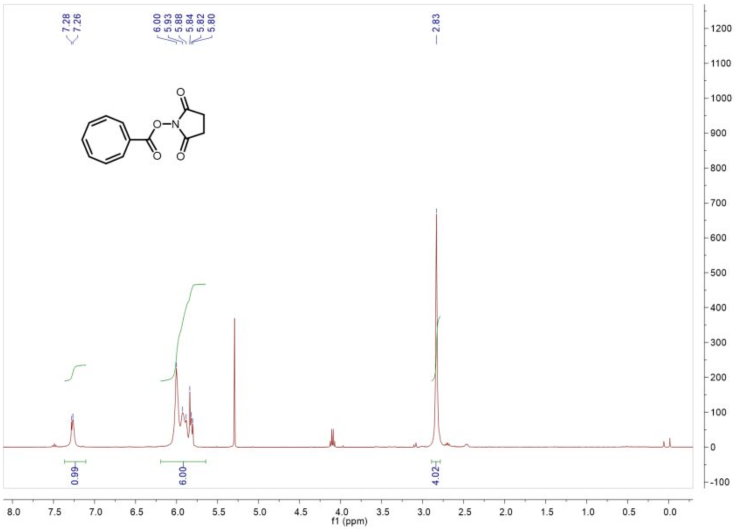


Fig.S38. The $^{1}$H NMR spectrum of 11 in $CDCl_3$-d.

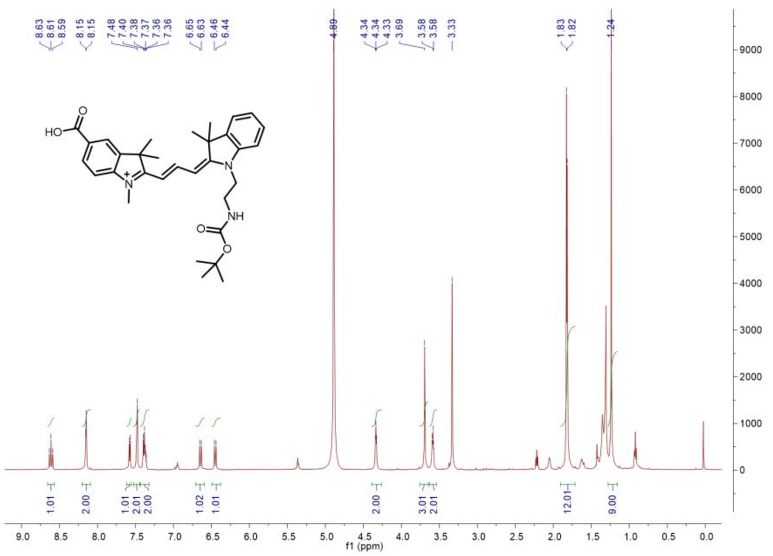


Fig.S39. The $^{1}H$ NMR spectrum of 12 in MeOD-$d_4$.

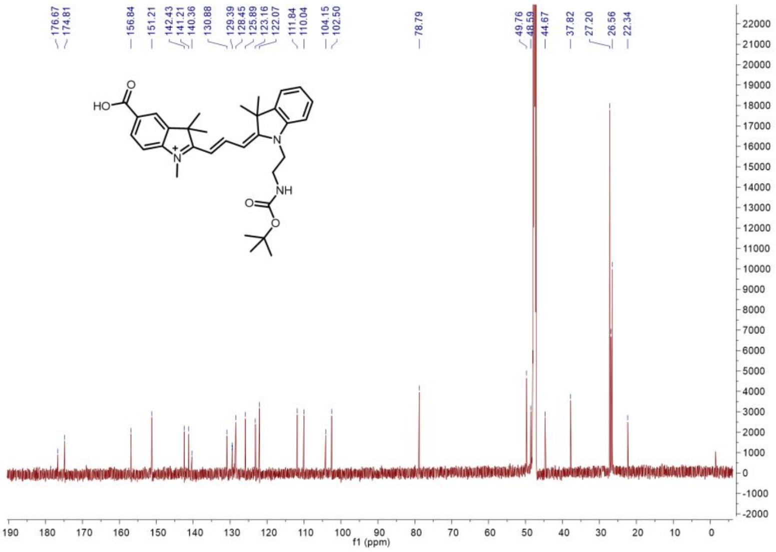


Fig.S40. The $^{13}C$ NMR spectrum of 12 in MeOD-$d_4$.

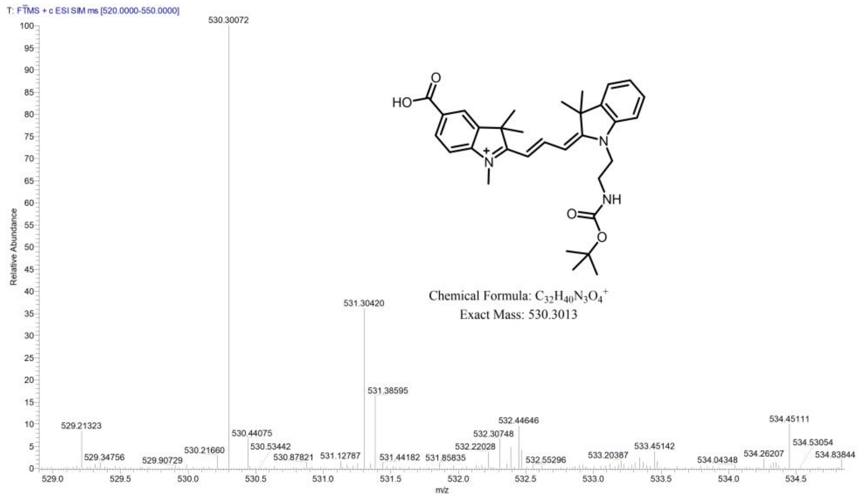


Fig.S41. The HR-MS spectrum of 12.

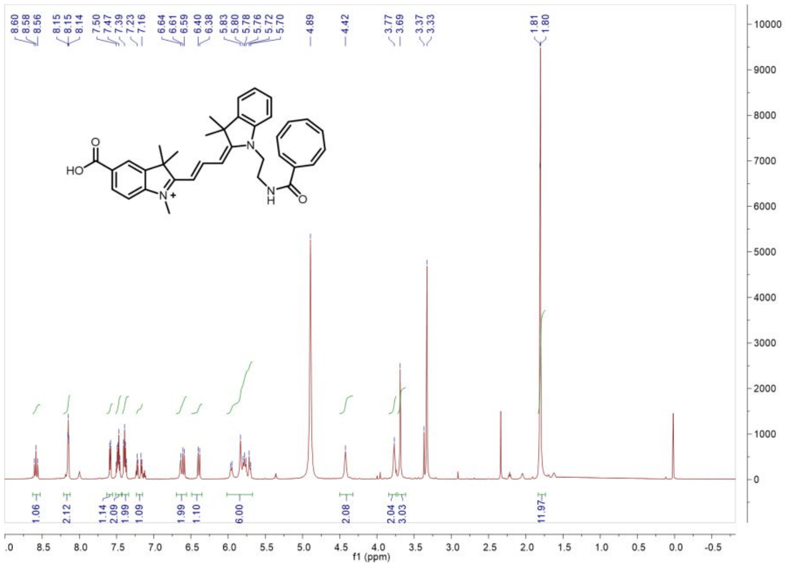

Fig.S42. The $^{1}$H NMR spectrum of 13 in MeOD-$d_4$.

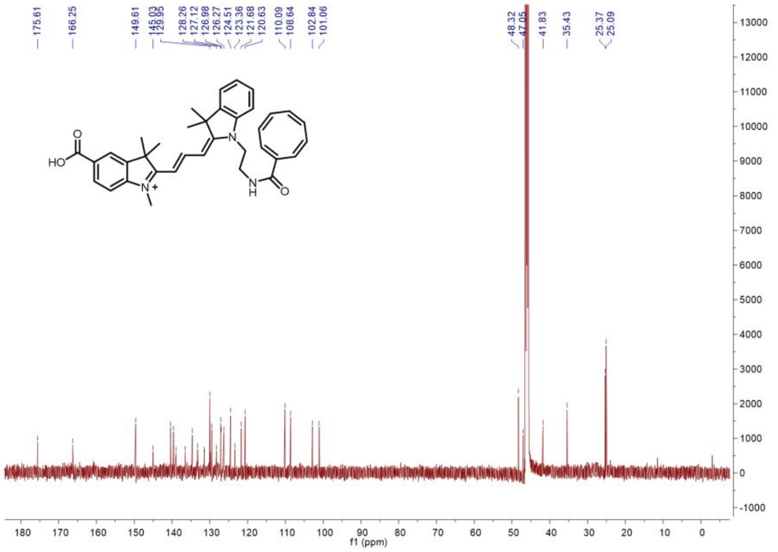

Fig.S43. The $^{13}$C NMR spectrum of 13 in MeOD-$d_4$.

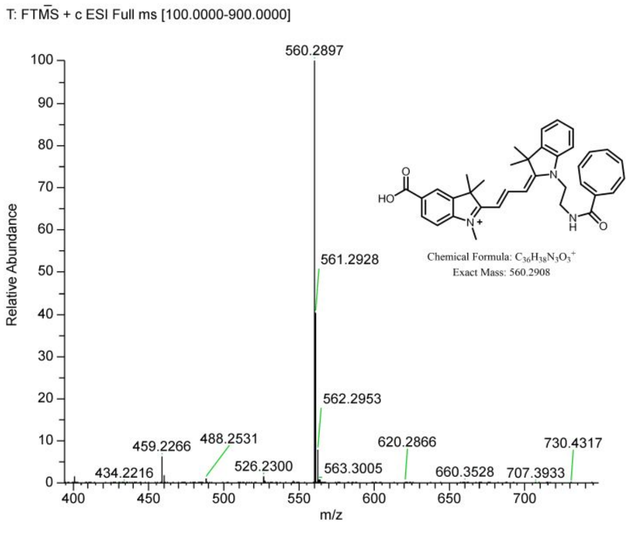


Fig.S44. The HR-MS spectrum of 13.

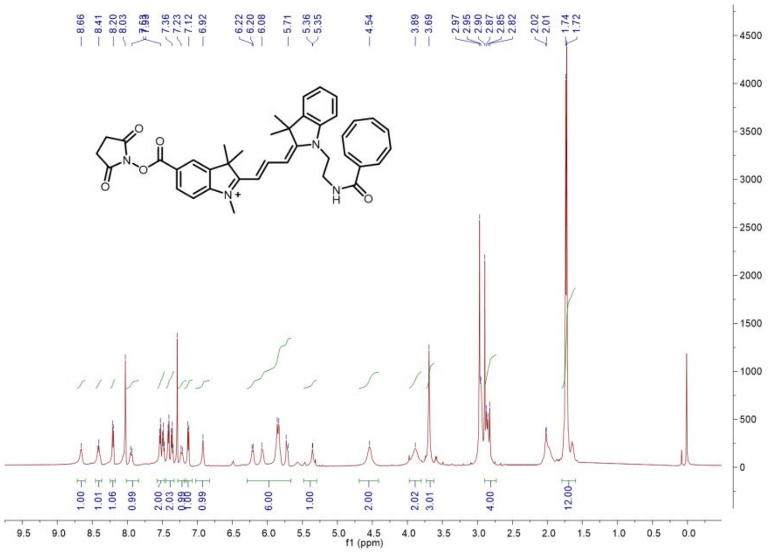

Fig.S45. The $^{1}H$ NMR spectrum of Cy3-COT-NHS in $CDCl_3$-d.

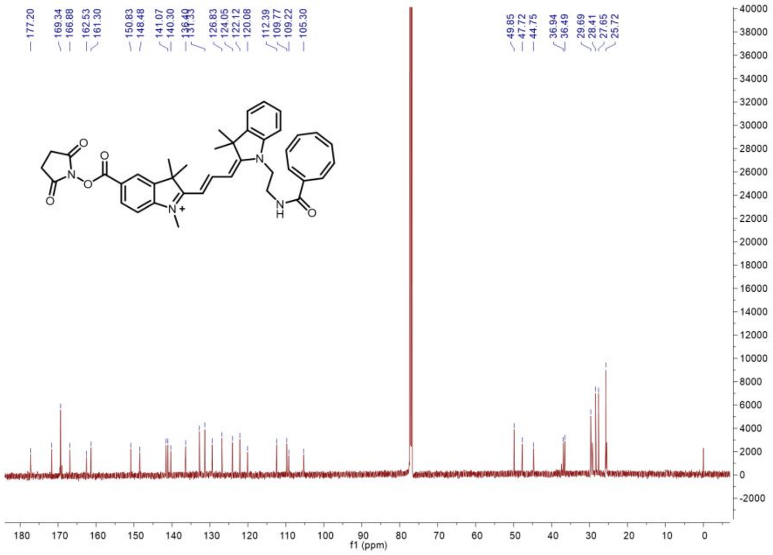

Fig.S46. The $^{13}C$ NMR spectrum of Cy3-COT-NHS in $CDCl_3$-d.

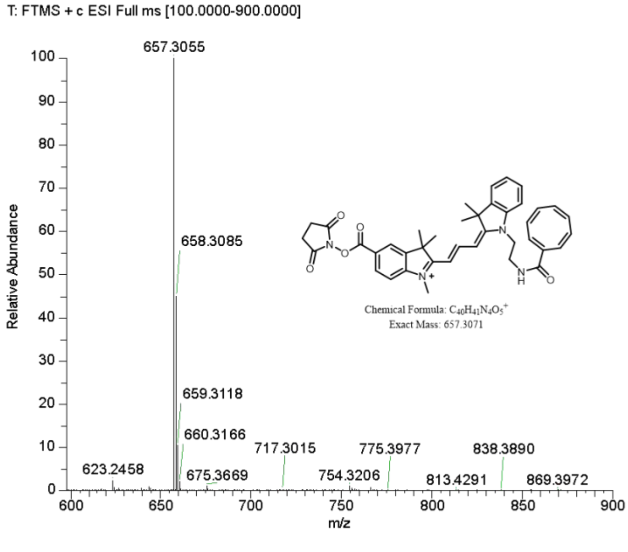


Fig.S47. The HR-MS spectrum of Cy3-COT-NHS.

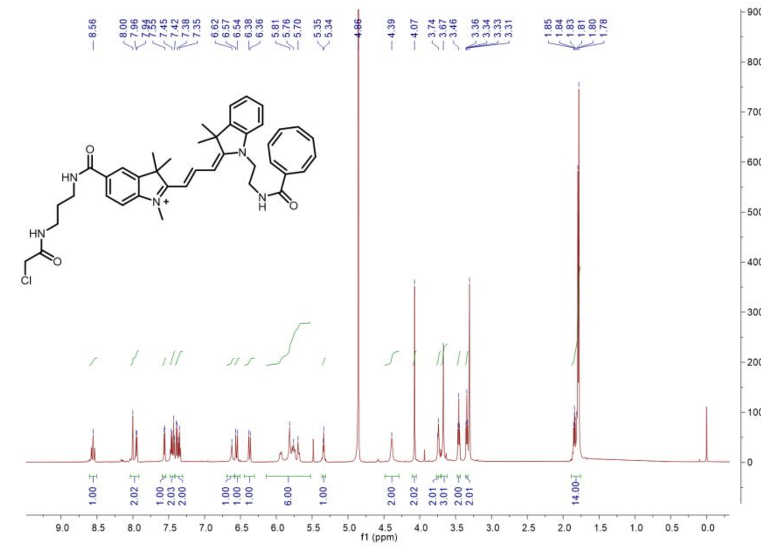


Fig.S48. The $^{1}H$ NMR spectrum of HZ Mito Red in MeOD-d$_4$.

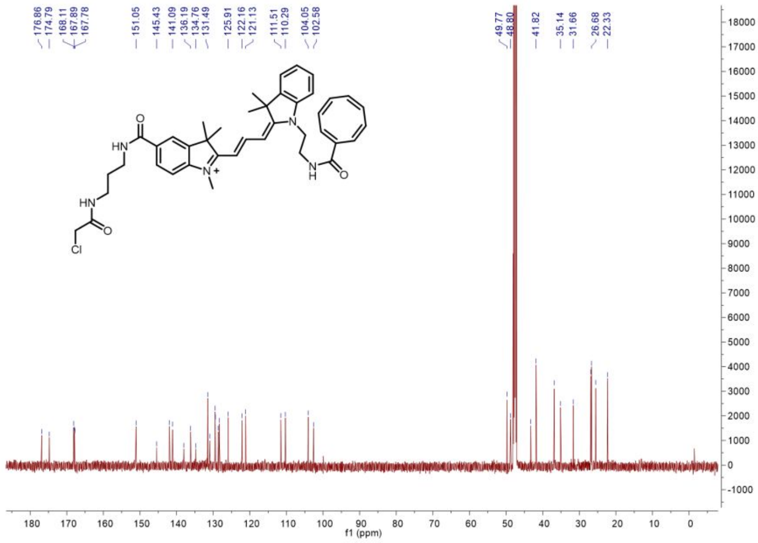


Fig.S49. The $^{13}C$ NMR spectrum of HZ Mito Red in MeOD-d$_4$.

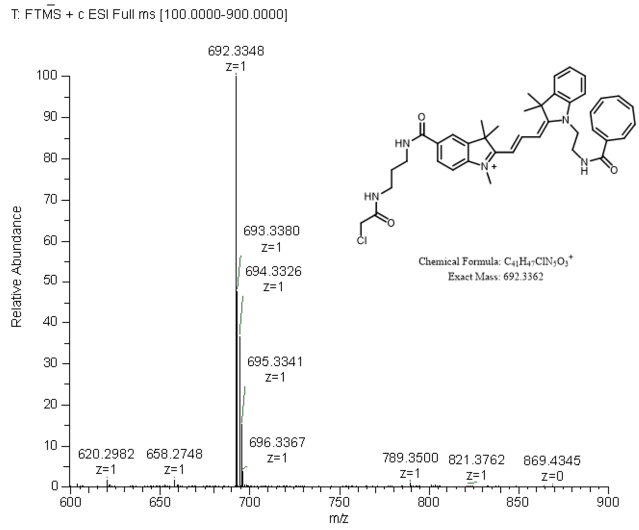


Fig.S50. The HR-MS spectrum of HZ Mito Red.

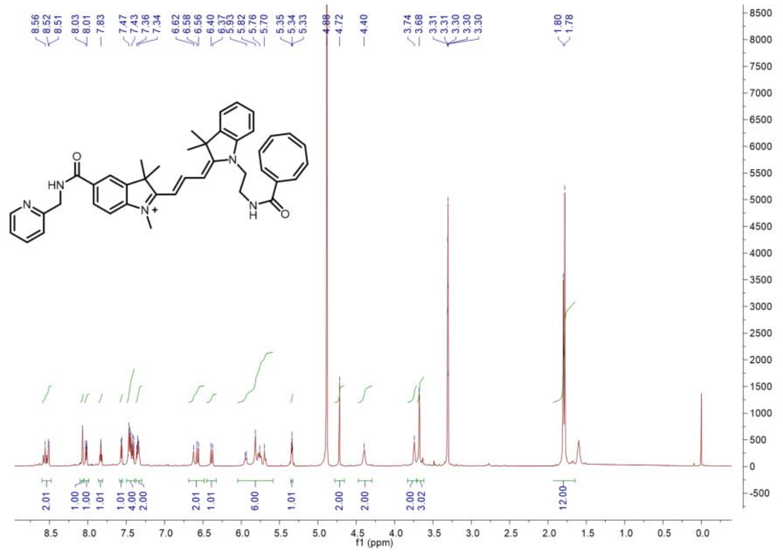

Fig.S51. The $^{1}H$ NMR spectrum of Cy3-COT-Py in MeOD-$d_4$.

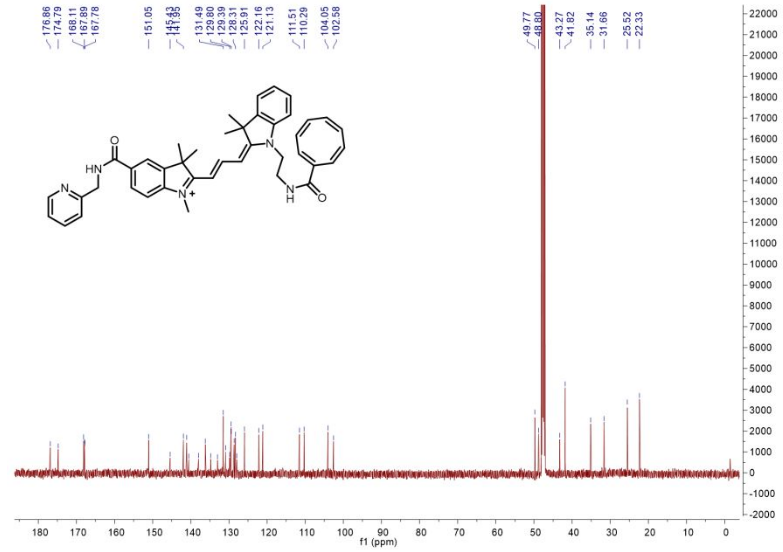

Fig.S52. The $^{13}C$ NMR spectrum of HZ Mito Red in MeOD-$d_4$.

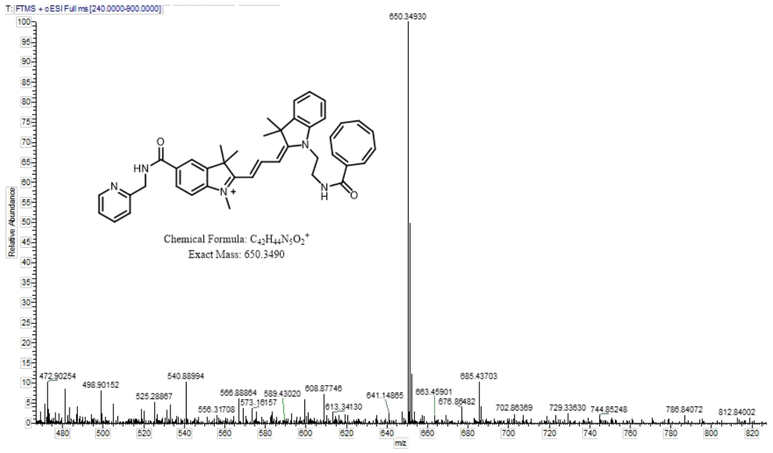


Fig.S53. The HR-MS spectrum of Cy3-COT-Py.

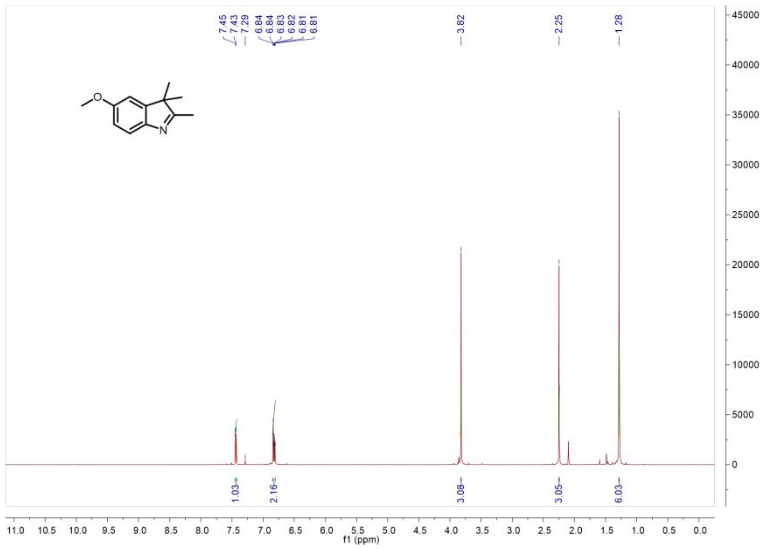

Fig.S54. The $^{1}$H NMR spectrum of 14 in $CDCl_3$-d.

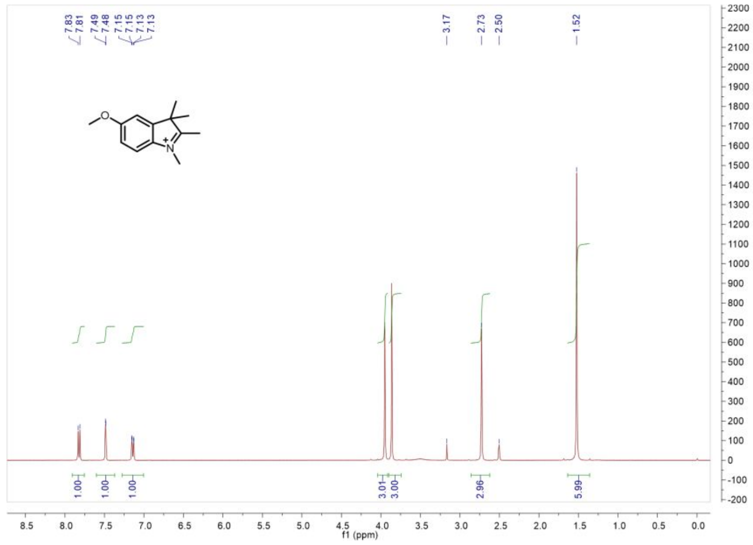

Fig.S55. The $^{1}$H NMR spectrum of 15 in DMSO-$d_4$.

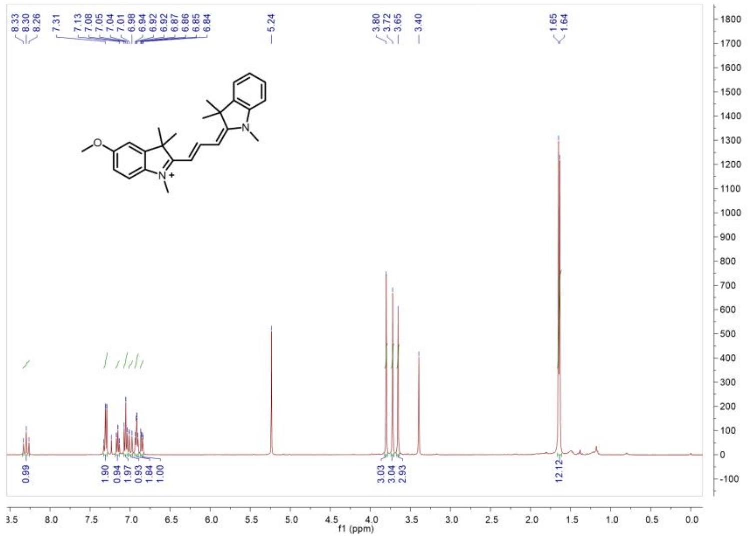

Fig.S56. The $^{1}$H NMR spectrum of 16 in MeOD-$d_4$.

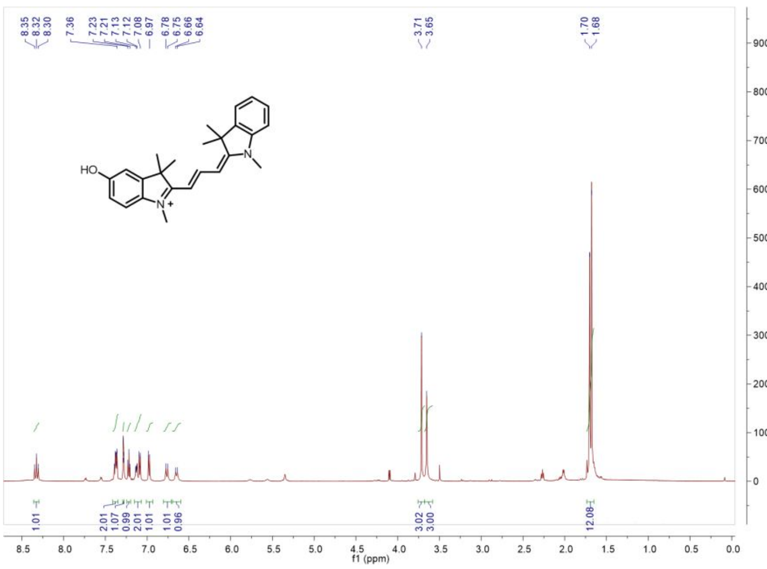


Fig.S57. The $^{1}$H NMR spectrum of 17 in $CDCl_3$-d.

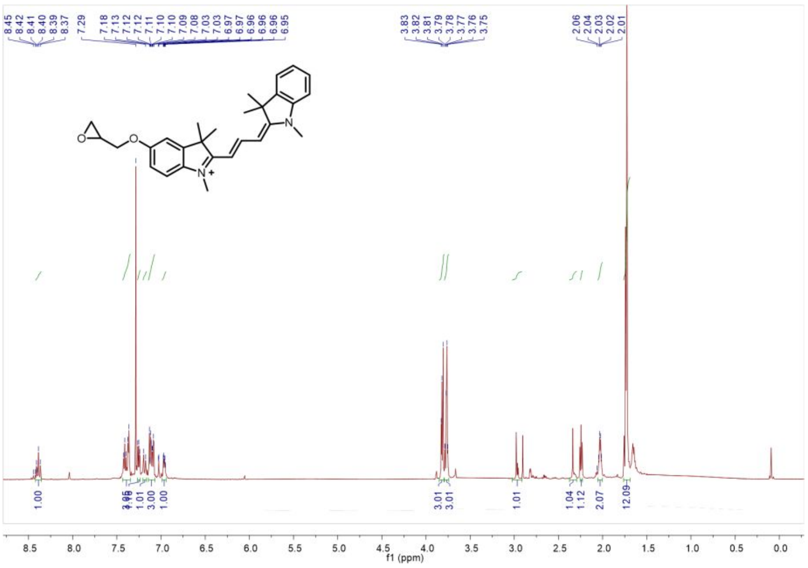


Fig.S58. The $^{1}$H NMR spectrum of Cy3-EP in $CDCl_3$-d.

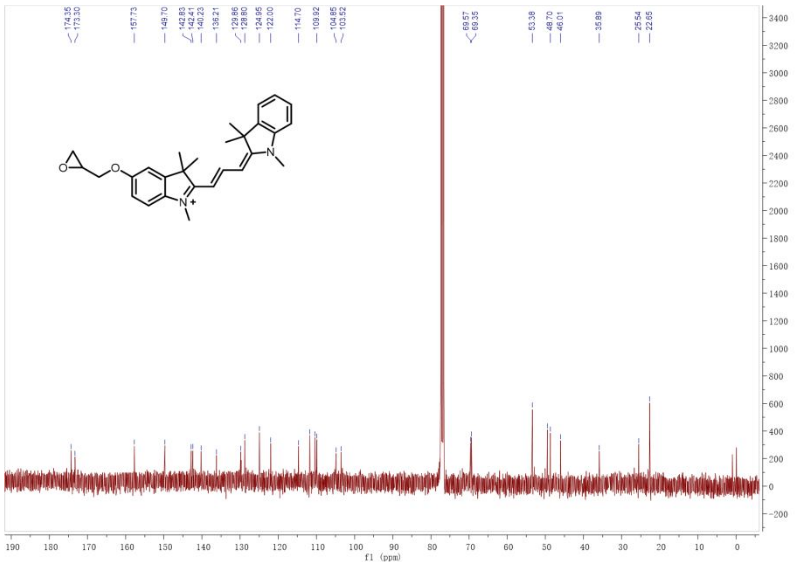


Fig.S59. The $^{13}$C NMR spectrum of Cy3-EP in $CDCl_3$-d.

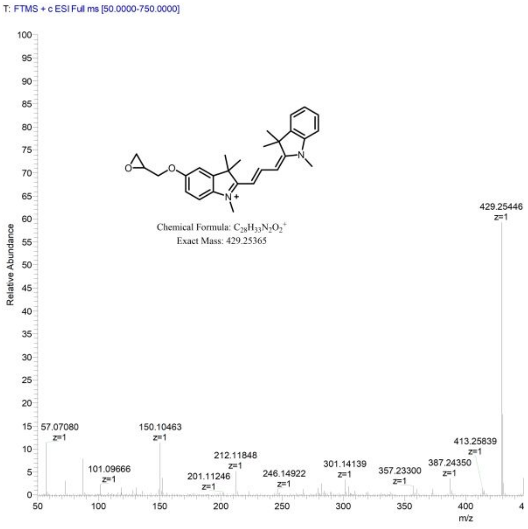


Fig.S60. The HR-MS spectrum of Cy3-EP.

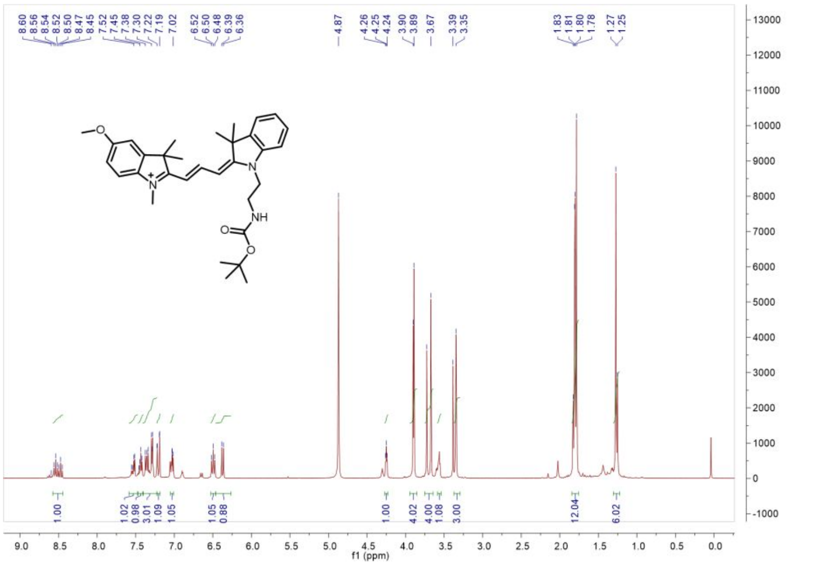


Fig.S61. The $^{1}H$ NMR spectrum of 18 in MeOD-$d_4$.

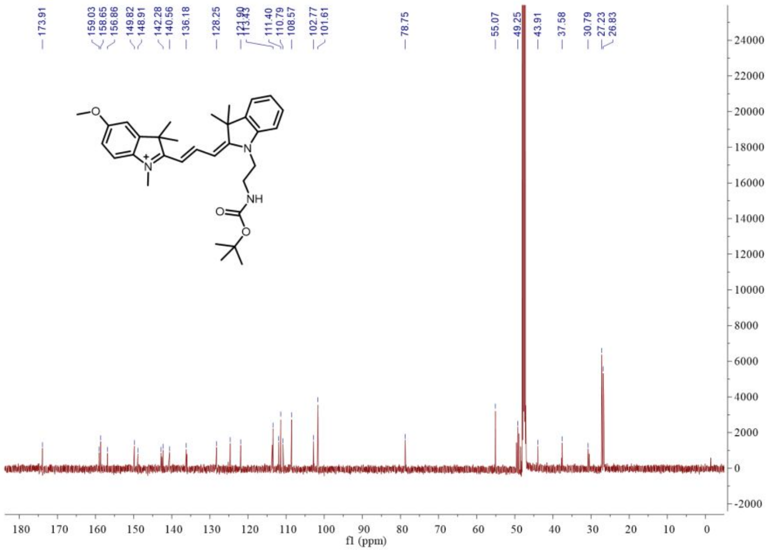


Fig.S62. The $^{13}C$ NMR spectrum of 18 in MeOD-$d_4$.

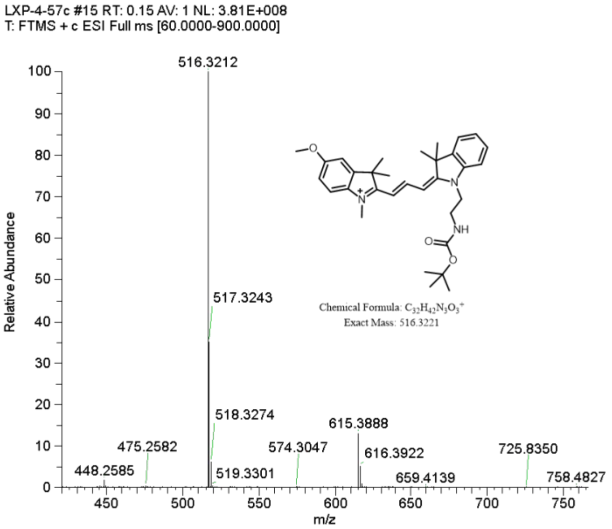


Fig.S63. The HR-MS spectrum of 18.

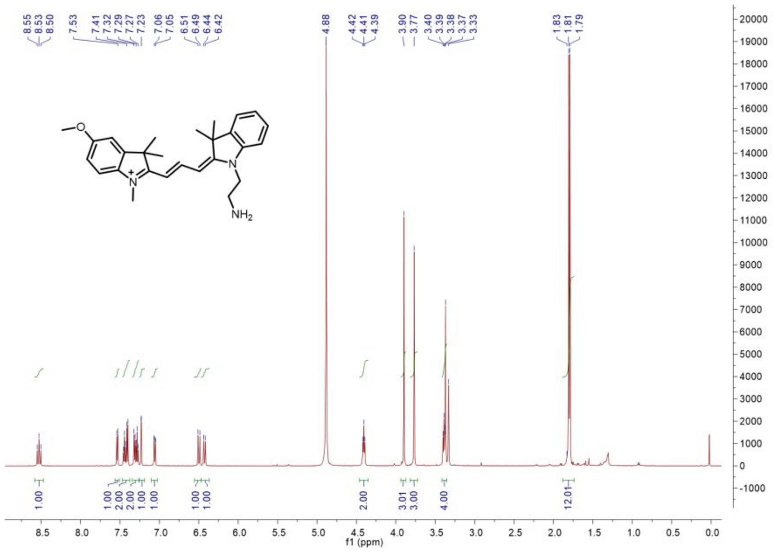

Fig.S64. The $^{1}H$ NMR spectrum of 19 in MeOD-$d_4$.

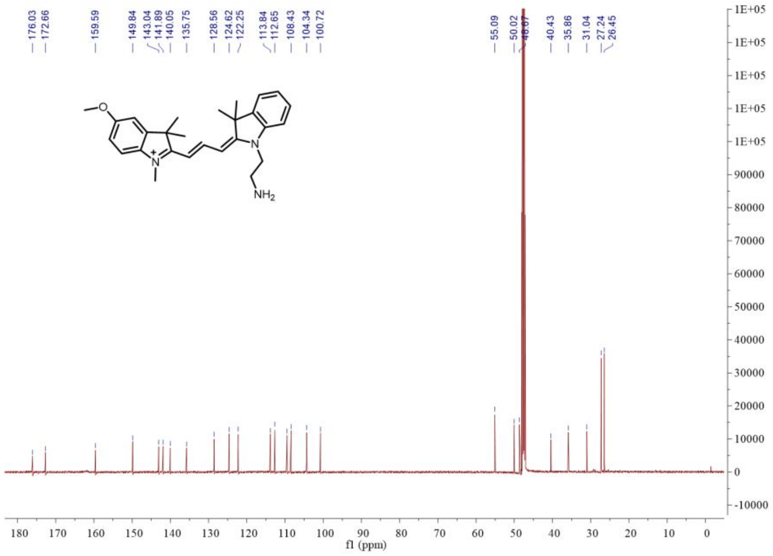

Fig.S65. The $^{13}C$ NMR spectrum of 19 in MeOD-$d_4$.

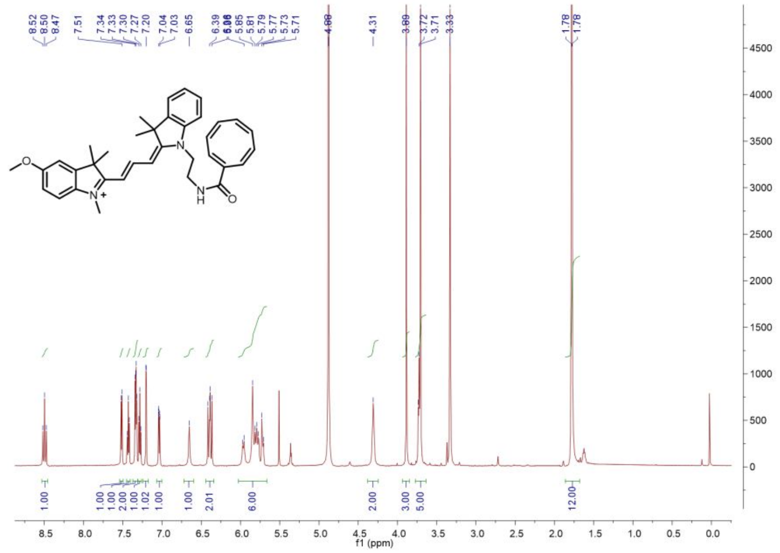


Fig.S66. The $^{1}$H NMR spectrum of 20 in MeOD-$d_4$.

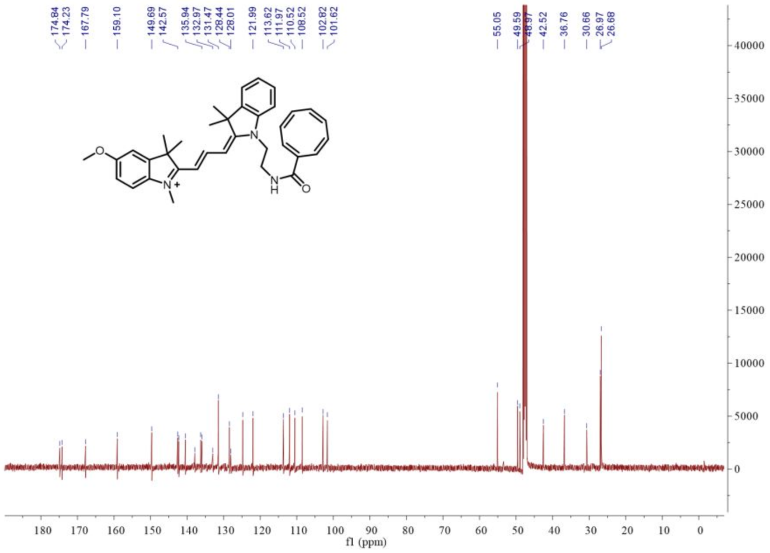


Fig.S67. The $^{13}$C NMR spectrum of 20 in MeOD-$d_4$.

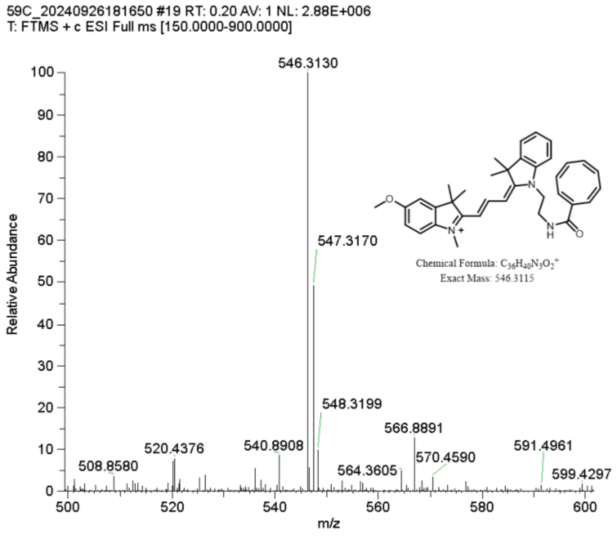


Fig.S68. The HR-MS spectrum of 20.

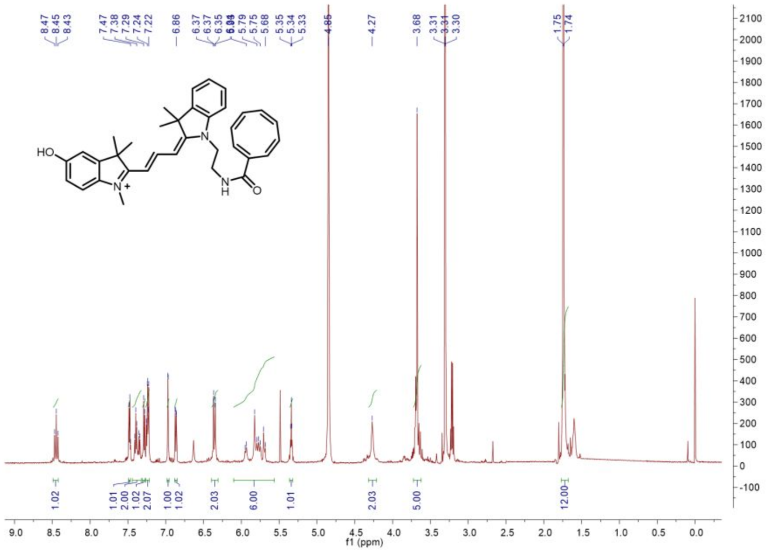

Fig.S69. The $^{1}H$ NMR spectrum of 21 in MeOD-$d_4$.

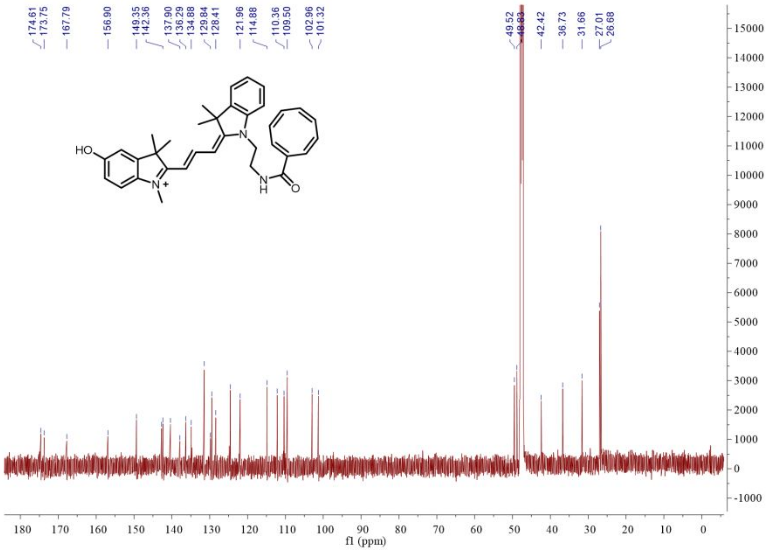

Fig.S70. The $^{13}C$ NMR spectrum of 21 in MeOD-$d_4$.

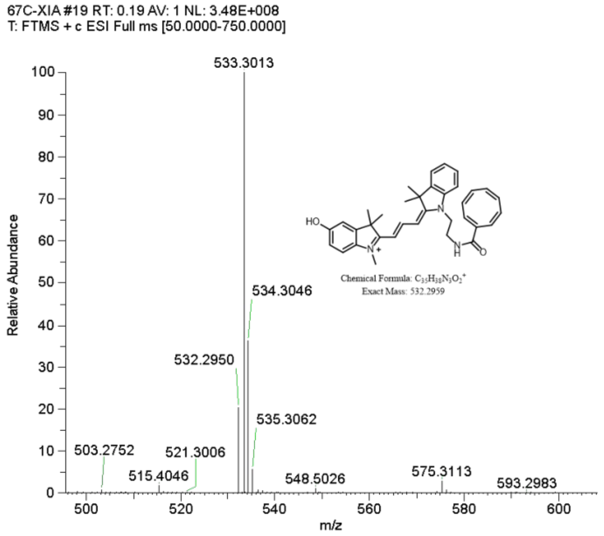


Fig.S71. The HR-MS spectrum of 21.

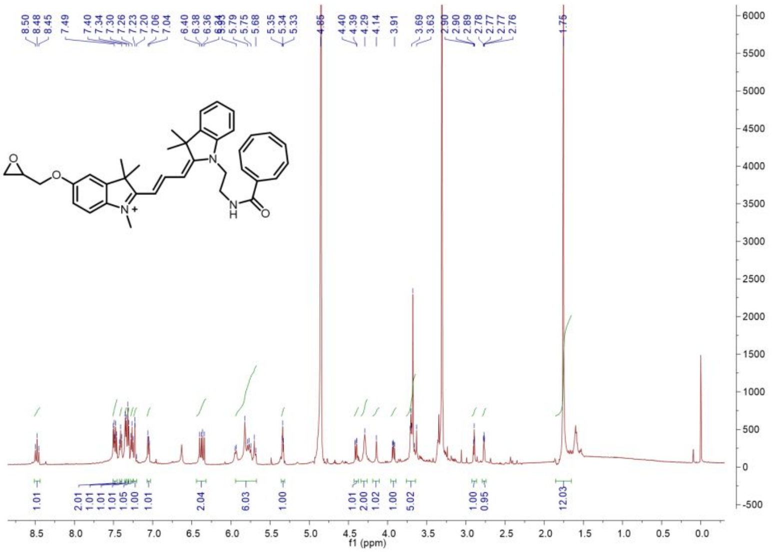


Fig.S72. The $^{1}H$ NMR spectrum of Cy3-COT-EP in MeOD-d$_4$.

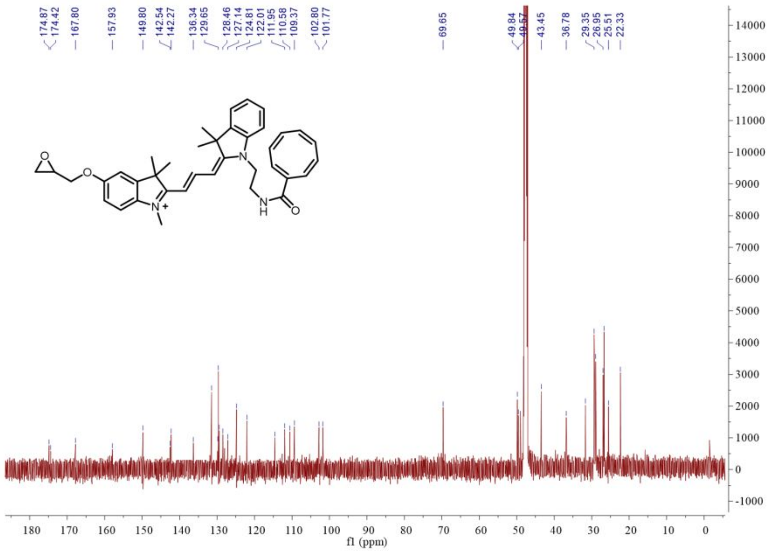


Fig.S73. The $^{13}C$ NMR spectrum of Cy3-COT-EP in MeOD-d$_4$.

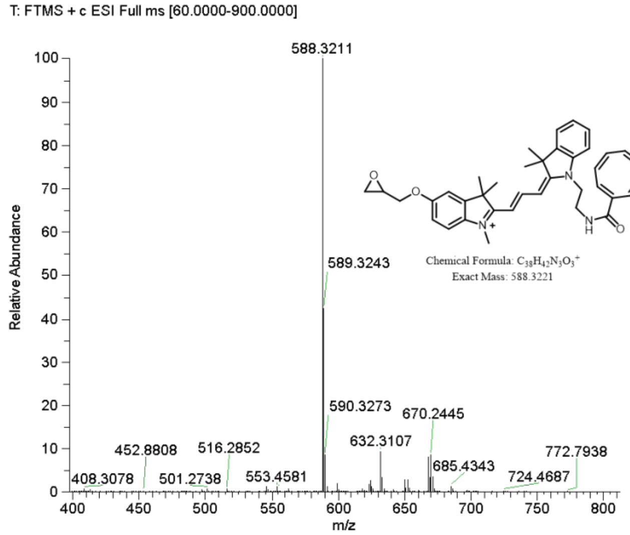


Fig.S74. The HR-MS spectrum of Cy3-COT-EP.

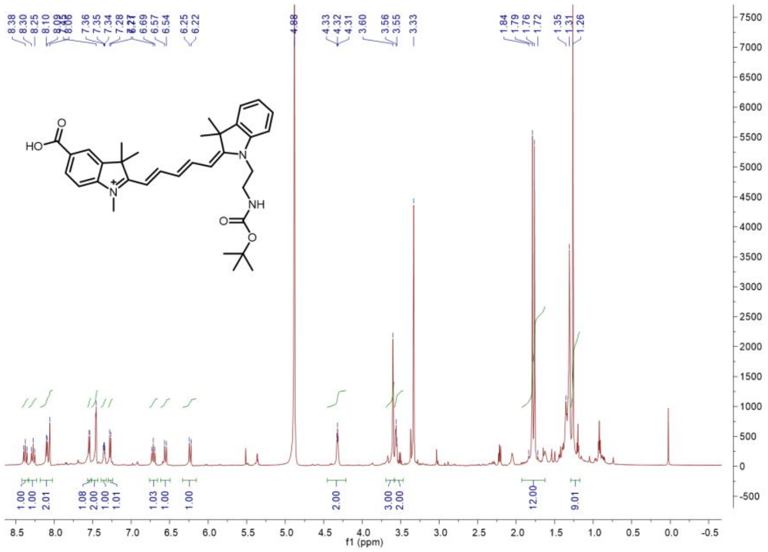

Fig.S75. The $^{1}H$ NMR spectrum of 22 in MeOD-$d_4$.

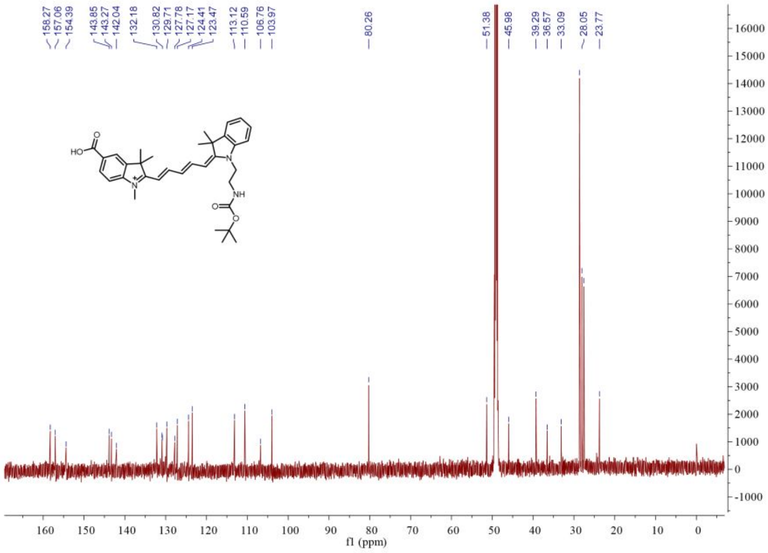

Fig.S76. The $^{13}C$ NMR spectrum of 22 in MeOD-$d_4$.

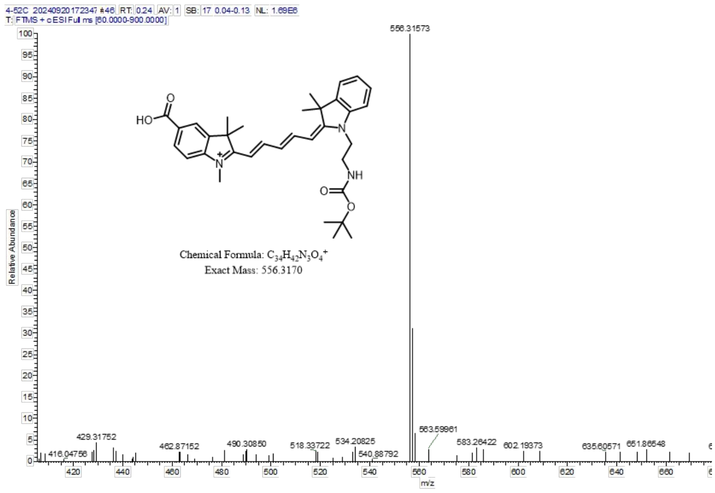

Fig.S77. The HR-MS spectrum of 22.

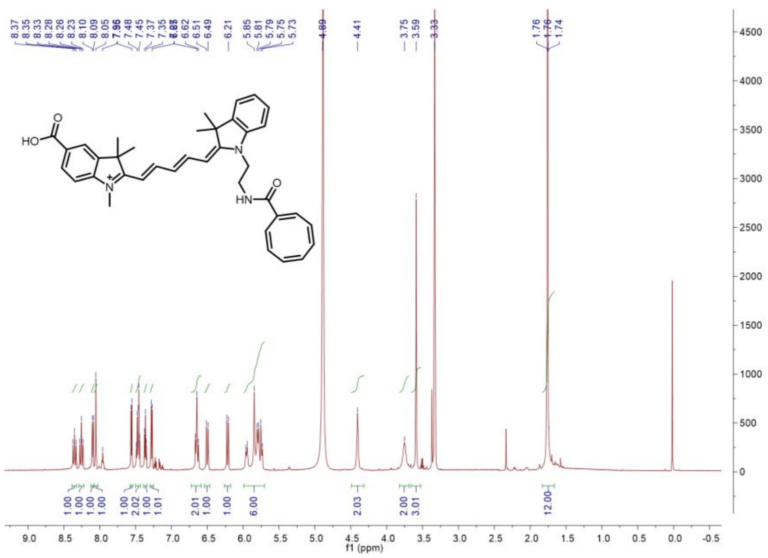


Fig.S78. The $^1$H NMR spectrum of 23 in MeOD-d$_4$.

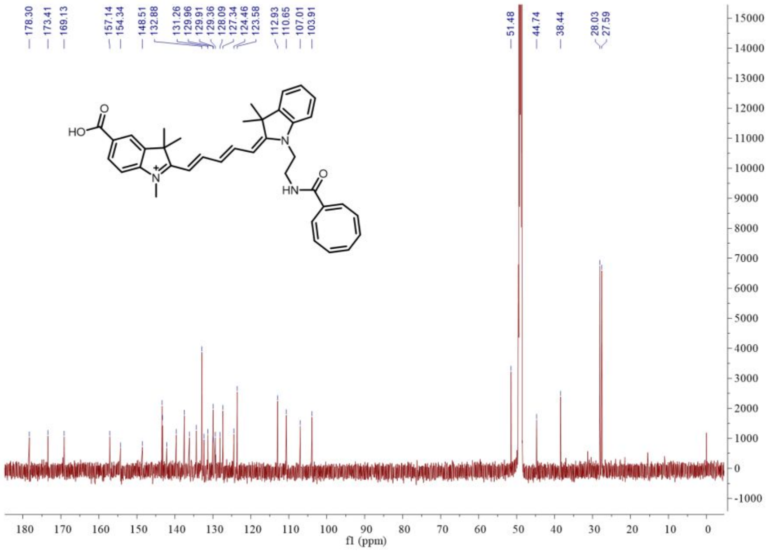


Fig.S79. The $^{13}$C NMR spectrum of 23 in MeOD-d$_4$.

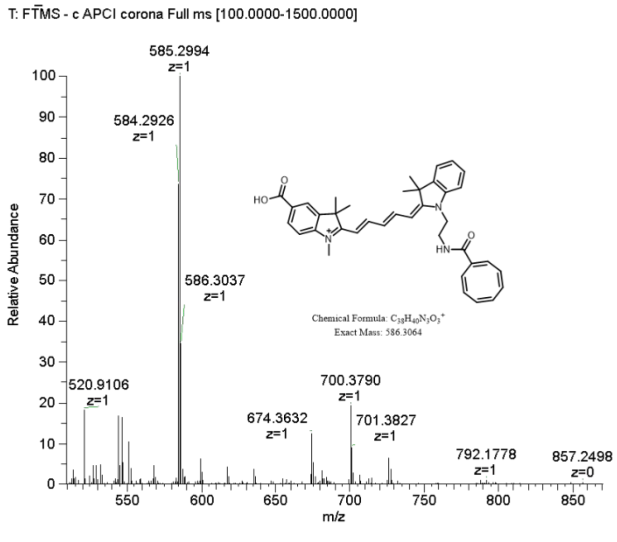


Fig.S80. The HR-MS spectrum of 23.

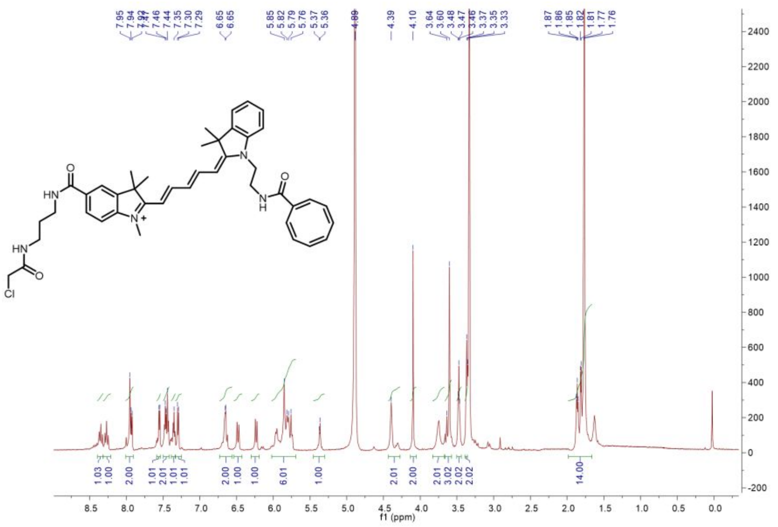


Fig.S81. The $^{1}$H NMR spectrum of HZ Mito Deep Red in MeOD-d$_4$.

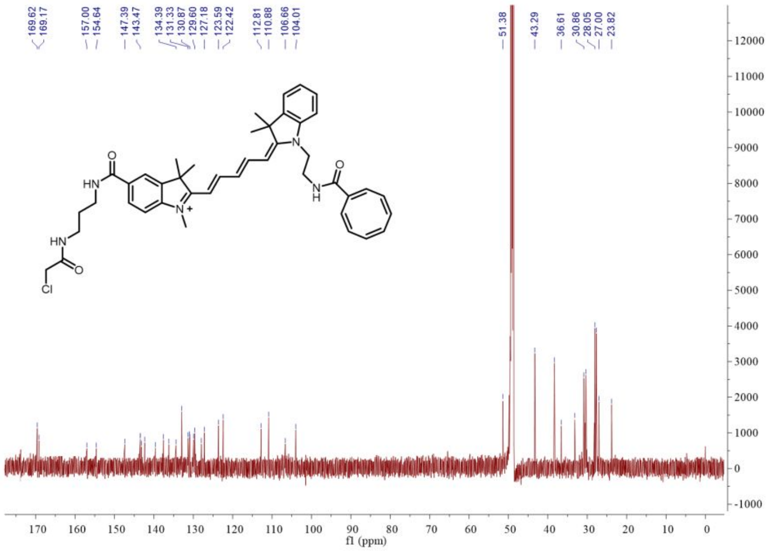


Fig.S82. The $^{1}$H NMR spectrum of HZ Mito Deep Red in MeOD-d$_4$.

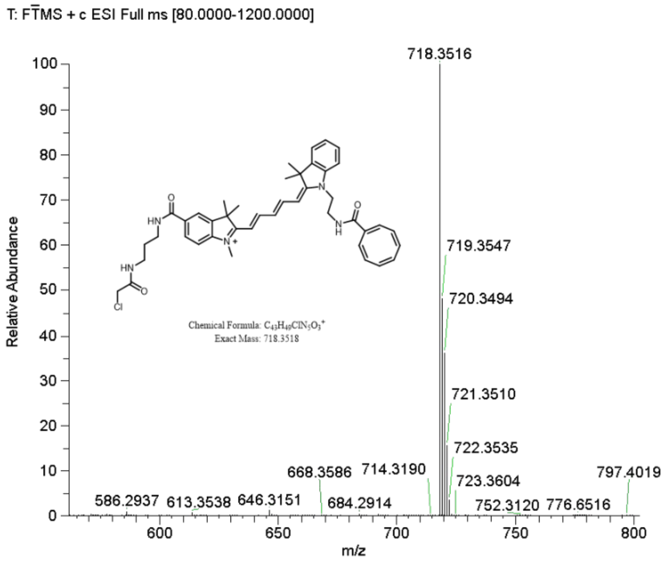


Fig.S83. The HR-MS spectrum of HZ Mito Deep Red.
